# Computational Model for Tumor Oxygenation Applied to Clinical Data on Breast Tumor Hemoglobin Concentrations Suggests Vascular Dilatation and Compression

**DOI:** 10.1371/journal.pone.0161267

**Published:** 2016-08-22

**Authors:** Michael Welter, Thierry Fredrich, Herbert Rinneberg, Heiko Rieger

**Affiliations:** 1 Theoretical Physics, Saarland University, Saarbrücken, Germany; 2 Division of Medical Physics and Metrological Information Technology, Physikalisch Technische Bundesanstalt PTB Berlin, Germany; University of Arizona, UNITED STATES

## Abstract

We present a computational model for trans-vascular oxygen transport in synthetic tumor and host tissue blood vessel networks, aiming at qualitatively explaining published data of optical mammography, which were obtained from 87 breast cancer patients. The data generally show average hemoglobin concentration to be higher in tumors versus host tissue whereas average oxy-to total hemoglobin concentration (vascular segment RBC-volume-weighted blood oxygenation) can be above or below normal. Starting from a synthetic arterio-venous initial network the tumor vasculature was generated by processes involving cooption, angiogenesis, and vessel regression. Calculations of spatially resolved blood flow, hematocrit, oxy- and total hemoglobin concentrations, blood and tissue oxygenation were carried out for ninety tumor and associated normal vessel networks starting from various assumed geometries of feeding arteries and draining veins. Spatial heterogeneity in the extra-vascular partial oxygen pressure distribution can be related to various tumor compartments characterized by varying capillary densities and blood flow characteristics. The reported higher average hemoglobin concentration of tumors is explained by growth and dilatation of tumor blood vessels. Even assuming sixfold metabolic rate of oxygen consumption in tumorous versus host tissue, the predicted oxygen hemoglobin concentrations are above normal. Such tumors are likely associated with high tumor blood flow caused by high-caliber blood vessels crossing the tumor volume and hence oxygen supply exceeding oxygen demand. Tumor oxy- to total hemoglobin concentration below normal could only be achieved by reducing tumor vessel radii during growth by a randomly selected factor, simulating compression caused by intra-tumoral solid stress due to proliferation of cells and extracellular matrix. Since compression of blood vessels will impede chemotherapy we conclude that tumors with oxy- to total hemoglobin concentration below normal are less likely to respond to chemotherapy. Such behavior was recently reported for neo-adjuvant chemotherapy of locally advanced breast tumors.

## Introduction

Adequate supply of tissue with oxygen and nutrients critically depends on the structure and function of its vasculature. For tumors to grow beyond 2 *mm* in size, new vasculature must develop, i.e. angiogenesis must occur [1]. The vasculature of solid tumors, however, is known to differ distinctly from that of surrounding normal tissue. Whereas vasculature in normal tissue is arranged in a hierarchy of arteries, arterioles, capillaries, venules and veins and grows under tight control of inter-capillary distances, tumor vasculature develops in a chaotic manner without such control, leading to spatial vascular heterogeneity. In solid tumors necrotic regions and regions of low microvessel density (*MVD*) may occur, whereas tumor blood vessels are more abundant at the tumor-host interface [2]. Tumor vessels are known to be immature, fragile, tortuous, dilated, to form (large diameter) arterio-venous shunts and often it is even difficult to distinguish arterioles and venules, i.e. the classification of tumor vessels as arterioles, capillaries and venules is no longer adequate [2–8]. In addition, tumor vessels exhibit high permeability to macromolecules [3]. The structurally abnormal tumor vasculature results in spatially and temporally heterogeneous blood flow, affecting tissue oxygenation (acute or perfusion-limited hypoxia). From intravital dorsal window microscopy on tumor models it is known that blood flow through tumor capillaries is frequently sluggish and at times may even be stationary and reverse direction. It follows that blood flow through tumors may not follow a constant unidirectional path. In addition, red blood cell (RBC) flux varies greatly among tumor vessels, many tumor vessels do not carry RBCs but contain plasma only [2–4, 6, 7]. Furthermore, tissue solid pressure, generated by proliferating cancer cells and modifications of the extracellular matrix of tumors, may compress tumor blood vessels thus reducing or impairing blood flow [9–11]. Tumor cells separated from nearby capillaries beyond the diffusion limit of oxygen suffer from chronic (diffusion-limited) hypoxia. Hypoxic tumor cells are known to be resistant to ionizing radiation, since oxygen is needed to stabilize radiation-induced DNA defects and, in addition, are considered to be resistant to some anticancer drugs. Therefore, tumor hypoxia is associated with poor prognosis, because it causes resistance to standard therapies and promotes more aggressive phenotypes [12, 13].

Because of their limited spatial resolution present-day non-invasive imaging techniques used in clinical practice do not allow to visualize tumor vasculature, blood flow and distributions of oxygen and nutrients at the capillary and cellular level, despite the diagnostic relevance, e.g. of *MVD* and the presence of hypoxic tumor cells. Therefore, computational modeling is an appropriate tool to analyze the interrelation of clinically amenable characteristics of tumor vasculature and oxygenation.

Tissue oxygenation has been measured in human tumors by various methods such as needle electrodes [14]. In this way oxygen partial pressures were found to be highly heterogeneous in tumors with median oxygen pressures below that of the host tissue. More importantly, tumors often exhibit hypoxic regions with oxygen partial pressures below 10 *mmHg*. Today, non-invasive positron emission tomography (PET) is employed clinically to image oxygenation status of tumor tissue [15]. Complementary to tissue oxygenation measurements magnetic resonance imaging (MRI) [16, 17] and tissue optical imaging, such as optical mammography assess (RBC-volume weighted) blood oxygenation of vascular segments.

Optical mammography [18–20] has been developed over the past one to two decades and a large body of data on deoxy-,oxy-, and total hemoglobin concentrations *c*_*HbD*_, *c*_*HbO*_ and *c*_*Hb*_ in normal breast tissue and breast tumors has been collected from many patients [21–24]. Because of light scattering photon trajectories through the breast are much (about 5 times) longer compared to the geometrical distance between the point of entry and point of exit of the photons, resulting in high sensitivity towards absorption. However, spatial resolution is generally poor and only average hemoglobin concentrations can be deduced. Tissue hemoglobin concentration *c*_*Hb*_ equals the sum of hemoglobin mass of each vessel segment taken over the vessel network within a selected tissue volume per unit volume of tissue and approximately reflects fractional blood volume. The ratio of oxy- to total hemoglobin concentration, called tissue blood oxygen saturation *Y*, is the average of RBC-volume-weighted true blood oxygenation taken over all vessel segments of the tissue volume selected. Tissue blood oxygen saturation reflects the balance between vascular flux of oxygen that enters the tissue, i.e. oxygen supply, and oxygen flux from the vascular network into tissue, reflecting oxygen demand. Besides tissue hemoglobin concentration, tissue blood oxygen saturation is thus another parameter characterizing tumor and host vascular networks.

On average tissue hemoglobin concentration *c*_*Hb*_ of breast tumors was found to be larger than in host tissue by a factor of 3.5 [22]. This observation can qualitatively be explained by a larger blood volume in tumors compared to their host tissue. However, tissue blood oxygen saturation in breast tumors was observed by most authors to be both above or below normal [21–23] whereas some papers reported tissue blood oxygenation in tumors to generally be below that of their host tissue [25, 26]. Up to now, there were no microscopic theoretical models available to account for tissue blood oxygenation in tumors allowing to analyze clinical data. Computational models as the one we present in this paper contain detailed information about the vasculature as well as the emerging oxygen distributions of tumors and host tissue. By coarse graining the obtained detailed information and comparing it with low resolution clinical data, one can infer potential vascular structure in the tumor of patients—with some uncertainty of course.

Mathematical modeling and numerical simulation of growth of solid tumors has been actively pursued since many years, for a recent review see e.g. [27]. In this paper 3D host vasculature is obtained by applying stochastic vessel network generation algorithms (see S1 of [28]). During tumor growth, remodeling of the host vasculature occurs involving vessel cooption, angiogenesis, vessel circumferential growth, vessel regression and collapse, simulated by corresponding stochastic vessel network remodeling processes (see S1 of [28]). In the present paper we adopted a simplified tumor growth model where a spherical tumor is expanding at constant rate. Host tissue and tumor are represented as hybrid models consisting of their vascular network surrounded by a homogeneous medium, representing host cells or tumor cells, respectively. Intravascular and extravascular oxygen transport has been studied by several authors. Oxygen dissolved in plasma, being in chemical equilibrium with oxygen bound to hemoglobin, extravasates and diffuses in tissue, where it is metabolized. Oxygenation by isolated vascular tubes has been studied theoretically in detail since the originally proposed “Krogh” model [29], see e.g. [30]. Also in several works, simplified two-dimensional tumor tissues, supplied by point-sources of oxygen, were considered, and the resulting oxygen concentration distributions were analyzed [31–37]. However, these models require appropriate intravascular O_2_ concentrations as input parameters. Previously, Goldman [38] coupled oxygen in a vascular network and tissue via boundary conditions which relate the radial transvascular oxygen flux at each point on the vessel surface to the PO2 gradient at the respective tissue location. For further reading focusing on skeletal muscle oxygenation, see [39] and the Refs. therein. Secomb and coworkers [40–43] used a Green’s function method for solving the diffusion equation in tissue, treating vessels as distributions of oxygen sources and tissue represented as a distribution of oxygen sinks. We followed the computational method of Beard [44], discretizing the diffusion equation for oxygen by a finite difference approach with source terms added locally corresponding to the surface area of nearby vessels. Our computational method is sufficiently fast to simulate tissue volumes of 0.5 *cm*^3^. Only stationary solutions are sought, ignoring transient variations of blood flow through tissue discussed above. For each vascular network generated our model allows us to calculate various biophysical and metabolic quantities, in particular partial oxygen pressure distributions in tissue, blood flow (perfusion), true vessel length weighted blood oxygen saturation distribution in the vascular network, tissue hemoglobin concentrations and tissue blood oxygen saturation, the latter two quantities being dominated by high caliber vessel. Blood vessels that set themselves apart by their diameter (approx. 100 *μm*) from the bulk of other vessels were found in corrosion casts of blood vessels of leiomyomata [45]. Moreover, in mammary carcinoma vessels of up to 200 *μm* diameter were observed [46].

Recently it was reported [47] that breast cancer patients who underwent neoadjuvant chemotherapy before surgery and were not classified as complete responders by immuno-histochemistry at the end of chemotherapy, tended to initially exhibit tumor blood oxygenation below that of host tissue. On the other hand, patients who were ranked as pathological complete responders exhibited the same tissue blood oxygen saturation on average in tumor and host tissue. Thus, tissue blood oxygen saturation may have diagnostic value for therapy control and it is important to understand what causes tissue blood oxygen saturation in tumors to fall above or below normal.

It is the aim of the present paper to gain information on host and tumor vascular networks from clinical data on tissue hemoglobin concentration and tissue blood oxygen saturation of breast tumors and their surrounding host tissue. To this end various synthetic host and tumor vascular networks were generated and tissue total hemoglobin concentration as well as tissue blood oxygenation calculated. As expected, tumor total hemoglobin concentration was always found to be larger than that of host tissue, consistent with clinical data. Within reasonable limits of metabolic rates of oxygen consumption by normal and tumorous breast tissue, simulated tumor blood oxygen saturation always fell above normal, in contrast to clinical data, indicating that increased perfusion and hence increased oxygen supply outweighs higher oxygen consumption in tumorous tissue. Tumor blood oxygen saturation was simulated below normal depending on the degree of vascular compression of (high-caliber) tumor vessels assumed to be caused by tumor solid stress. Although qualitative agreement with clinical data on tissue blood oxygenation was achieved, other processes than compression of high-caliber tumor vessels may lead to perfusion impairment in at least parts of a tumor and hence tumor blood oxygen saturation to drop below normal.

**The main part of this paper is organized as follows**: first, we briefly explain our tumor growth model including the generation of host and tumor vasculature. Then we discuss the mathematical models used to account for blood flow, intravascular and extravascular oxygen transport, which are well known [30, 40] but reviewed here for clearness and readability. Further below we describe our method to solve the obtained coupled equations describing intra-and extravascular PO2 distributions efficiently. Section ‘Theoretical models’ concludes with a description of three model variants, the results of which are presented in the following section ‘Results and Discussion’. There we initially provide examples of simulated PO2 and hematocrit distributions of host and tumor tissue, illustrating compartmentalization in tumors. Next we validate our model by comparing simulation results on host und tumorous tissue with biophysical and biochemical data available in the literature. Then we focus on clinical data on tissue hemoglobin concentration and tissue blood oxygenation of breast tumors, determining which simulation model variant provides qualitative agreement. Additionally, we correlate various simulated quantities of host and tumor that are not easily amenable to clinical measurements. Finally we discuss the limitations of our model and summarize our results in the conclusion section.

## Theoretical models

### Overview of tumor growth model for hemodynamic and oxygen transport simulations

Ideally, one would start from digitized 3D blood vessel networks of each breast cancer patient to simulate hemodynamic properties, as well as intravascular and extravascular oxygen transport in tumor and surrounding host tissue. Although 3D vascular networks of tumors grown in animal tumor models can be generated from *μ*-CT data [48, 49], such techniques are not applicable to humans. Therefore, 3D vascular trees of tumors and surrounding host tissue are algorithmically constructed, following methods developed by us previously and applied to model vasculature in melanomas and gliomas [28, 50–54]. The model described in detail in Ref. [28] represents an extension of the model used in [55] which is based on wall shear stress guided random growth of interdigitating vascular trees on a lattice. In contrast to previous work, the model of the tumor spheroid employed in this paper is very simplified.

**Fig 1:**
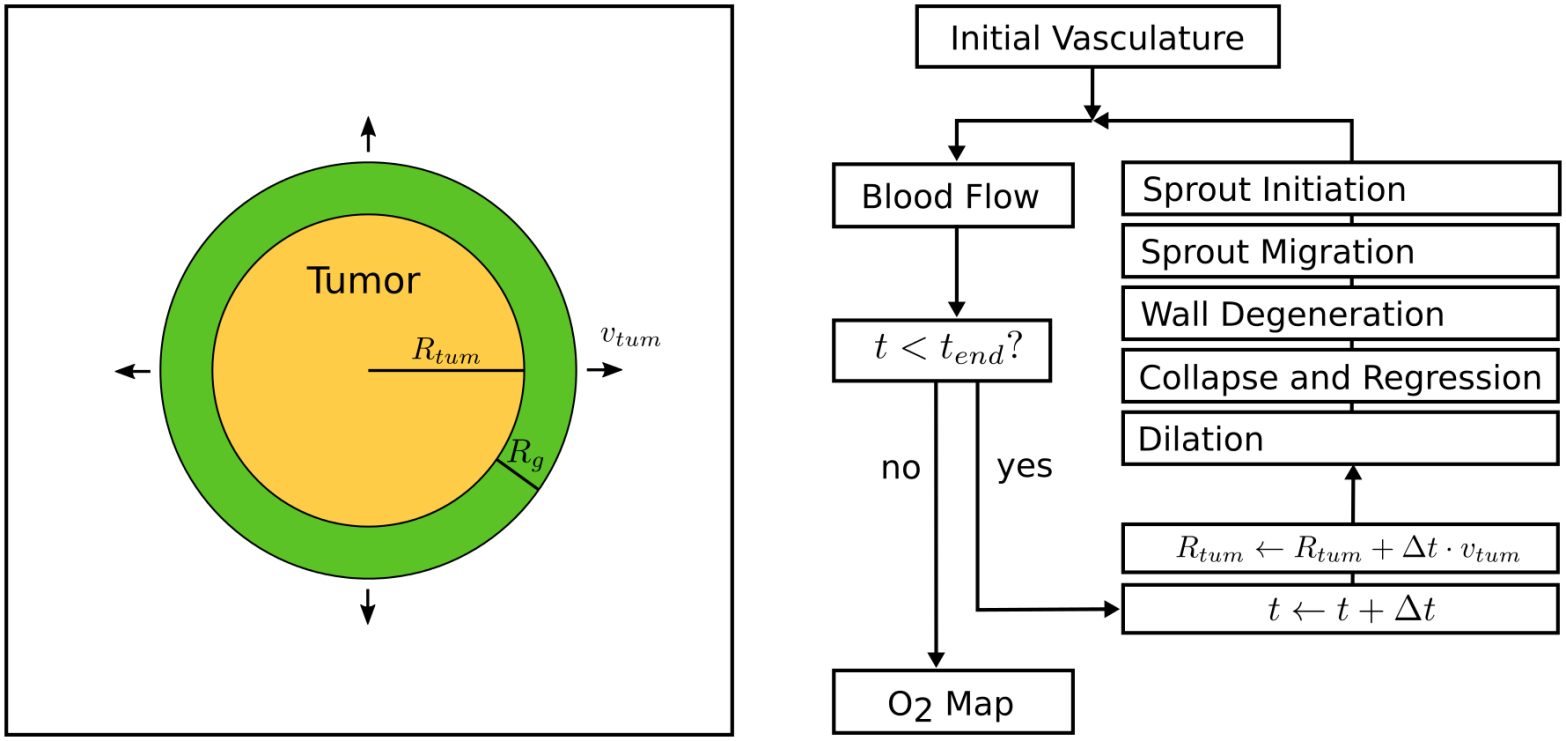
Model Overview. Left hand side: the tumor spheroid is represented by a homogeneous sphere, the radius of which *R*_*tum*_ grows at constant speed *v*_*tum*_ and is surrounded by a concentric shell of thickness *R*_*g*_, the diffusion length of vascular endothelial growth factors. Vascular remodeling is thought to take place within the tumor and surrounding concentric shell. Right hand side: block diagram outlining basic simulation procedure. The modification of the present vasculature is implemented by stochastically applying the processed depicted in Fig 2. At the end of each time step, blood flow is recomputed, time *t* and tumor radius *R*_*tum*_ incremented. The iteration steps are repeated until the final simulated time *t*_*end*_ is reached.

Fig 1 illustrates the theoretical model of tumor growth used in the present paper. Starting from synthetic host vasculature, a small spherical nucleus (*R*_*tum*_(*t* = 0) = 250 *μm*) is inserted at the beginning (*t* = 0) of the growth process into host tissue. The tumor spherically expands and its radius is assumed to grow linearly in time with speed *v*_*tum*_ = 2 *μm*/*h* up to a preselected time *t*_*end*_, taken to be 600 *h*. In real tumors, competition for space and nutrients causes confinement of cell proliferation to a few cell layers behind the invasive edge and therefore eventually leads to a linear expansion rate. The tumor tissue is assumed homogeneous. We surround the tumor with a concentric shell of thickness *R*_*g*_, within which angiogenesis, i.e. sprouting of new vessels is activated. The distance *R*_*g*_ represents the diffusion length of vascular endothelial growth factors (VEGFs), i.e. the distance that growth factors diffuse, before their concentration drops to insignificant levels due to degradation and binding. Vascular remodeling takes place during each time step delta *t* = 1 *h*, corresponding to the following biological processes: sprout initiation, sprout migration, vessel wall degeneration, vessel collapse and regression and vessel dilatation due to circumferential vessel growth. These biological processes are implemented as probabilistic addition (angiogenesis), deletion (collapse) of vessel segments, as well as probabilistic and deterministic changes of segment associated variables (e.g. wall thickness or vessel radius, see below). During each time step, we iterate over lattice sites or vessel segments as required and perform the local alterations. Since the probability for vessel collapse and regression depends on wall shear stress, blood flow through host and tumor vasculature is calculated as Poiseuille flow and updated after each time step. Furthermore, for angiogenesis (sprout initiation, sprout migration) to occur, VEGFs have to be present in tumor tissue. To this end more advanced growth models, such as the one described in [28], calculate a VEGF concentration field during each time step. For this purpose the (extravascular) oxygen concentration field is calculated in tumor tissue and hypoxic tumor cells are taken as sources for VEGF, assuming the same preset blood oxygen saturation throughout the entire vasculature, however. In the present work we focus on a realistic oxygen transport model taking the decrease of intravascular oxygen concentration due to oxygen extravasation into account. In order to reduce model complexity we assume vascular remodeling to take place within the tumor and surrounding concentric shell, i.e. assume sufficient VEGF to be within the tumor and its outer shell for angiogenesis to occur. The considerable simplification of our present tumor growth model is justified because vascular morphologies obtained in this way are consistent with data from real tumors [56, 57] and predictions of more detailed tissue models. The simulation growth process was deliberately stopped at *t* = 600 *h* when the tumor diameter had grown to ca. 3 *mm* to minimize boundary effects which would occur if the tumor was allowed to grow to the size of the simulation box.

**Fig 2:**
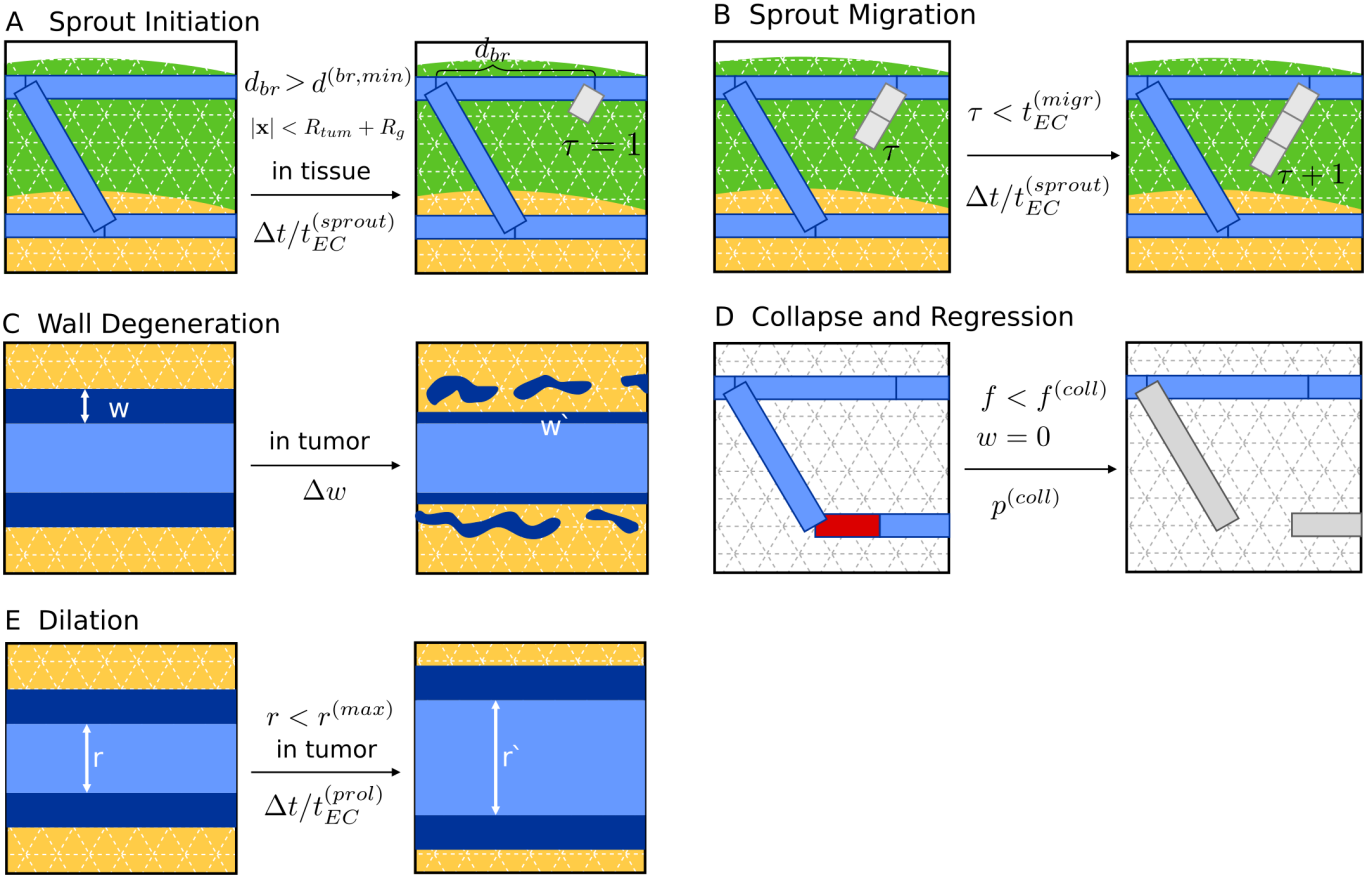
Vascular Remodeling Processes. The dynamical processes of vascular network remodeling (A-E) are illustrated by exemplary state transitions with the current state shown in the left hand side box and the resulting state on the right. Preconditions are indicated above the center arrows, and transition probabilities are denoted below, respectively. (A) depicts the start of a new sprout (gray) which can happen on any site on the network provided that given preconditions are true. Here, one of several preexisting segments (blue) is split to create a new branching point. Space occupied by the tumor spheroid (orange), and space where angiogenic sprouting may take place (green), i.e. where *R*_*tum*_ < |***x***| <*R*_*tum*_ + *R*_*g*_, is indicated by colored background. “In tissue” refers to the exterior of the spheroid, i.e. where |***x***| >*R*_*tum*_. “In tumor” means the opposite, obviously. The path length on the network to the next branching point *d*_*br*_ must be larger than the lower limit *d*^(*br*, *min*)^. The new segment is initialized with an associated life-time of *τ* = 1. (B) depicts the further extension of the sprout from (A). Additional segments inherit *τ* from the parent segment. Moreover *τ* is incremented, globally, for all sprouts once per time step Δ*t*. (C) depicts the degradation of vessel walls. The variable *w* represents the stability of the vessel wall, here depicted as varying wall thickness. It decreases continuously at the rate Δ*w*, resulting in a value of *w*′ at the next time step. In (D) an unstable vessel (red) is removed, representing occlusion of blood flow and complete disintegration. Such event is assumed to happen only to vessels with maximally degenerate walls *w* = 0 and low wall shear-stresses *f*, where *f* < *f*^(*coll*)^. The emerging dead ends (gray) trivially have *f* < *f*^(*coll*)^, and therefore collapse rapidly, resulting in a long ranged effect. Since collapses can happen anywhere, we left the background blank. (E) depicts the dilation of tumor vessels. Their radii increase at rate Δ*r* up to the upper limit *r*^(*max*)^.

By simulating tumor growth and vascular remodeling, a tumor-specific vascular network emerges at the end (*t* = *t*_*end*_) of each growth process. Because of the stochastic nature of the growth processes involved, each simulation yields a different network. Based on the networks obtained, relative blood volume, tissue perfusion, hematocrit, and tissue hemoglobin concentration *c*_*Hb*_ are calculated. In addition, using a sophisticated model of oxygenation (see below), tissue blood oxygen saturation *Y* = *c*_*HbO*_/*c*_*Hb*_ and maps of oxygen partial pressure (PO2) were simulated. Note that the assumed spheroidal tumor region generally differs in its physiology, e.g. metabolic rate of oxygen consumption, from that of the surrounding host tissue. If not stated otherwise, results for host tissue refer to the entire (unaltered) network at *t* = 0.

#### Artificial blood vessel networks in normal breast tissue

We briefly review the generation of 3D host vascular networks and refer the reader to the supplemental material S1 of Ref. [28] for details. Simulations are performed on a cubical domain with lateral length of *L* = 8 *mm*. Starting point are arterial and venous root nodes, (endpoints of feeding arterioles and draining venules) located at boundary sites of the face centered cubic lattice. Structural elements of different shape (linear, tripod) are added to randomly selected terminal branches of the vascular trees one at a time. This process is continued until further addition of vessel segments causes segments to overlap, which is forbidden by definition. In the subsequent stage of the construction scheme, trees generated in this way are pruned and extended to obtain the final network. It comprises the following steps. Firstly, nearby open ends at terminal branches are temporarily connected by vessel segments representing capillaries. Radii of capillaries, of arterial and venous terminal segments are set to 2.5 *μm*, 2.5 *μm* and 3.8 *μm*, respectively, whereas radii of parent segments are determined from Murray’s law, i.e. $r_{c}^{\alpha }=r_{a}^{\alpha }+r_{b}^{\alpha }$ with *r*_*c*_ being the radius of the parent vessel, *r*_*a*_, *r*_*b*_ the radii of the two downstream vessels and *α* = 3 has been selected. Secondly, blood flow through the temporary network is calculated, including shear wall stress of segments. With the capillaries removed additional vessel segments are stochastically attached to or removed from terminal branches with probability based on wall shear stress, corresponding to wall shear stress guided random growth of interdigitating vascular trees on a lattice [55]. This process is repeated until the number of capillaries reaches a plateau, i.e. the lattice is filled with a interdigitating trees exhibiting a homogeneous distribution of capillaries. The resulting *MVD* is determined by the lattice constant *h*_*gen*_, where *h*_*gen*_ = 130 *μm* has been selected in the present paper.

**Fig 3:**
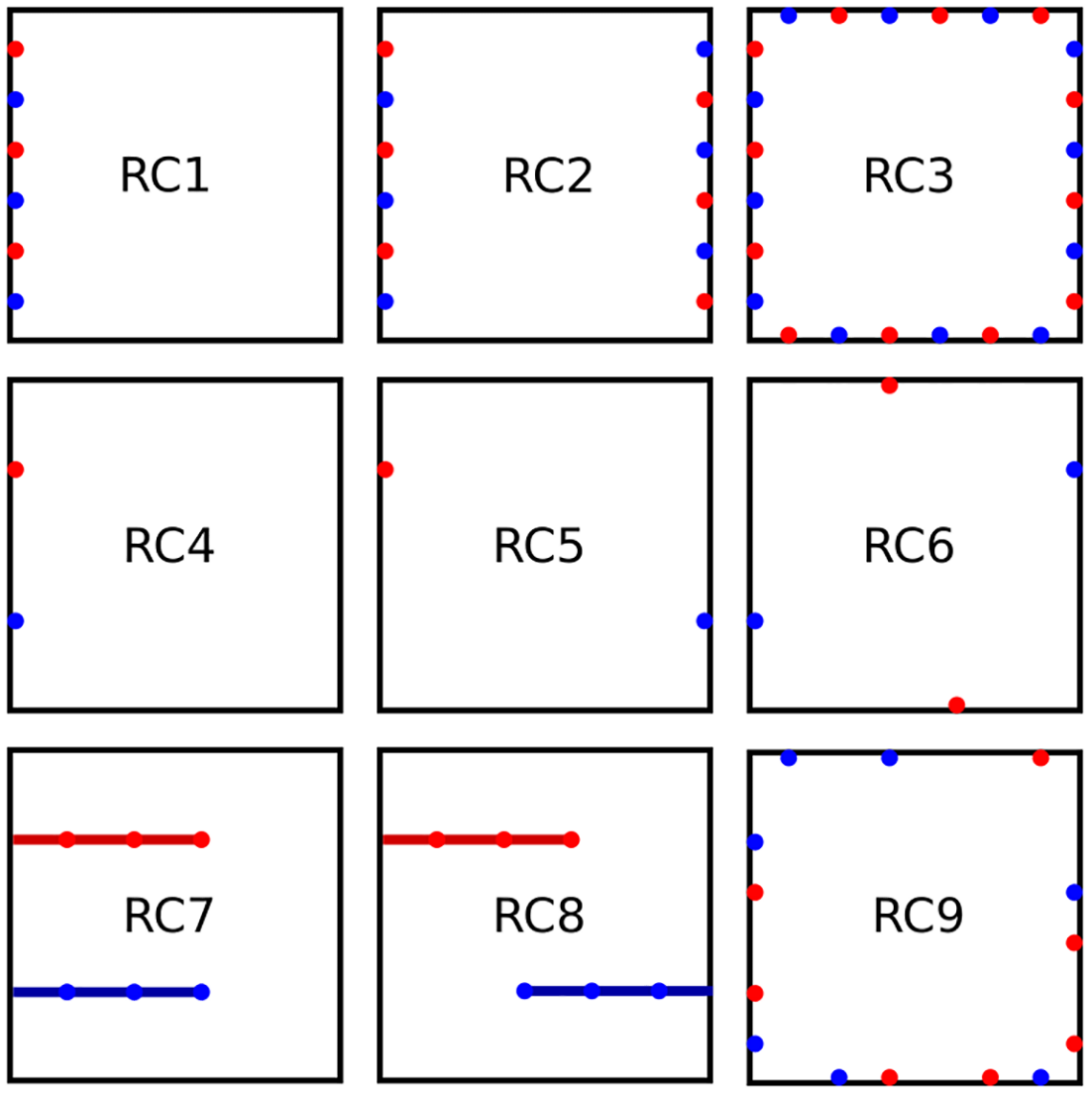
Illustration of vascular tree configurations. Schematic views from above on simulation cubes indicate vascular tree root locations for configurations denoted RC1 to RC9. In RC1 to RC3 boundary sites are occupied in alternating order either with arterial or venous nodes. In RC1 only one face of the simulation cube is used, in RC2 two opposing sides are used and in RC3 four faces are used. In RC4, RC5 and RC6 we place nodes at 33% and 66% of the length of the cube faces diagonals on the diagonals. As before, one, two and four faces are used, where in total only two (RC4, RC5) and four nodes (RC6) are placed. In RC7 and RC8 we first create fixed parent vessels protruding along the x-axis to 60% of the cube length into the interior. On each site occupied by these parent vessels a regular root node is created for further growth. Flow boundary conditions are specified at the actual inlets and outlets at the cube boundaries. In RC9 each boundary site of the lattice is occupied with a root node with probability *p*_*root*_. Before a network is created, *p*_*root*_ is drawn equally distributed between 0 and 1, however a configuration is only accepted if there is at least one arterial and one venous node.

Variations of host vessel networks among patients of a cohort were introduced by arbitrarily adopting nine root node geometries, illustrated in Fig 3, differing in location and number of arterial (venous) inlet (outlet) vessels. Starting from each root node geometry ten host vascular networks were grown which differed among each other because of the statistical nature of the growth process. Thus a total of 90 different vascular networks were generated, representing normal tissue vasculature. Using these networks we demonstrated that the specific arrangement of host vessels prior to tumor growth can play a significant role for the outcome of tumor vascular remodeling. We believe that all generated vascular morphologies are physiologically plausible. However, we did not make an effort to determine the frequency with which configurations *RC*1 − *RC*9 might occur in real breast tissue (s. ‘Limitations‘).

#### Details of vascular remodeling during growth of synthetic tumor blood vessel networks

Starting from one of the 90 vascular networks representing host tissue the associated tumorous vessel network is formed by following processes, also ilustrated in Fig 2:

1. Angiogenic sprouting: In normal tissue, lattice sites occupied by vessels, have a chance (probability $\Delta t/t_{EC}^{\left(sprout\right)}$) to spawn a sprout segment on an adjacent lattice bond provided the site is close enough to the tumor, i.e. within the growth factor diffusion distance *R*_*g*_ rather than requiring the growth factor concentration to be sufficiently high, as previously postulated [51]. Furthermore, no other branching points must be nearby, i.e. within the distance of *d*^(*br*, *min*)^. Vessels within the tumor sphere, switch to circumferential growth (s. below). The direction of sprouting is chosen randomly, which is non-critical, since the net effect is either way an increase of vascular density near the invasive edge, extending *R*_*g*_ units into normal tissue.
2. Sprout growth: sprouting vessels have a chance (probability $\Delta t/t_{EC}^{\left(sprout\right)}$) to be extended by another lattice bond. Sprouts have a total life time of $t_{EC}^{\left(migr\right)}$. When sprouts hit an existing vessel during their lifetime, they are connected to it. Sprouts that are consequently circulated become normal vessels. Otherwise sprouts are prone to collapse after their lifetime.
3. Circumferential growth: vessels switch from sprouting to circumferential growth after their residence time $t_{EC}^{\left(switch\right)}$ within the tumor. After switching to circumferential growth we assume each endothelial cell (10 × 10 *μm*^2^ surface area) at the inner wall to duplicate every $t_{EC}^{\left(prol\right)}$ hours leading to an increase of vascular radius *r*. We impose an upper limit on the radius (*r*^(*max*)^) after which circumferential growth stops.
4. Degradation of vessel wall stability: vessels possess a wall stability value *w* as an abstract measure of the state of degeneracy of their vessel wall. The stability measure *w* is initialized relating it to the wall thickness of healthy vessels [28, Suppl.1, Eq (2)]. The wall stability of tumor vessels decreases over time at the rate Δ*w* until zero. Once zero, vessels are allowed to collapse and regress (see below).
5. Vessel collapse and regression: If the wall shear stress *f* of a segment falls below a threshold (*f*^(*coll*)^) and *w* = 0, then the segment is removed from the network at probability *p*^(*coll*)^, representing collapse, occlusion and complete regression. In this study we set *p*^(*coll*)^ to 1, making collapses deterministic.

The vasculature obtained at the end of the tumor growth process (*t* = 600 *h*) comprises the part of the network altered by the tumor and the remaining normal vasculature. Parameters used for simulations of oxygen transport (s. Table 1) are to a large extent taken from [58], compiled from various sources, not specifically for breast tissue.

**Table 1:** Oxygen transport parameters.

Symbol	Unit	Value	Ref/Note
*n*		2.7	[58]
*P*_*S*50_	*mmHg*	27	[58]
*α*_*p*_	*mlO*_2_ *ml*^−1^ *mmHg*^−1^	3.1 ⋅ 10^−5^	[58] (a)
*α*_*t*_	*mlO*_2_ *ml*^−1^ *mmHg*^−1^	2.8 ⋅ 10^−5^	[58] (a)
*c*_0_	*mlO*_2_/*ml*	0.5	[60] (b)
*D*_*t*_	*μm*^2^/*s*	2410	[58]
*D*_*p*_	*μm*^2^/*s*	2750	[58]
*P*_*M*50_(*normal*)	*mmHg*	4	(c)
*P*_*M*50_(*tumor*)	*mmHg*	2	(c)
*M*_0_(*normal*)	*μlO*_2_ *ml*^−1^ *min*^−1^	3.7	(d)
*M*_0_(*tumor*)	*μlO*_2_ *ml*^−1^ *min*^−1^	14.8	(d)
*γ*	*μm*^3^ *O*_2_ *μm*^−2^ *s*^−1^ *mmHg*^−1^	0.0033 − 0.021	(e)
*η*_*plasma*_	*mPa*	1.2	[61]
*H*^(*BC*)^		0.45	
$P_{c}^{\left(BC\right)}$	*mmHg*	100	[62]
Δ*P*^(*BC*)^	*mmHg*/*μm*	1	[62]
$P_{0}^{\left(BC\right)}$	*mmHg*	55	[62]
*h*_*v*_	*μm*	4	
*h*	*μm*	40	

Shows parameters for throughout all simulations except case METAB where *M*_0_(*tumor*) is picked at random, for each simulation. *n* is the Hill-Exponent, *P*_*S*50_ is the PO2 where the the Hill-curve *S*(*P*) is 1/2, *α*_*p*_, *α*_*t*_ denote the oxygen solubilities in plasma and tissue, respectively, *c*_0_ is oxygen concentration in saturated red blood cells, *D*_*p*_, *D*_*t*_ denote the diffusion coefficients in plasma and tissue, *P*_*M*50_ denotes the M-M pressure where *M*(*P*) = *M*_0_/2, *M*_0_ denotes the maximal oxygen consumption rate, *γ* is the transvascular mass transfer coefficient. For blood flow, *η*_*plasma*_ is the blood plasma viscosity, *H*^(*BC*)^ is the inlet hematocrit, and $P_{c}^{\left(BC\right)}$, Δ*P*^(*BC*)^ and $P_{0}^{\left(BC\right)}$ are the coefficients of the radius dependency of the inlet PO2 $P^{\left(BC\right)}\left(r\right)=min\{P_{0}^{\left(BC\right)}+r\Delta P^{\left(BC\right)},P_{c}^{\left(BC\right)}\}$. The lattice constants *h* and *h*_*v*_ refer to the numerical grid on which the tissue PO2 distribution is defined, and the step width with which Eq (5) is integrated.

(a) Assuming 22.4 *l*/*mol* under normal conditions.

(b) Computed as the product of the hemoglobin binding capacity (Hüfner factor) 1.36 *ml* *O*_2_/*g* and the *MCHC* (mean corpuscular hemoglobin concentration) 0.34 *g*/*ml*.

(c) Literature [32, 37, 39, 41] values for *P*_*M*50_ range from 1 to 4 *mmHg*. *P*_*M*50_ represents a threshold below which cells become hypoxic. We lowered *P*_*M*50_ in tumor tissue by a factor of 1/2 because cancer cells are generally associated with a resistance to hypoxia.

(d) The maximal consumption rate *M*_0_ was estimated assuming zeroth order kinetics *MRO*_2_ = *M*_0_, using that *MRO*_2_ ⋅ *MTT* = *ϵc*_*Hb*, *blood*_ ⋅ *OEF* with *MTT* = *rBV*/*rBF*, *rBV* = 0.008, *rBF* = 0.06*min*^−1^ [8], *c*_*Hb*, *blood*_ = 14 *gHb*/*dl*, and *OEF* = 0.32.

(e) The lower bound is obtained for *r* = 60 *μm* and the upper bound for *r* = 3 *μm*.

### Mathematical modeling of oxygen transport in networks and tissues

#### Hemodynamic and blood oxygen transport model

The concentration of oxygen dissolved in blood plasma or in tissue is proportional to the oxygen partial pressure, i.e. *c*_*p*_ = *α*_*p*_ *P* and *c*_*t*_ = *α*_*t*_ *P*_*t*_, where *P*, *P*_*t*_ are the oxygen partial pressures in plasma and tissue, respectively, and *α*_*p*_, *α*_*t*_ are the oxygen solubilities. Conventionally, the partial pressures are measured in *mmHg* and solubilities in *mlO*_2_/*ml*/*mmHg*, yielding the oxygen concentrations expressed as oxygen gas volume at standard conditions (STP 0°C, 1 bar) per unit volume of plasma or tissue. In blood, oxygen, besides being dissolved in plasma, is bound to hemoglobin. Up to 4 molecules of oxygen can be bound per hemoglobin molecule. The chemical equilibrium between dissolved oxygen and oxygen-carrying hemoglobin depends on the number of oxygen molecules bound to hemoglobin, and the reaction is sufficiently fast, that the equilibrium state needs to be considered only. The cooperativity of oxygen-hemoglobin binding leads to the well-known oxygen dissociation curve [59], that can be approximated by the Hill equation [30]

$$\begin{array}{c} S\left(P\right)=\frac{P^{n}}{P^{n}+P_{S50}^{n}}\text{,} \end{array}$$ (1)

where *S*(*P*) is the blood oxygen saturation, *P*_*S*50_ denotes the partial pressure at 50% saturation and *n* is the Hill exponent (see Table 1). Consequently, the total blood oxygen concentration, denoted by *c*, is the sum of the concentrations of dissolved and bound oxygen.

$$\begin{array}{c} c=c_{p}+c_{b}=\alpha_{p}P+Hc_{0}S\left(P\right)\text{,} \end{array}$$ (2)

where *H* is the hematocrit, i.e. the volume fraction of red blood cells (RBCs), and *c*_0_ denotes the concentration of oxygen per unit volume of RBCs at maximal saturation (s. Table 1).

#### Oxygen transport within a single vascular cylindrical tube

The intravascular PO2 variation along the longitudinal axis of a cylindrical vascular tube is described by *P*(*x*), where *x* is the spatial coordinate. Following [40], we make the zeroth order approximation that the variation of PO2, hematocrit, and flow velocity in the radial direction within vascular tubes are negligible. This approximation reduces the problem to advection in one dimension. Radial transport, i.e. transvascular oxygen exchange is accounted for by the addition of suitable mass transfer terms (see below). Hence, the product of the oxygen concentration with the blood flow rate *q* yields the oxygen flux *ι* representing the total amount of oxygen at any given point that flows per unit time through the tube’s cross-sectional area

$$\begin{array}{c} \iota (P)=qc(P)\text{.} \end{array}$$ (3)

While blood flows along the vessel, some oxygen is continuously lost through the vessel wall. The respective transvascular flux density *j*_*tv*_ is given in units of amount of oxygen per lumen surface area and time. It causes a change in the longitudinal oxygen flux, which is expressed as

$$\begin{array}{c} \frac{d\iota }{dx}=q[Hc_{0}\frac{dS}{dP}\frac{dP}{dx}+\alpha_{p}\frac{dP}{dx}]=-2\pi rj_{tv}\left(x\right)\text{,} \end{array}$$ (4)

where the second expression is just the evaluation of *dι*/*dx*, and *r* is the vessel radius. Reordering yields the derivative of *P*

$$\begin{array}{c} \frac{dP}{dx}=\frac{-2r\pi j_{tv}\left(x\right)}{q[Hc_{0}\frac{dS}{dP}+\alpha_{p}]}\text{.} \end{array}$$ (5)

For simplicity only losses due to diffusion are considered. This is certainly a good approximation for normal vessels because oxygen is a light molecule and has a proportionately high diffusion speed. Its diffusion constant in water is ca. 2 ⋅ 10^3^ *μm*^2^/*s*. Walls of tumor vessels, on the other hand, are leaky e.g. for plasma which is why a significant convective transport is plausible. Interstitial fluid velocities of up to *v* = 1 *μm*/*s* have been reported near tumor vessels [63–66]. In order to judge whether diffusive or convective transport in tissue is dominant, we calculated the Peclet number *P*_*e*_ = *vL*/*D* which relates advective transport rate to diffusive transport rate over a certain distance *L*. A reasonable *L* is 100 *μm*, corresponding to typical intercapillary separations, yielding *P*_*e*_ = 1/20. It follows that the transport is diffusion dominated. Intravascular radial oxygen transport is in itself a complex problem. To make transvascular oxygen flux workable, the mass transfer coefficient (MTC) *γ* is introduced that relates the transvascular oxygen flux density with the PO2 difference between the plasma and tissue partial oxygen pressure *P* and *P*_*t*_.

$$\begin{array}{c} j_{tv}\left(x\right)=\gamma \cdot \left(P\left(x\right)-P_{t}\left(x\right)\right)\text{.} \end{array}$$ (6)

Since vessels are represented as lines, we also assume that *P*_*t*_ is constant over the entire circumference. Note that *γ*2*πr* = *K*^−1^, where *K* is the radial transport resistance used in [40, 41, 67]. The MTC depends on several factors like tube radius, blood oxygen saturation, hematocrit, RBC shape [30] and vessel morphology [68]. We consider a radius dependency according to a simple fit model, based on empirical data for radial oxygen transfer in vascular tubes [58, 69, 70]: This is typically characterized by the dimensionless flux density, denoted Nusselt number $Nu=2rD_{p}^{-1}\alpha_{p}^{-1}\cdot \gamma$, where *D*_*p*_ and *α*_*p*_ are oxygen diffusion coefficient and solubility in blood plasma. We found that the data presented in the Refs. cited above, ranging up to *r* = 50 *μm*, is well fit by an exponential function

$$\begin{array}{c} Nu\left(r\right)=p_{2}\left(1-\exp\left(-r/p_{1}\right)\right)\text{,} \end{array}$$ (7)

where *p*_1_ = 8 *μm* and *p*_2_ = 4.7 are fit parameters. For simplicity we omitted the dependence of *Nu* on the saturation *S*, assuming an approximate *S* = 0.9.

#### Blood flow and hematocrit in vascular networks

The vascular network is represented by a graph where edges represent vessel segments each connected to two nodes, whereas nodes may be attached to one to three vessel segments. Following the work of Pries et al. [71] we calculated blood pressure p at each node as well as blood flow rate *q* and hematocrit *H* associated with each vessel segment. The primary unknowns are the nodal pressures and segment hematocrits. The hematocrit may differ between various vessel segments due to phase separation.

The flow rates are given by Hagen-Poiseuille’s law for flow through pipes *q* = *πr*^4^Δ*p*/(8*ηl*), where *r* denotes the radius of the segment, *l* its length, *η* the (apparent) blood viscosity, and Δ*p* the difference of the blood pressure at both nodes the vessel segment is attached to. Mass conservation demands at each non-boundary node that

$$\begin{array}{c} \sum_{v}q_{v}=0 \end{array}$$ (8)

$$\begin{array}{c} \sum_{v}H_{v}q_{v}=0\text{,} \end{array}$$ (9)

where the index variable *v* runs over vessels adjacent to the currently considered node. Note that depending on context, the flow rate *q* must be taken as a signed quantity, e.g. in Eq (8) to correctly account for flow into and out of the considered node. In contrast to Ref. [71] we use pressure boundary conditions (BCs) where *p* at root nodes is set to a fixed value *p*(*root*) = *p*^(*BC*)^(*r*) depending on the radius and the type of vessel (artery or vein) (s. Eq (3) in S1 Appendix). Eq (8) and BCs lead to a system of linear equations, the solution of which yields the nodal pressures. The system is very large, of the order of 10^6^ unknowns. We found that the Conjugate Gradient algorithm with the Multilevel (ML) preconditioner from the Trilinos software library [72] solves it efficiently. The calculated nodal blood pressures impose flow directions upon vessel segments, i.e. blood flows from the end-point with higher pressure to the end with lower pressure.

The apparent blood viscosity in microvessels varies with *r* and *H* (Fahraeus-Lindqvist effect) and is expressed by decomposing it into *η* = *η*_*plasma*_ *η*_*rel*_(*r*, *H*), where *η*_*plasma*_ is the viscosity of pure blood plasma and *η*_*rel*_(*r*, *H*) is known as relative viscosity. We implemented the *η*_*rel*_ formula from more recent work of Pries et al. [73] where *η*_*rel*_ is based on in vivo data. For brevity we refer to the original publication for the expression for *η*_*rel*_.

Furthermore RBCs at arterial bifurcations tend to flow into the faster perfused branch (phase separation effect) resulting in an uneven hematocrit distribution across the network. Pries et al. [71] devised a phenomenological formula which describes the hematocrit *H* in the downstream branches of a bifurcation as a function of *q* and *r* of all adjacent vessels. For details we refer to [71]. With the help of this formula we compute the RBC flow *qH* in down stream vessels of arterial bifurcations, i.e. where one vessels splits into two downstream vessels. On the other hand, venous bifurcations have only one downstream branch where *H* is determined by Eq (9). Host vascular networks contain by default only bifurcations and the trivial case of two adjacent vessels. In the present model of tumor vascular remodeling, we reject sprout connections to other vessels that resulted in junctions with more than three vessels attached, following [50]. The hematocrit over the entire network can thus be computed with a simple depth-first search graph traversal algorithm [74] starting from the venous ends and determining the downstream *H* while backtracking.

The ansatz to solve this coupled system of equations is iterative. In each iteration, the nodal pressures are computed using the results for the vessel segment hematocrit of the previous iterations, followed by the computation of the hematocrit with new flow rates. Details can be found in [71] (see also sec. ‘Numerical solution‘).

#### Oxygen in vascular networks

The ansatz to determine the PO2 across the vascular network is similar to the method followed for calculating the distribution of hematocrit. First we consider the distribution at an arbitrarily selected node. To this end we define the sets I and O that contain adjacent up-and downstream vessels. Let further *P*_*i*_ for i∈I be the PO2 at downstream ends of adjacent inlet vessels. Then *P*_*j*_ for j∈O at the upstream ends of outlet vessels are to be determined. The main assumption made is that RBCs flowing into the junction instantly assume a common equilibrium partial pressure $\tilde{P}$, that by definition equals the PO2 at all outlets $P_{j}=\tilde{P}\;\text{for}\;j\in O$. $\tilde{P}$ is determined with the help of the oxygen mass balance

$$\begin{array}{c} \sum_{i\in I}q_{i}\left(c_{0}S\left(P_{i}\right)H_{i}+\alpha_{p}P_{i}\right)=\tilde{\iota }=\sum_{j\in O}q_{j}\left(c_{0}S\left(\tilde{P}\right)H_{j}+\alpha_{p}\tilde{P}\right)\text{,} \end{array}$$ (10)

where the left hand side is known and provides the total oxygen flux $\tilde{\iota }$ at the node under discussion. The right hand side is rearranged as

$$\begin{array}{c} \tilde{\iota }=[\sum_{i\in O}H_{i}q_{i}]c_{0}S\left(\tilde{P}\right)+[\sum_{i\in O}q_{i}]\alpha_{p}\tilde{P}\text{,} \end{array}$$ (11)

where quantities in brackets are known RBC, and blood, flow rates. Eq (11) was solved numerically for $\tilde{P}$ using a bisection search. At inlet (root) nodes, boundary conditions (BCs) must be specified. We use Dirichlet BCs where *P* = *P*^(*BC*)^ is given according to a curve *P*^(*BC*)^(*r*) depending on the arteriolar radius.

$$\begin{array}{c} P^{\left(BC\right)}\left(r\right)=\min\left(P_{0}^{\left(BC\right)}+\Delta P^{\left(BC\right)}r,P_{c}^{\left(BC\right)}\right)\text{.} \end{array}$$ (12)

For the parameter values of $P_{0}^{\left(BC\right)}$, Δ*P*^(*BC*)^, $P_{c}^{\left(BC\right)}$ see Table 1. The determination of PO2 across the entire network can be defined procedurally with the help of the depth-first search graph traversal [74] defined analogously to the propagation of hematocrit. Starting from venous root nodes, the network is traversed towards the upstream ends. Let us consider a given node while backtracking. *P* is known at the upstream ends of adjacent inlet vessels, and according to Eq (5) the down-stream *P* is determined. Hence, Eq (11) provides *P* for further backtracking downstream. This procedure holds under the caveat that *j*_*tv*_ is known, which is addressed below.

#### Oxygen in tissue

The oxygen concentration in tissue, denoted *c*_*t*_(***y***), as a function of position ***y***, is determined by the solution the following steady state diffusion equation, considering a cubic domain Ω = (0, *L*)^3^ with lateral size *L* and Neumann boundary conditions.

$$\begin{array}{c} D_{t}\nabla^{2}c_{t}-M+Q=0 \end{array}$$ (13)

$$\begin{array}{c} \nabla c_{t}\cdot n=0\;\text{on}\;\partial \Omega \text{,} \end{array}$$ (14)

where *M* is the oxygen consumption rate per unit volume of tissue and the last term *Q* represents the oxygen exchange with vessels. With the help of the solubility *α*_*t*_ this equation is rewritten in terms of the partial pressure *P*_*t*_.

$$\begin{array}{c} \alpha_{t}D_{t}\nabla^{2}P_{t}-M+Q=0 \end{array}$$ (15)

$$\begin{array}{c} \nabla P_{t}\cdot n=0\;\text{on}\;\partial \Omega \text{,} \end{array}$$ (16)

where the symbols *Q* = *Q*(*P*_*t*_,***y***) and *M* = *M*(*P*_*t*_) are redefined as functions of *P*_*t*_. They emerge from their former versions by simple scaling of their arguments by *α*_*t*_.

We model oxygen consumption according to the well-known Michaelis-Menten (M-M) model [30]. The M-M model takes into account that viable cells consume oxygen up to a maximal rate *M*_0_ even at high (excess) oxygen availability, and that consumption must drop to zero when the local oxygen partial pressure drops to zero. *M* is consequently defined as

$$\begin{array}{c} M\left(P\right)=M_{0}\frac{P}{P+P_{M50}}\text{,} \end{array}$$ (17)

where the subscript for *P* has been dropped. The partial pressure at which *M* equals 50% of its maximal rate *M*_0_ is denoted by *P*_*M*50_. *M*(*P*) increases monotonously and approaches *M*_0_ asymptotically. The advantage of the M-M model over zero-or first order kinetics is a realistic description of hyper-and hypoxic situations. Apart from their vasculature, we assume the tumor tissue and host tissue to be homogeneous and that it behaves according to the M-M relation. The selected parameters *M*_0_ and *P*_*M*50_ are thus representative of the mixture of all tissue components. This assumption is justified by the small volume of the tumor grown. Otherwise the expression for *M*(*P*) has to be weighted by the fraction of viable cells in tumor and host tissue.

To find the vascular contribution *Q*, representing sources and drains, we follow [40] where line-like sources are embedded into the volume (tissue) with a magnitude equal to *j*_*tv*_. Symbolically written, this corresponds to

$$\begin{array}{c} Q\left(y\right)=\sum_{v\in V}\int_{v}2\pi rj_{tv}\left(x,r\right)\delta \left(x-y\right)dx\text{,} \end{array}$$ (18)

where the summation is taken over all vessels and the integration along their center lines. *δ* is the Dirac delta distribution and *j*_*tv*_ is given in Eqs (6) and (7).

#### Numerical solution

The equation for the vascular oxygen distribution Eqs (5) and (11) together with the diffusion equation for tissue Eq (15) form a complicated non-linear system of equations with the partial pressures values as unknowns. The only practical way to treat this is through a numerical solution.

Previously a similar approach to ours was followed by Beard [44] who distributes the exchange flux with vessels to nearby sites of a discretization grid of the tissue domain. We justify this approach with a formulation based on a Finite Element Method (FEM) and in contrast to [44] also include hemoglobin binding dynamics. As a result we obtain a sparse system of equations which is efficiently solved by an iterative scheme.

The principal idea of the FEM is to seek an approximate solution within a finite dimensional vector space *V* that is spanned by basis functions *φ*_*i*_. Various choices are possible, e.g. Fourier bases, but the common approach is to introduce a grid and organize the *φ*_*i*_ such that their contribution is centered around a corresponding grid site ***x***_*i*_, requiring that *φ*_*i*_(***x***_*i*_) = 1 and *φ*_*i*_(***x***_*j*_) = 0 for *i* ≠ *j*. Here the choice falls for simplicity on piecewise linear (P1) tensor product bases which are defined on a regular cubic grid. Then *φ*_*i*_ reads

$$\begin{array}{ccc} \tilde{w}\left(x\right) & = & \max(0,1-|x|/h)\\ w(x) & = & \tilde{w}\left(x_{0}\right)\tilde{w}\left(x_{1}\right)\tilde{w}\left(x_{2}\right)\\ \varphi_{i}\left(y\right) & = & w(y-x_{i})\text{,} \end{array}$$

where *h* is the lattice constant and ***y*** = (*y*_0_, *y*_1_, *y*_2_) and likewise ***x*** in three dimensions. Incidentally evaluation of a function *f*(***y***) = ∑*f*_*i*_ *φ*_*i*_(***y***) with some coefficients *f*_*i*_ is equivalent to a (tri-) linear interpolation of the coefficients between grid sites that are nearest to ***y***. Hence, let the tissue PO2 be an element of *V* such that

$$\begin{array}{c} P_{t}=\sum_{j}P_{t,j}\varphi_{j}\text{.} \end{array}$$ (19)

At this point it is advisable to proceed with the treatment of the vascular system. It is by definition already represented as discrete collection of segments but these are implicitly further subdivided by the integration points that are introduced for the solution of the axial transport Eq (5) (see below). We impose an equidistant subdivision with step length of approximately *h*_*v*_ = 4 *μm*. In general, the last point needs to coincide with the end of the vessel. Therefore the true step length is adapted to the vessel length, corresponding to ⌈*l*_*v*_/*h*_*v*_⌉ + 1 integration points. Smaller values of *h*_*v*_, given all other parameters fixed, did not yield noticeably different predictions.

In the following indices *k*, *l* are used for points on the vessel network whereas *i*, *j* denote indices to sites on the tissue grid. Now it is convenient to introduce vector and matrix notations. Hence let coefficient vectors be denoted in bold face, such that ***P***_**t**_ = {*P*_*t*,*i*_} and ***P*** = {*P*_*k*_}. And let further linear operators (matrices) be denoted in bold face underlined $\underline{\cdot }$.

The solution procedure relies on an outer iteration where the tissue degrees of freedom {*P*_*t*,*i*_} and the vessel degrees of freedom {*P*_*k*_} are updated in alternating order. This can be written with the aid of two functions ***F***(***P***_**t**_) and ***F***_**t**_(***P***,***P***_**t**_) determining ***P*** and ***P***_**t**_ from the last known quantities. An iteration variable *n* is introduced denoting the current step number. This is not to be confused with *n* in the Hill-equation Eq (1).

$$\begin{array}{ccc} & & \;\text{for}\;n=1,2,3,\cdots \\ & & \;P^{\left(n+1\right)}=F\left(P_{t}^{\left(n\right)}\right) \end{array}$$ (20)

$$\begin{array}{ccc} & & \;P_{t}^{\left(n+1\right)}=F_{t}\left(P^{\left(n+1\right)},P_{t}^{\left(n\right)}\right)\\ & & \text{while}\;{\left|\right|P_{t}^{\left(n+1\right)}-P_{t}^{\left(n\right)}\left|\right|}_{\infty }>\epsilon \;\text{and}\;{\left|\right|P^{\left(n+1\right)}-P^{\left(n\right)}\left|\right|}_{\infty }>\epsilon \text{,} \end{array}$$ (21)

where ||·||_∞_ denotes the maximum norm. We set the initial guess for tissue $P_{t}^{\left(1\right)}$ to *P*_*root*,*max*_/2 on all grid points, i.e. to half the maximal inlet PO2. ***P*** needs no initial value since it is entirely determined by ***P***_**t**_. The iteration is stopped when the change between the updates becomes less than *ϵ*, where we consider *ϵ* = 0.1 *mmHg* to be small enough. Usually 200 to 400 iterations are needed for convergence, when applied to our tumor networks.

*Evaluation of **F**:* The vascular tree is traversed down stream as explained. At each vessel, first the upstream PO2 is determined according to Eq (11) or according to the boundary condition Eq (12) if it is an inlet node.

Then Eq (5) is integrated with the implicit Euler method [75]. Explicit methods are not well suited to the problem since their stability requirements [75] lead to a step size *h*_*v*_ < *const* ⋅ *q*. Since many tumor vessels are slowly perfused this would lead to sub-micrometer steps, incurring a prohibitive performance penalty. Note that the representation of *P*_*t*_ as superposition of basis functions readily provides a means to obtain its function value for the r.h.s of Eq (5). Since the bases *φ*_*i*_ have a small support this can be efficiently implemented. Moreover storage of all *P*_*k*_ values is not required. Instead we perform required operations “on the fly” when the vascular PO2 is computed in ***F***. This means we either record oxygen exchange contributions for *P*_*t*_ into the system matrix Eq (35) or compute observables for final output. It is sufficient to just store *P*_*k*_ at the upstream end of each vessel which is just $\tilde{P}$ from Eq (11).

*Evaluation of **F**_*t*_:* Our approach for the solution of the diffusion Eq (15) can be derived from the Galerkin method, where in brief, the general idea is as follows. First, the standard scalar product over the space of integrable functions over Ω is introduced (*f*, *g*) = ∫*fgd* ***x***. Furthermore instead of the true solution, an approximation within the vector space *V* is searched for. This solution is defined as *f* ∈ *V* for which the residual *R* after evaluation of the differential equation w.r.t. *f* is minimal, i.e. we look for *f* ∈ *V* such that (*R*, *R*) = *min*. Here *P*_*t*_ takes the role of *f* and we have

$$\begin{array}{c} R=\alpha_{t}D_{t}\nabla^{2}P_{t}-M\left(P_{t}\right)+Q\left(P_{t}\right)\text{.} \end{array}$$ (22)

It can be shown that minimization of ||*R*|| is equivalent to finding the *P*_*t*_ where *R* is orthogonal to *V*

$$\begin{array}{c} (R,\varphi_{i})=0\;\forall i\text{.} \end{array}$$ (23)

Eventually this results in an equation per grid site *i* in the unknowns {*P*_*t*,*i*_}. The l.h.s. of Eq (23) decomposes into the summands

$$\begin{array}{c} \alpha_{t}D_{t}\left(\nabla^{2}P_{t},\varphi_{i}\right) \end{array}$$ (24)

$$\begin{array}{c} \left(M\left(P_{t}\right),\varphi_{i}\right) \end{array}$$ (25)

$$\begin{array}{c} (Q\left(P_{t}\right),\varphi_{i})\text{,} \end{array}$$ (26)

for which algebraic expressions are needed.

The benefit of considering a finite element formulation lies in the straight forward treatment of the transvascular exchange *Q* with its Dirac delta. Also FEM makes it straight forward to use higher order elements for better accuracy. Here however we use P1 elements. Moreover, if the integration of Eqs (24)–(26) is performed by the trapezoidal rule, then the resulting discretized equations look identical to standard finite differences, s. e.g. [76]. We use this to obtain for a smooth function *g*

$$\begin{array}{c} \left(g,\varphi_{i}\right)\approx h^{3}g\left(x_{i}\right)\text{,} \end{array}$$ (27)

i.e. *g* is sampled at grid points, where we have control values *g*_*i*_ = *g*(***x***_*i*_). Likewise, one obtains for a sufficiently smooth function *f*

$$\begin{array}{c} \left(\nabla^{2}f,\varphi_{i}\right)\approx h^{3}{\left[\underline{\Delta_{h}}f\right]}_{i}+\;\text{boundary}\;\text{terms,} \end{array}$$ (28)

where

$$\begin{array}{c} {\left(\underline{\Delta_{h}}f\right)}_{i}=\sum_{j\in \text{Neighbors}\;\text{of}\;i}\left(f_{j}-f_{i}\right)/h^{2}\text{.} \end{array}$$ (29)

$\underline{\Delta_{h}}$ is the discrete Laplacian [75] known from finite differences. Neighbors of *i* denotes the 6 nearest grid neighbors. At the boundaries we modify $\underline{\Delta_{h}}$ to account for the missing neighbors [75]. The consumption term *M*(*P*_*t*_) is expanded to first order around the oxygen level from the last step. Following the above rationale, it is evaluated per grid point which yields

$$\begin{array}{c} \frac{1}{h^{3}}\left(M,\varphi_{i}\right)\approx M\left(P_{t,i}^{\left(n\right)}\right)+M^{\prime }\left(P_{t,i}^{\left(n\right)}\right)\left[P_{t,i}^{\left(n+1\right)}-P_{t,i}^{\left(n\right)}\right]\text{,} \end{array}$$ (30)

where ′ denotes the first derivative. More conveniently in matrix notation and with dropped super scripts, we write for the right hand side of Eq (30)

$$\begin{array}{c} \mathrm{MA}+\mathrm{MB}\;\underline{I}\;P_{t}\text{,} \end{array}$$ (31)

where $\underline{I}$ denotes the identity, and

$$\begin{array}{c} MA_{i}=M\left(P_{t,i}^{\left(n\right)}\right)-M^{\prime }\left(P_{t,i}^{\left(n\right)}\right)P_{t,i}^{\left(n\right)} \end{array}$$ (32)

$$\begin{array}{c} MB_{i}=M^{\prime }\left(P_{t,i}^{\left(n\right)}\right) \end{array}$$ (33)

The last contribution is derived by expanding Eq (26). For better readability, the super scripts are dropped again. Also the dependence of *r* and *γ* on the current vessel *v* is implied but for readability not indicated.

$$\begin{array}{ccc} \left(Q\left(P_{t}\right),\varphi_{i}\right) & = & \int_{\Omega }\sum_{v\in V}\int_{v}2\pi rj_{tv}\delta \left(x-y\right)\varphi_{i}\left(y\right)dxdy\\ & = & \sum_{v\in V}\int_{v}2\pi rj_{tv}\varphi_{i}\left(x\right)dx\\ & = & \sum_{v\in V}\int_{v}2\pi r\gamma \left[P\left(x\right)-P_{t}\left(x\right)\right]\varphi_{i}\left(x\right)dx\\ & = & \left[\sum_{v\in V}\int_{v}2\pi r\gamma P\left(x\right)\varphi_{i}\left(x\right)dx]-[\sum_{j}\sum_{v\in V}\int_{v}2\pi r\gamma P_{t,j}\varphi_{j}\left(x\right)\varphi_{i}\left(x\right)dx\right] \end{array}$$ (34)

The expression given in Eq (34) approximates the total volume rate of oxygen that extravasates from the entire vascular system into the tissue volume associated with the basis function *φ*_*i*_. Now the integral over the vessel network is approximated with a Riemann sum based on the integration points {*P*_*k*_} from step Eq (20). Each integration point *k* represents a finite length segment Δ*x*_*k*_ = *h*_*v*_ except at the end points of each segment where Δ*x*_*k*_ = *h*_*v*_/2. In order to achieve good accuracy, we require *h*_*v*_ < <*h*. Else, the integration kernel 2*πγφ*_*i*_ or 2*πγφ*_*i*_ *φ*_*j*_ will be under-sampled. This limitation could be removed to some degree by interpolation of the numerical solution of *P*(*x*), decoupling it from the integration quadrature formula. From Eq (34) one obtains for the total volume rate of extravasated oxygen per cell volume

$$\begin{array}{c} \frac{1}{h^{3}}\left(Q\left(P_{t}\right),\varphi_{i}\right)\approx \underset{QA_{i}}{\underset{︸}{\sum_{k}\frac{\Delta x_{k}2\pi r}{h^{3}}\gamma P_{k}\varphi_{i}\left(x_{k}\right)}}-\sum_{j}P_{t,j}\underset{QB_{ij}}{\underset{︸}{\left[\sum_{k}\frac{\Delta x_{k}2\pi r}{h^{3}}\gamma \varphi_{j}\left(x_{k}\right)\varphi_{i}\left(x_{k}\right)\right]}}\text{.} \end{array}$$ (35)

The factor Δ*x*_*k*_2*πr*/*h*^3^ is intuitively understood as vessel surface area per cell volume associated with vessel centerline integration step *k*. Finally, the completely assembled system of equations reads

$$\begin{array}{c} {}[\alpha_{t}D_{t}\underline{\Delta_{h}}-\mathrm{MB}\;\underline{I}-\underline{\text{Q}\;\text{B}}]P_{t}=\mathrm{MA}-\mathrm{QA}\text{.} \end{array}$$ (36)

It is easy to see that this is a sparse symmetric system. The Laplacian gives rise to 6 off-diagonal entries. The operator $\underline{\text{Q}\;\text{B}}$ gives rise to 26 off-diagonal entries corresponding to the nearest and second nearest grid neighbors. For points which are spaced further apart, *φ*_*i*_ and *φ*_*j*_ have no overlap so their product in Eq (35) becomes zero.

In order to improve the efficiency we approximate *P*_*t*_(***x***_***k***_) with the value from the current site *P*_*t*,*i*_. This approximation is justified physically since the oxygen diffusion length considerably exceeds the lattice spacing *h*. Consequently, $\underline{\text{Q}\;\text{B}}$ is diagonalized such that the new version, now denoted $\tilde{\underline{\text{Q}\;\text{B}}}$, reads

$$\begin{array}{c} {\tilde{Q\;B}}_{ij}=\sum_{k}\frac{\Delta x_{k}2\pi r}{h^{3}}\gamma \varphi_{i}\left(x_{k}\right)\delta_{ij}\text{.} \end{array}$$ (37)

This is a sparse linear system where the only off-diagonal terms stem only from the Laplacian, removing the off-diagonal entries corresponding to the second nearest neighbors. We solved these systems with an algebraic multigrid method (Trilinos ML [72]). We found the difference between the $\tilde{\underline{\text{Q}\;\text{B}}}$ and $\underline{\text{Q}\;\text{B}}$ solutions negligible.

The computational cost of our method scales well in the number of discretization points since the obtained sparse systems can be solved in *O*(*n* log *n*) time. In contrast the Green’s functions method [41] gives rise to dense systems and is therefore much more computationally demanding.

We take grid constants *h* of 40 *μm*, which is rather coarse for resolving the approximately exponential decay of the O_2_ concentration around isolated tumor blood vessels. The therewith associated diffusion range can be estimated, assuming zeroth order kinetics in one dimension, by $l_{diff}=\sqrt{2D\alpha_{t}P_{t}/M_{0}}$. This estimate is obtained from *dP*_*t*_(*x*)/*dx* = −*M*_0_/*Dα*_*t*_, under the boundary conditions that *P*_*t*_(0) = *P*_0_, *dP*_*t*_/*dx*(*l*_*diff*_) = 0, and *P*_*t*_(*l*_*diff*_) = 0. We obtain, *l*_*diff*_ = 295 *μm* in normal tissue, and 148 *μm* in tumor, using the parameters in Table 1 and assuming that *P*_0_ = 40 *mmHg*. In two dimensions, the Krogh model for a capillary of 3 *μm* radius, yields approximately *l*_*diff*_ = 150 and 80 *μm* in normal and tumor tissue, respectively. However, we do not use Fick’s law as in *j*_*tv*_ = −*Dα*_*t*_∇*P*_*t*_ to determine the amount of extravasated oxygen. Instead, *j*_*tv*_ is computed from the difference between *P* and *P*_*t*_ at the vessel center line. Therefore the result does not depend critically on the precise resolution of tissue *PO*2 gradients.

We repeated our calculations with a grid constant *h* of only 20 *μm*. As a result, the peaks of the calculated *P*_*t*_ distributions are better resolved. Concomitant changes in the predicted averages are rather small. For instance we obtain lower average *OEF* (0.1 versus 0.11), lower *P*_*t*_ (25 vs. 28 *mmHg*—the most significant change), and higher *Y* (0.75 vs. 0.73), in tumors of case CMPR. However, these variations are well within experimental uncertainties. Therefore, our overall results and conclusions are left unaltered.

In Fig D in S1 Appendix we investigated the convergence of the on-axis oxygen partial pressure *P*(*x*) with decreasing lattice constant *h* and conclude that at *h* = 40 *μm* the accuracy is between 3 to 13% depending on the location along the vessel. Differences of the absolute values of the intravascular and extravascular partial oxygen pressures *P*(*x*) and *P*_*t*_(***y***) with respect to the Green’s function technique of Secomb et al. [41] are illustrated in Fig C in S1 Appendix. Overall, we estimate uncertainties of absolute intravascular and extravascular partial oxygen pressures to be in the order of 10 – 20%.

### Simulation setup

#### Expressions for tissue hemoglobin concentration and tissue blood oxygen saturation

For a comparision of simulated tissue hemoglobin concentration *c*_*Hb*_ and tissue blood oxygenation *Y* with clinical data we provide expressions for these quantities based on host and tumor vascular networks. Corresponding expressions for other quantities of interest are given in S2 Appendix.

$$\begin{array}{c} c_{Hb}=MCHC\cdot rRBCV=\frac{MCHC}{\left|\Omega \right|}\sum_{v\in V}\pi r_{v}^{2}l_{v}H_{v} \end{array}$$ (38)

$$\begin{array}{c} Y=\frac{c_{HbO}}{c_{Hb}}=\sum_{v\in V}\{\frac{\pi r_{v}^{2}H_{v}l_{v}}{\sum_{u\in V}\pi r_{u}^{2}l_{u}H_{u}}\frac{1}{l_{v}}\int_{v}S_{v}\left(x\right)dx\}\text{,} \end{array}$$ (39)

where V is the set of vessel segments of host or tumor tissue, *MCHC* is the mean corpuscular hemoglobin concentration, |Ω| the relevant tissue volume, *r*_*v*_, *l*_*v*_, *H*_*v*_, *S*_*v*_(*x*) designate radius, length, hematocrit and blood oxygen saturation of vessel segment *v*. In Eq (39) integration of blood oxygenation is taken over the length of the vessel segment.

Tissue total hemoglobin concentration *c*_*Hb*_ is the sum over the hemoglobin mass of all vessel segments per unit volume of tissue. Tissue blood oxygenation *Y* is the average of RBC-volume-weighted blood oxygen saturation taken over all vessel segments. It follows that high-caliber vessels contribute dominantly to *c*_*Hb*_ and *Y*, being less sensitive to the capillary plexus.

#### Simulation model variants

In the following we discuss three different scenarios denoted BASE, CMPR and METAB. Cases BASE and METAB use the same tumor vascular networks (*t* = 600 *h*), but differ in their assumptions made to calculate tissue metabolic rate of oxygen consumption variables. CMPR, on the other hand, augments the processes that cause remodeling of the tumor vasculature by accounting for vascular compression by solid pressure, leading to tumor vascular networks at *t* = 600 *h* that differ from the networks of the scenarios BASE and METAB.

**Case BASE** This is our base case with parameters for growth of breast tumor vasculature listed in Table 2. Blood flow, hematocrit distribution, blood and tissue oxygen concentrations were calculated using each of the 90 realizations of tumor vasculature (*t* = 600 *h*) together with parameters related to oxygen transport given in Table 1. The maximal oxygen consumption rate of tumor tissue was assumed amounting to *M*_0_(*tumor*) = 4 ⋅ *M*_0_(*normal*) and *P*_*M*50_(*tumor*) was assumed equal to 1/2 ⋅ *P*_*M*50_(*normal*), reflecting the enhanced need for oxygen of proliferating tumor cells. Reports on measured oxygen consumption of solid tumors are scarce in the literature. We include a recent report on metabolic rate of oxygen consumption of viable multicellular spheroids of human colorectal carcinoma cells [77] indicating that *M*_0_(*tumor*) might even be underestimated. Compared to previous simulations concerning melanomas [28], changes in the tumor growth parameters (s. Table 2) include *d*^(*br*,*min*)^, governing the *MVD* near the tumor rim, *f*^(*coll*)^, and *r*^(*max*)^ determining the central *MVD* and *rBV*. Other parameters such as the sprouting time $t_{EC}^{\left(sprout\right)}$, and vessel wall degradation rate Δ*w* are unaltered.

**Table 2:** List of parameters for tumor growth.

Parameter	Value	Unit	Description
*R*_*tum*_(*t* = 0)	250	*μm*	Initial tumor radius
*v*_*tum*_	2	*μm*/*h*	Tumor radius expansion rate
*R*_*g*_	200	*μm*	Growth factor diffusion range
$t_{EC}^{\left(switch\right)}$	12	*h*	Circumferential growth switch delay
$t_{EC}^{\left(sprout\right)}$	2	*h*	Time between sprouting events
$t_{EC}^{\left(migr\right)}$	50	*h*	Sprout activity duration
*d*^(*br*,*min*)^	80	*μm*	Sprout sites minimum separation
$t_{EC}^{\left(prol\right)}$	144	*h*	Endothelial cell proliferation time
*r*^(*sprout*)^	2.6	*μm*	Initial sprout vessel radius
*r*^(*max*)^	10 *, 14 **	*μm*	Maximum dilatation radius
*p*^(*coll*)^	1		Vessel collapse probability
Δ*w*	0.05	*μm*/*h*	Vessel stability (*w*) decrease rate
*f*^(*coll*)^	0.25	*Pa*	Critical wall shear stress
*h*_(*gen*)_^†^	130	*μm*	Lattice constant of initial network generation

* case BASE.

** case CMPR. Increased maximal dilatation radius compensated for by overall compression of tumor vessels (see text).

^†^ The lattice constant *h*_(*gen*)_ corresponds to the vessel segment length in the initial (*t* = 0) networks. Simulation of tumor growth and vascular remodeling is performed at only 10 *μm* lattice spacing. This is possible since segments of the initial *t* = 0 lattice coincide with bonds and sites of the finer 10 *μm* lattice.

**Case CMPR: Vascular compression** We allowed for compression of tumor vessels during tumor growth by extravascular stress on simulated tissue blood oxygen saturation *Y*_*tum*_ based on the final *t* = 600 *h* tumor vasculature. Compression of tumor vessels will change the relation of *Y*_*tum*_ and the tissue blood oxygen saturation of the surrounding normal breast tissue *Y*_*norm*_ obtained from the corresponding vascular network at *t* = 0. Elevated interstitial fluid pressure (IFP) is known to be a hallmark of tumors [10], primarily caused by the increased leakiness of tumor vessels and the lymph drain being impaired in solid tumors. Yet the precise mechanisms by which extravascular pressure compresses tumor vessels are still under debate [78, 79]. The uncontrolled proliferation and growth of tumor cells within a restricted volume generates extravascular solid stress. Solid stress accumulates within the tumor volume through an increase in tumor cell density and hyperproduction of interstitial matrix molecules such as collagen and hyaluronan [9–11]. Collagen fibers are resistant to tensile stress whereas hyaluronan resists compression thus transferring stress on tumor vessels. On the other hand water-loaded hyaluronan can be considered to be part of the relative immobile fluid phase of the interstitial fluid, contributing to IFP apart from contributions of the freely mobile fluid phase. Furthermore, solid stress can be exerted on the tumor volume by the surrounding normal tissue. It was estimated that solid stress becomes significant for tumor volumes larger than 0.065 *mm*^3^, corresponding to a sphere of *R*_*tum*_ = 250 *μm* [80]. A detailed simulation of solid stress during progression of the tumor is beyond the scope of the present paper. Therefore we assume that all vessels of the tumor vasculature experience compression during the growth process, i.e. a reduction of their radius by a constant factor. The fundamental difference to the case BASE is that in this way high-caliber vessels experience a reduction in radius, which cannot be achieved by the previous model that predicts either growth or complete removal of vessels. Hence we introduce a new “compressed” vessel radius, denoted $\tilde{r}$ which enters the blood flow and oxygen computation. We consider a function *ψ*(|***x***|) of the spatial coordinate ***x*** that represents the magnitude of compression relative to the base radius, depending on the distance from the tumor center |***x***|. Therefore we define $\tilde{r}=\psi \cdot r$. We let *ψ* describe a linear transition between full compression of magnitude 1 − *ξ*_*cpr*_, in the tumor center and zero in normal tissue. Thus, vascular radii $\tilde{r}$ within the tumor, with the exception of a small transition zone of width *w*_*cpr*_, are reduced relative to *r* by a factor of *ξ*_*cpr*_. Since compression affects all vessels, and to obtain a common mean capillary diameter of cases BASE and CMPR, *r*^(*max*)^ was increased in case CMPR from *r*^(*max*)^ = 10 *μm* to 14 *μm*. This compensation helps to separate the effect of compression of large vessels from an overall compression of all radii.

$$\begin{array}{c} \psi \left(x\right)=\{\begin{array}{cc} \xi_{cpr} & \text{for}\;x<R-w_{cpr}\\ 1 & \text{for}\;x>R+w_{cpr}\\ \xi_{cpr}-\frac{x-R+w_{cpr}}{2w_{cpr}}\cdot \left(\xi_{cpr}-1\right) & \text{else,} \end{array} \end{array}$$ (40)

where *R* = *R*_*tum*_ is short for the tumor radius and *w*_*cpr*_ is assumed 200 *μm*. We assume that varying degrees of compression are present in the tumors of the patient cohort due to phenotypical differences from tumor to tumor or differences in the micro environment of the host. Therefore a new *ξ*_*cpr*_ was drawn for each simulation from a uniform distribution over the interval between 0.5 and 1. The lower bound of 0.5 was deliberately chosen since it appears to be the lower bound of what was reported for experiments, i.e. relief of stress resulted in two times increase of vessel diameters [81].

**Case METAB: Variation of tumor oxygen consumption rate** In contrast to case CMPR scenario, METAB uses the identical tumor vascular networks as case BASE. However, rather than selecting the same maximal oxygen consumption rate *M*_0_ for all tumors as in scenario BASE we consider variations of the maximal tumor oxygen consumption rate *M*_0_ among various tumors reflecting phenotypical differences. In this way we probe the impact of tumor tissue oxygen consumption versus tumor vascular restructuring on intra-and extravascular oxygen distributions. Therefore, *M*_0_ / (*ml* *O*_2_/*ml*/*min*) is drawn from a lognormal distribution with *mu* = *log*(0.0149) and *sigma* = 0.3 on a tumor by tumor basis, yielding a median *M*_0_ identical to case BASE and a standard deviation of 32% of the median. The oxygen consumption rate of normal tissue is kept at its original value (s. Table 1). Furthermore oxygen partial pressures *P*_*M*50_ were kept unchanged.

## Results and Discussion

### Tumor compartmentalization and contributions of tumor core to hemoglobin concentration, fractional blood volume and tissue blood oxygenation

**Fig 4:**
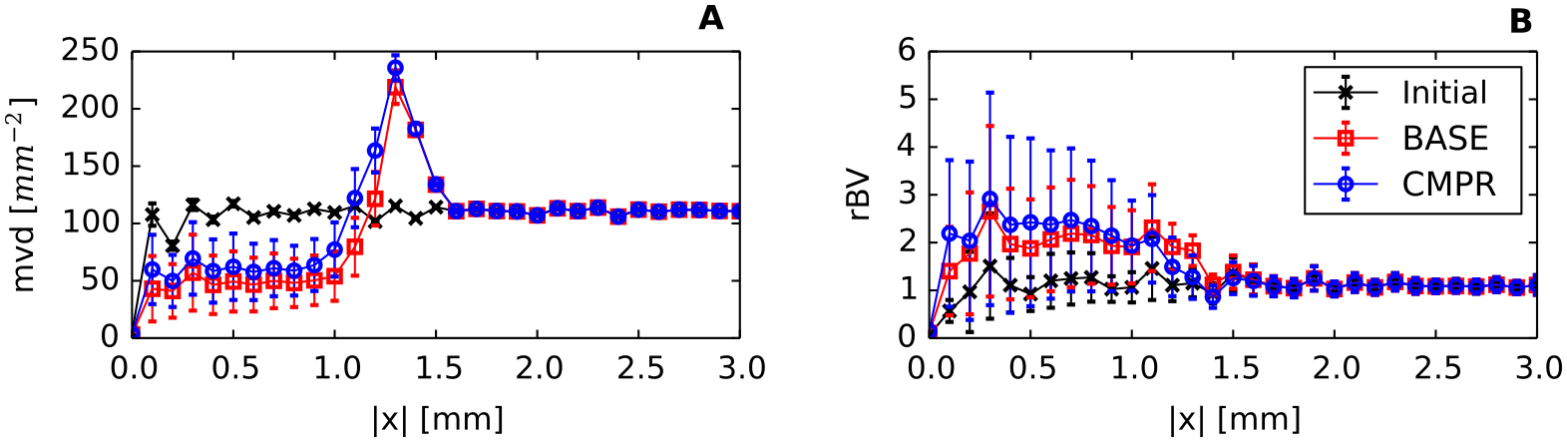
Radial distributions of the microvascular density, *MVD*, and the relative blood volume, *rBV*. The simulation box was divided in 100 *μm* thick concentric shells over which the regional *MVD* and *rBV* were determined to obtain radial profiles depending on the distance from the center |*x*|. Plotted is the ensemble mean of these profiles, where the error bars display the standard deviation (STD). As can be seen, a compartmentalization into varying degrees of vascularization (*MVD*) and vascular dilatation (*rBV*) exist which is typical for Melanoma [56] and Glioma [57]. Fig 4 provides evidence that these features are also present in breast tumors. The STD of *MVD* further shows that the *MVD* of the tumor core is below normal in most realizations of the ensemble which conforms to our expectations for tumors. Oscillations are an artifact of the confinement of the vessels to a lattice.

Using the model parameters listed in Table 2 together with the root node geometries illustrated in Fig 3 we simulated vascular networks corresponding to normal breast tissue (*t* = 0, “initial”) and tumors (*t* = 600*h*, case BASE, case CMPR). As can be seen from Fig 4, tumor vascular networks are highly compartmentalized, comprising a central core with low *MVD* and a highly vascularized peripheral shell of a few hundred micrometer thickness, consistent with previous results [28, 50–54]. The arterio-venous organization is lost, and the remaining high caliber vessel protruding into and through the tumor are connected with a chaotic network of dilated capillaries. Although central tumor *MVD* is lower than normal (s. Fig 4A), relative blood volume rBV is above normal within the tumor core (s. Fig 4B). It follows that vessels crisscrossing the tumor core are predominantly of high caliber. As was mentioned above, all vessel segments contribute to the average blood oxygen saturation 〈*S*〉 proper (s. S2 Appendix), independent of their volume.

**Fig 5:**
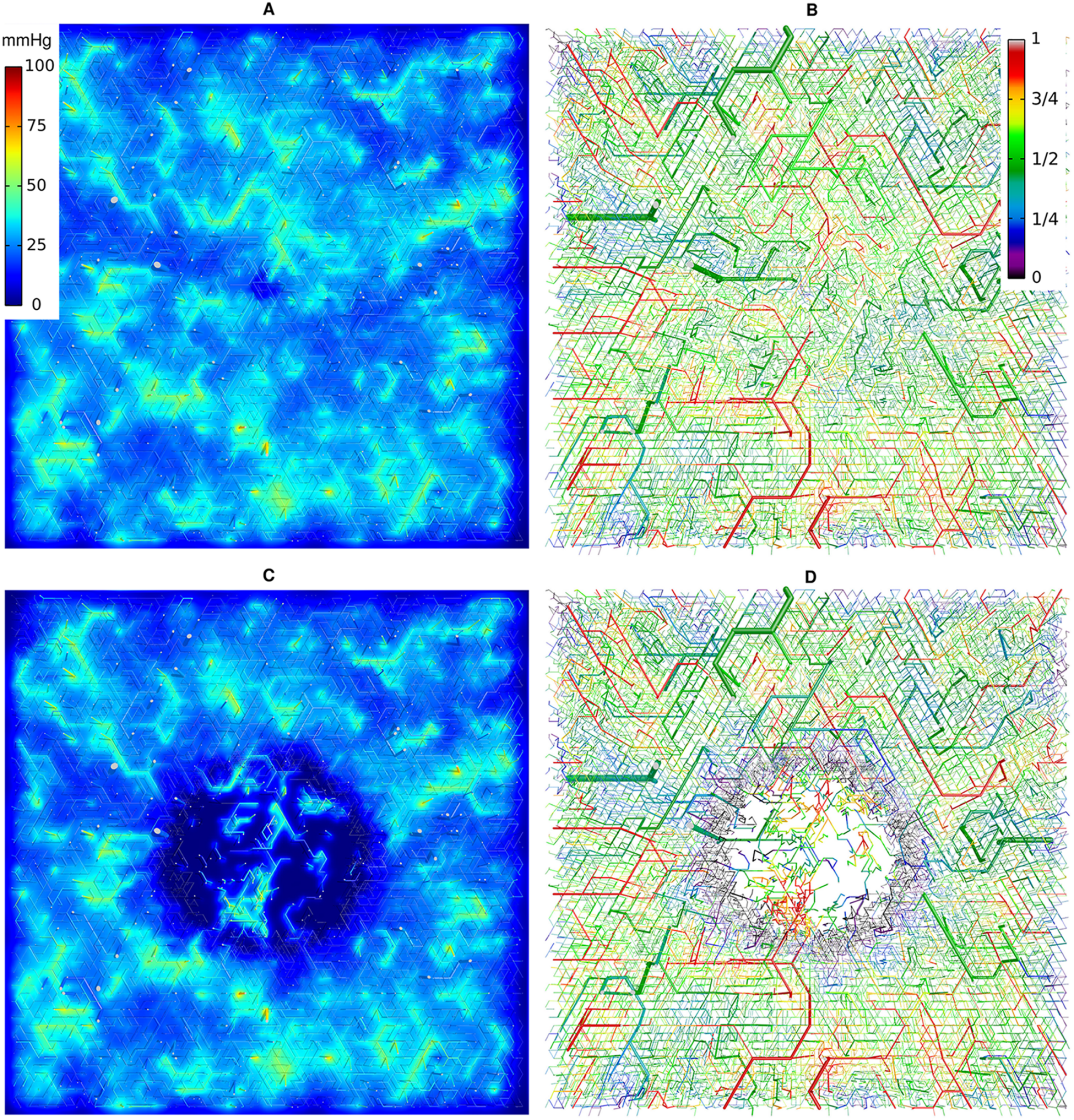
Blood oxygen saturation and oxygen partial pressure corresponding to case CMPR, root node configuration RC9. The left column (A, C) shows the PO2 of vessels, *P* and of tissue, *P*_*t*_ in a slice through the center of the simulation domain. The camera is looking vertically onto the horizontal cutting plane so that the whole lateral extent of 8 *mm* is shown. The vessel network is visualized as a collection of cylinders that have been truncated 100 *μm* above and below the central plane. The resulting cross sectional areas are light grey. Otherwise the color code indicates the PO2, *P*, of vessels and *P*_*t*_ of tissue, respectively. In the right column (B, D), only the network is shown color coded by saturation *S*. The top row (A, B) shows the initial state at t = 0. The bottom row (C, D) shows the final state at *t* = 600 *h*.

Fig 5 provides examples for vascular networks corresponding to tumor model CMPR, and root node geometry RC9 (cf. Fig 3). Distributions of partial oxygen pressure (PO2) of blood vessels, *P*, and of tissue, *P*_*t*_, as well as corresponding distributions of blood oxygen saturations *S* are shown at the start (*t* = 0, top row) and at the end (t = 600 h, bottom row) of the growth process. The other cases (BASE, METAB) and root node geometries exhibit similar looking distributions of PO2 and blood oxygen saturation *S* in normal tissue and tumor periphery. However, the vascularization at the tumor center can be lower or higher than for the example shown, which is accompanied with varying degrees of oxygenation. We made sure that in good approximation cases BASE and CMPR exhibited similar radial *MVD* and *rBV* profiles. This requirement is critical since we aim to analyze the effect on tissue blood oxygen saturation *Y* by varying the compression factors *ξ*_*cpr*_ among the ensemble of 90 tumor vascular networks (see below), while retaining agreement with clinical data on *MVD* and *rBV*. We consider details of the oxygen distribution shown for instance in Fig 5C. Immediately obvious, we see severe hypoxia at the tumor center due the sparse vasculature there. As one would expect, *P*_*t*_ is highest near vessels and falls off into non-vascularized spaces over a scale of 100 to 200 *μm*, corresponding to the oxygen diffusion-range.

### Redirection of hematocrit modulates blood oxygen saturation

**Fig 6:**
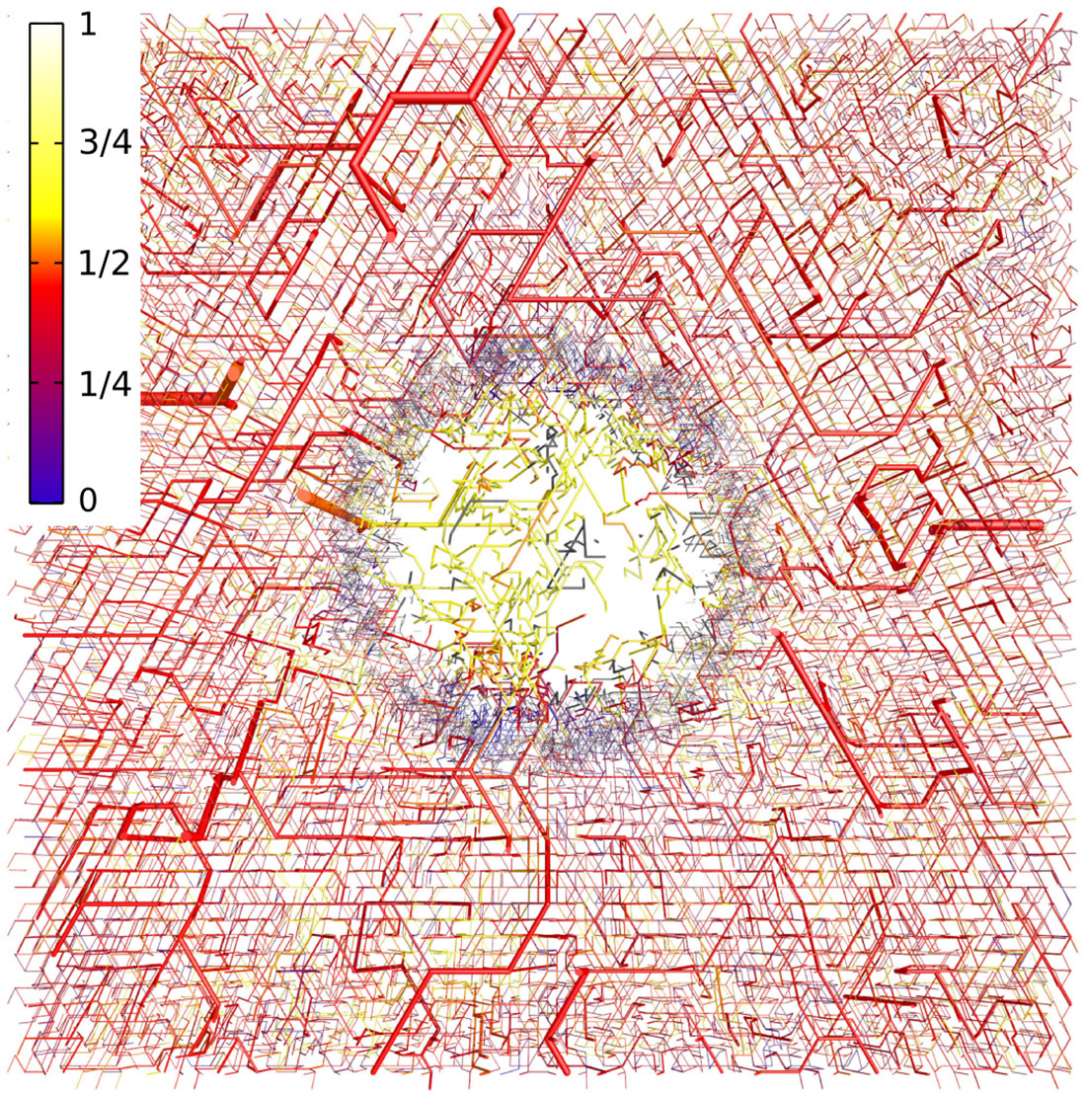
Hematocrit distribution corresponding to Fig 5D, showing the same slice of the vessel network, but color coded for *H*. Our blood flow model includes the phase separation effect which leads to a severe reduction of the hematocrit in the peripheral tumor network and an elevated hematocrit in the vessels at the tumor center.

Tumor vessels in the central region of the tumor vary significantly in their oxygen content. Some vessels carry highly saturated blood, in particular when connected to nearby arterioles. When isolated vessels thread the tumor over distances of several hundred micrometers, blood oxygen saturation *S* visibly decreases to the point where vessels are completely depleted of oxygen. The tissue PO2 near such vessels follows this trend. Remarkably, the (neo-)vascular plexus around the tumor rim is dominated by severe oxygen deprivation. However, the few high caliber vessels which advance towards the tumor center carry normal amounts of oxygen. We explain this by the phase separation effect taken into account in our simulations. This means that red blood cells (RBCs) at bifurcations prefer to flow into the more strongly perfused branch [71]. Accordingly, simulated hematocrit distributions exhibit high hematocrit in central tumor vessels, as exemplarily shown in Fig 6. We conclude that RBCs are redirected into central vessels, consequently leading to a lack of RBCs in the peripheral capillary plexus and a drastically decreased oxygen carrying capacity. The impaired oxygen carrying capacity, in turn, results in fast oxygen depletion to hypoxic levels which could drive further invasive behavior in real tumors. On average, the hematocrit taken over the concentric core region 100 *μm* beyond the invasive edge is elevated to 117% of baseline hematocrit in normal tissue. In contrast, the average taken over annular shells near the invasive edge of 50 *μm* thickness drops to 66%.

For some applications, such as models of tumor growth, it might be sufficient to implement a simplified model of tissue oxygenation, setting intravascular PO2 constant, thus treating it as input parameter. Therefore, in S1 Fig we briefly consider such a simplification and highlight qualitative differences to results obtained by our full model of oxygenation.

### Model validation: vascular morphology, blood flow and oxygenation

In order to validate our simulation model we calculated various biophysical quantities and compared the results with data available in the literature. Values listed in Tables 3 and 4 represent averages (means) over the results of all 90 vascular networks, including standard deviations, at a given time. The initial networks (*t* = 0) are taken to represent vasculature in normal breast tissue, neglecting the influence of the small tumor of radius 250 *μm* at the center of the simulation domain. Data given in column “Initial” are therefore associated with normal breast tissue. Data for the cases BASE, CMPR and METAB were calculated at the end (*t* = 600 *h*) of the tumor growth process.

**Table 3:** Morphological data: Simulation results and literature values.

Symbol	Unit	Initial	Clin./Lit.	BASE/METAB	CMPR	Clin./Lit.
*MVD*, *L*_*D*_	*mm*^−2^	111 ± 0.4	115 ± 8 [84]	112 ± 19	133 ± 18	88 (15 − 348) [85]
						20 (0 − 157) [86]
						21 (9 − 101) [7] (a)
*rBV*	%	0.98 ± 0.10	0.8 ± 0.3 [22] (b)	1.9 ± 0.6	1.8 ± 0.9	2.4 ± 1.5 [22] (b)
			3.4 ± 0.6 [87] (c)			4.3 ± 1.3 [87] (c)
						0.67(0.2 − 3.4) [7] (a)
*r*	*μm*	4.1 ± 0.1	-	5.9 ± 0.4	5.2 ± 1	9.23 ± 0.27 [7] (a)
*S*_*D*_	*mm*^−1^	2.8 ± 0.04	-	4.2 ± 1	4.4 ± 1.1	0.5 − 5.4 [7] (a)
*S*_*D*_/*rBV*	*μm*^−1^	0.29 ± 0.03	-	0.22 ± 0.03	0.27 ± 0.06	0.165 [7] (a)
*rBF*	*mlg*^−1^ *min*^−1^	0.053 ± 0.014	0.06 [8]	1.6 ± 1.3	1.2 ± 1.3	0.32 [8]
			0.056 ± 0.014 [88]			0.19 ± 0.1 [87]
			0.04 ± 0.01 [87]			0.29 ± 0.17 [88]
			0.028 ± 0.017 [89]			0.07 − 0.16 [90] (d)
						0.001 − 2 [7] (a)
*rBF*_*scaled*_	*mlg*^−1^ *min*^−1^	-	-	0.12 ± 0.04	0.091 ± 0.050	-

Shows averages and standard deviations taken over 90 initial networks (*t* = 0 *h*) and tumors (*t* = 600 *h*) for cases BASE, CMPR and METAB. Morphological data for BASE is the same as METAB. We compiled clinical data, data from animal models and theoretical data taken from literature in the columns “Clin./Lit.” for normal (left) and tumor tissue (last col.), respectively. Microvascular density obtained by our model and from [7] is given as the line density *L*_*D*_ whereas data from [85, 86] are the medians of histological *MVD*. *rBF*_*scaled*_ is the initial tissue *rBF* scaled by the ratio of perfusions, *rBF*, of the iso-volumetric tumor sphere at *t* = 0 and at *t* = 600 *h* (see text).

(a) Obtained from digitized animal model tumor network obtained by *μ*-CT.

(b) *rBV* estimated from *c*_*Hb*_ assuming 2.18 *μmol*/*l* hemoglobin in blood.

(c) Data from [87] was obtained by PET of human breasts. Note that PET data from [87] for tumor tissues are considered unreliable (see text).

(d) obtained from human breast cancer xenografts in animal model.

**Table 4:** Oxygenation data: Simulation results and literature values.

Symbol	Unit	Initial	Clin/Lit.	BASE	CMPR	METAB	Clin/Lit.
〈*S*_*in*_〉_*q*_		-	0.98 [91] (e)	-	-	-	-
*rJ*_*in*_	*μlO*_2_ *ml*^−1^ *min*^−1^	12 ± 3	-	331 ± 266	249 ± 282	331 ± 265	11 − 27 [90] (d)
*rJ*_*in*,*scaled*_	*μlO*_2_ *ml*^−1^ *min*^−1^	-	-	25 ± 10	18 ± 12	25 ± 10	-
*j*_*tv*_	*μlO*_2_ *min*^−1^ *cm*^−2^	0.12 ± 0.001	0.6 [92] (f)	0.24 ± 0.02	0.20 ± 0.02	0.24 ± 0.06	-
*MRO*_2_	*μlO*_2_ *ml*^−1^ *min*^−1^	3.3 ± 0.1	4.5 ± 1 [87]	12 ± 1	12 ± 2	13 ± 3	6.6 ± 2.8 [87] (c)
			33 ± 5 [93] (i)				40.6 ± 7.8 [77] (g)
							21 − 52 [83] (g)
							33.4 ± 8.3 [83] (h)
							4.5 − 11.8 [90] (d)
*OEF*		0.34 ± 0.10	0.65 ± 0.1 [87]	0.068 ± 0.050	0.11 ± 0.09	0.068 ± 0.051	0.23 ± 0.08 [87]
			0.44 ± 0.06 [93] (i)				0.3 − 0.52 [90] (d)
*OEF*_*scaled*_		-	-	0.55 ± 0.19	0.88 ± 0.46	0.55 ± 0.22	-
*c*_*Hb*_	*μmol*/*l*	24 ± 3	17.3 ± 6.2 [22]	47 ± 14	43 ± 19	47 ± 14	53 ± 32 [22]
			16 ± 4 [23]				70 ± 35 [23]
*Y*		0.70 ± 0.10	0.74 ± 0.14 [22]	0.81 ± 0.10	0.73 ± 0.14	0.81 ± 0.10	0.72 ± 0.14 [22]
			0.74 ± 0.09 [23]				0.71 ± 0.10 [23]
*P*	*mmHg*	39 ± 6	-	45 ± 11	34 ± 13	45 ± 12	-
*P*_*t*_	*mmHg*	36 ± 6	30 [94] (k)	35 ± 14	29 ± 14	35 ± 14	30 (0 − 100) [14] (j)
			65 (12 − 100) [14] (j)				
			40 − 50 [95] (k)				

This is a continuation of Table 3 with data of tumor and normal tissue oxygenation. 〈*S*_*in*_〉_*q*_ denotes the flow-weighted blood oxygen saturation at input. *rJ*_*in*_ denotes the oxygen supply at input *rJ*_*in*_ = *c*_0_ *H*〈*S*_*in*_〉_*q*_ ⋅ *rBF*. For tumors, *rJ*_*in*,*scaled*_ denotes the scaled quantity where *rBF*_*scaled*_ is used. In addition to the following notes, see Table 3 for further notes.

(e) Oxygen saturation of human arterial blood at sea level.

(f) Obtained by oxygen micro-electrode measurement in rat brains [92].

(g) Obtained from various human cancer cell spheroids, excluding breast cancer.

(h) Obtained from MDA-MB-468 human breast cell spheroids.

(i) Obtained from human brain by PET.

(j) Median and range; obtained from human breast cancer and normal tissue using polarographic needles.

(k) Obtained from peritumoral skin tissue [94] and muscle tissue in breast tumor-bearing rats [95].

Explicit formulas for the parameters listed are given in S2 Appendix. It should be kept in mind that for most biophysical variables, results corresponding to a particular vascular network represent (weighted) averages taken over all vessel segments of the network (s. S2 Appendix). For comparison, Tables 3 and 4 also include clinical data, data obtained from animal models and theoretical results taken from the literature. In the following we comment on some of the simulated and literature data. Additional details are provided in S4 Appendix.

In general, good to fair agreement was achieved between simulation results and corresponding literature data. In particular total hemoglobin concentration and tissue blood oxygenation *Y*, known from optical mammography were reproduced both for normal breast tissue and breast tumors. Likewise, simulated vascular volume densities fall within the range of *rBV* values reported in the literature. In Table 3 we take simulated length density *L*_*D*_, i.e. the total length of the vascular network divided by the tissue volume (s. S2 Appendix), as measure for microvessel density *MVD*, conventionally obtained by microscopically counting the number of microvessels per area of a tissue section. Simulated length density *L*_*D*_ and clinical *MVD* of normal breast tissue agree quantitatively and length densities *L*_*D*_ of the tumor model variants fall within the range of *MVD* reported for breast cancers in the literature. Average vessel radii corresponding to the tumor model variants BASE, CMPR and METAB are about 60% of those reported by Stamatelos, probably because the maximal dilatation radius was limited to 10 *μm* (BASE/METAB) and 14 *μm* (s. Table 2). Tumor vessel surface density *S*_*D*_ and tumor vessel surface density over tumor vascular volume density *S*_*D*_/*rBV* are consistent with the data reported by Stamatelos et al. [7].

Regional blood flow, *rBF*, i.e. perfusion, is given by the ratio of total blood flow entering a selected tissue region Ω to its volume |Ω| (s. S2 Appendix). To calculate host perfusion *rBF*_*norm*_ of the entire simulation cuboid we sum the flow rates *q* over all arterial root nodes and divide by the volume of the simulation box |Ω| = *L*^3^. Good agreement between simulated perfusion *rBF*_*norm*_ and clinical data was achieved. However, for a partial volume, such as a small spherical tumor inside the tissue cuboid, some caution must be taken. Assuming a constant surface flux density of blood, the perfusion of the small spherical volume scales as 1/*R*_*tum*_. Indeed, perfusion rates *rBF*_*tum*_ predicted for the relatively small tumors, simulated here, are much larger than clinical *rBF* data from breast tumors which are typically of several *cm*^3^ in volume. To estimate the perfusion *rBF*_*scaled*_ that corresponds to larger simulated tumors, we scale *rBF*_*tum*_ in the following way: *rBF*_*scaled*_ = *rBF*_*norm*_ ⋅ *rBF*_*tum*_/*rBF*_*sph*,*norm*_. Here, *rBF*_*sph*,*norm*_ denotes the perfusion of the isovolumetric spherical volume *V*_*sph*_ of normal tissue (*t* = 0) that corresponds to the tumor spheroid at *t* = 600 *h*. The perfusion rates *rBF*_*tum*_ and *rBF*_*sph*,*norm*_ are obtained from summation of blood flow *q* over those vessels that penetrate the surface of the sphere *V*_*sph*_ and have a blood flow direction into the spherical tissue volume. Scaled perfusion *rBF*_*scaled*_ is lower by a factor 2—3 compared to clinical data on breast cancers. However, scaled perfusion falls within the range of perfusion values reported by Stamatelos [7] and Kallinowski et al [82] for human breast cancer animal models. In addition, Table 3 lists the regional blood flow of the small spherical tumor *rBF*_*tum*_ proper.

The metabolic rate of oxygen consumption *MRO*_2_ of normal tissue is mainly determined by the assumed Michaelis-Menten maximal rate *M*_0_ (s. Table 1), since (average) partial oxygen pressure *P*_*t*_ in tissue is considerably larger than the Michaelis-Menten oxygen half pressures. As a result, the simulated *MRO*_2_ for normal breast tissue favorably compares with clinical data. To our knowledge there are no reliable clinical data available on metabolic rates of oxygen consumption of breast cancers. Therefore, we considered a maximal consumption rate *M*_0_(*tumor*) for cases BASE and CMPR, consistent with the metabolic rates of oxygen consumption reported for xeno-transplanted human breast cancers in animal models [82] (see above for case METAB). More recently, metabolic rates of oxygen consumption, ranging from 21 − 52 *μlO*_2_/*g*/*min*, were determined from multicellular tumor spheroids of various human cancer cell lines, including breast cancer (33 ± 8 *μlO*_2_/*g*/*min*), serving as avascular in-vitro tumor models [77, 83], also consistent with our choice of *M*_0_.

The cohort average of the predicted tumor O_2_ consumption *M*(*P*_*t*_) proper is close to the assumed maximal rate *M*_0_, indicating that predominantly *P*_*t*_ > *P*_*M*50_, in spite of severely decreased PO2 levels. We explain this result by the assumed value of *P*_*M*50_ = 2 *mmHg* which is quite small. Furthermore, average partial oxygen pressures in normal breast tissue and breast tumors are consistent with clinical data.

We conclude that our model is in general capable to correctly account for the biophysical and physiological quantities simulated taking into account their considerable standard deviations and the considerable variations of clinical, animal and theoretical data available in literature.

### Variance of tissue hemoglobin concentration, tissue blood oxygen saturation and perfusion due to individual initial vascular configurations

**Fig 7:**
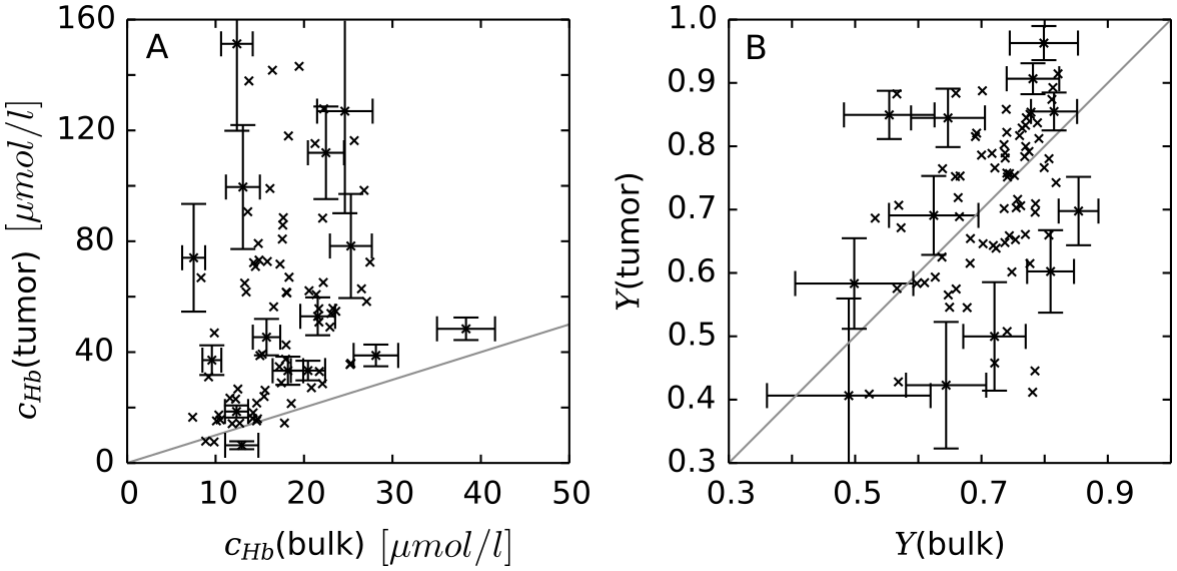
Physiological parameters of tumors versus those of corresponding healthy breast tissue for 87 patients. (A, left) total hemoglobin concentration *c*_*Hb*_; (B, right) blood oxygen saturation *Y*. Reproduced from data shown in Ref. ([22], Fig 3A, B).

**Fig 8:**
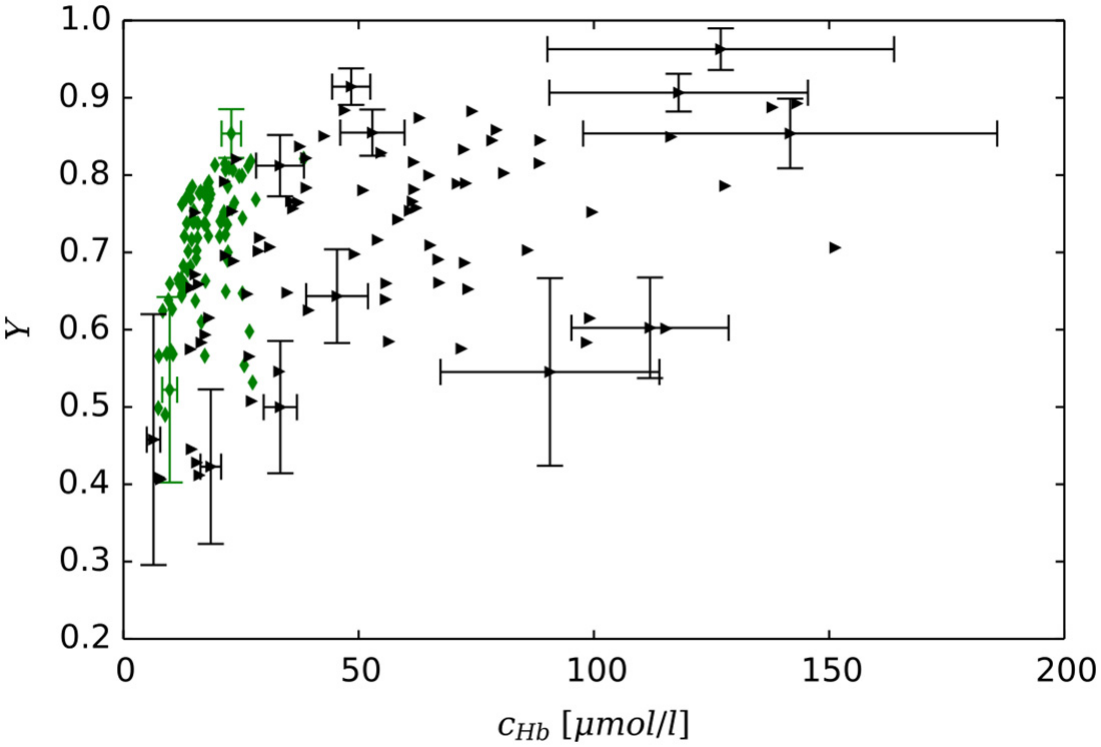
Blood oxygen saturation versus total hemoglobin concentration. Error bars ([22], cf. Fig 3a,b) on tumor data correspond to uncertainties of tumor radius *a*_*T*_ and location *z*_*T*_ along compression direction, besides statistical contributions, error bars on healthy breast tissue data reflect statistical uncertainties only. Reproduced from data shown in Ref. ([22], Fig 5).

It is the focus of the present paper to simulate tissue total hemoglobin concentrations *c*_*Hb*_ and tissue blood oxygen saturation *Y* of tumors and host tissue and compare the results with the clinical data on breast tumors and surrounding normal breast tissue obtained from a cohort of 87 breast cancer patients using optical mammography, reported by Grosenick et al. ([22], cf. Figs 3A, B, Fig 5]). Since the present paper frequently refers to the clinical results, we reproduce the relevant figures, see Figs 7 and 8.

**Fig 9:**
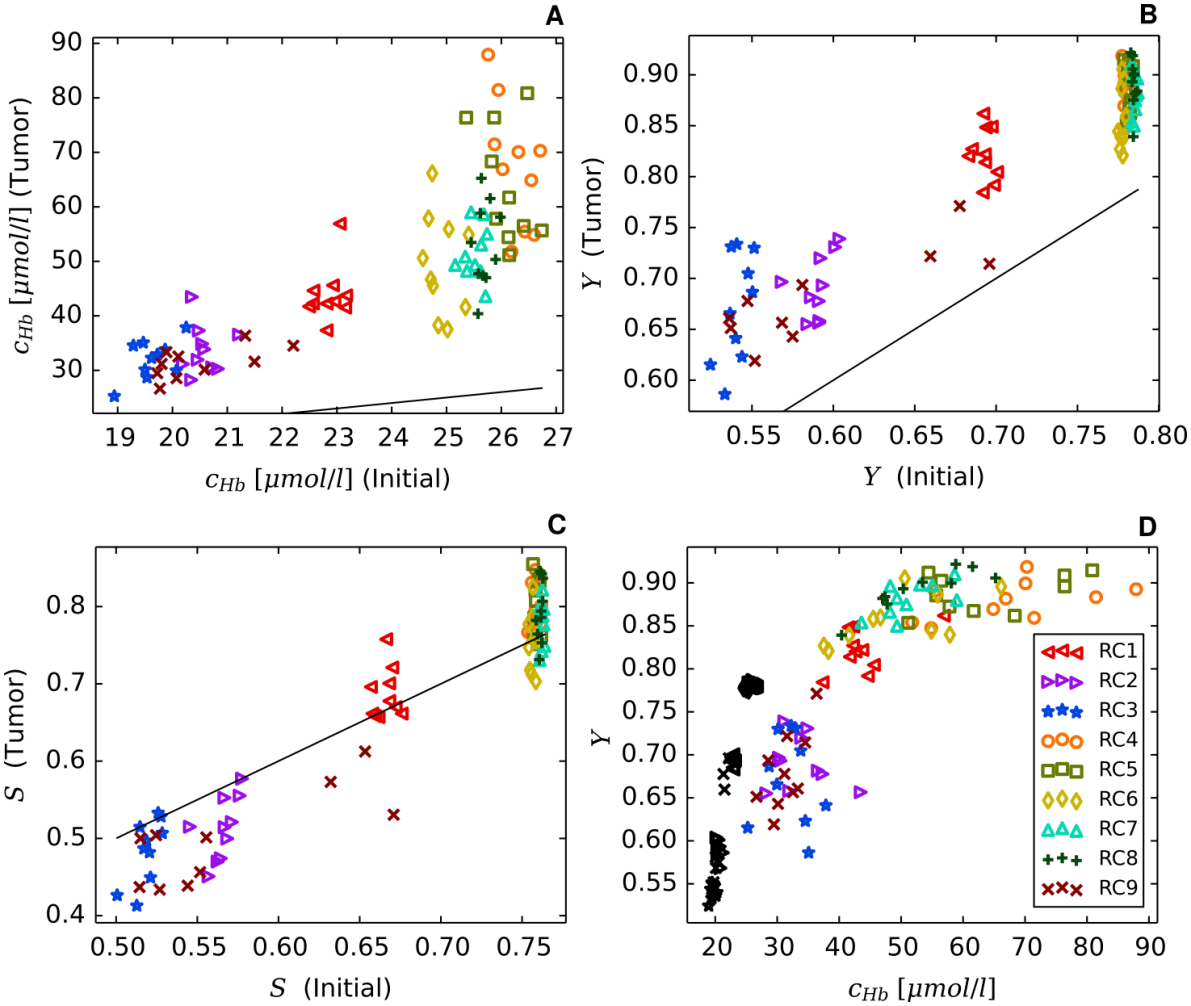
Tissue hemoglobin concentrations, volume-weighted tissue blood oxygen saturations *Y* and length-weighted blood oxygenation *S* (Case BASE). Each data point corresponds to a different simulation run, where the color code indicates the corresponding root node configuration (cf. Fig 3, see text). Panel A displays hemoglobin concentration *c*_*Hb*_ of tumor tissue versus *c*_*Hb*_ in normal tissue. Panel A also corresponds to case METAB, differing from case BASE in tissue oxygen metabolism only. Panel B correlates RBC- volume-weighted tissue blood oxygen saturation *Y*, Panel C length-weighted blood oxygenation *S* in tumorous and normal breast tissue. Panel D shows a plot of *Y* against tissue hemoglobin concentration *c*_*Hb*_ where tumor (colored symbols) and normal tissue (black symbols) are both shown as different point sets. Black lines in Panel A, B and C represent diagonals separating tumor data above from below normal.

**Fig 10:**
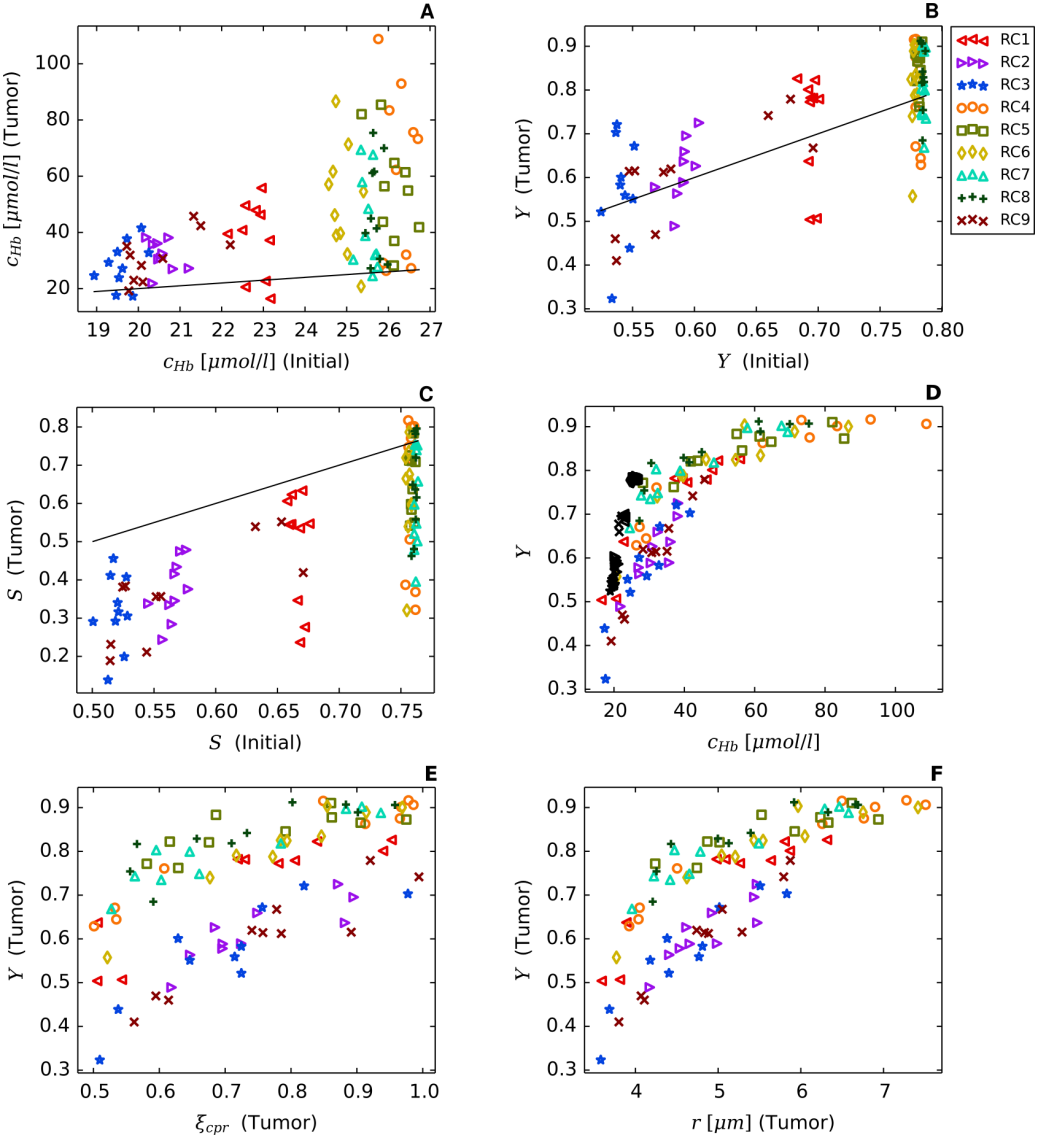
Tissue hemoglobin concentrations, volume-weighted tissue blood oxygen saturations *Y* and length-weighted blood oxygenation *S* (Case CMPR). This figure is the analog of Fig 9, but for case CMPR. Each of the 90 simulation runs was carried out with a different randomly selected factor *ξ*_*cpr*_ for reducing all tumor vessel radii (s. Eq (40)) simulating solid stress. Fig 10 Panel A-C correlate tissue hemoglobin concentration *c*_*Hb*_, RBC-volume-weighted blood oxygen saturation *Y* and length-weighted blood oxygen saturation *S* of tumor (*t* = 600 *h*) and normal (*t* = 0 *h*) tissue, respectively. In Fig 10D tissue blood oxygenation *Y* is plotted versus tissue hemoglobin concentration *c*_*Hb*_ separately for tumors (colored) and normal tissue (black). Fig 10E and 10F correlate tissue blood oxygen saturation *Y* of tumors with factor *ξ*_*cpr*_ and average vessel radius *r*. Generally, *Y*_*tum*_ decreases with decreasing average *r* and decreasing *ξ*_*cpr*_, corresponding to higher compression. Black lines in (A), (B) and (C) represent diagonals separating tumor data above from below normal. The color code to identify root node geometry is the same as in Fig 9.

**Fig 11:**
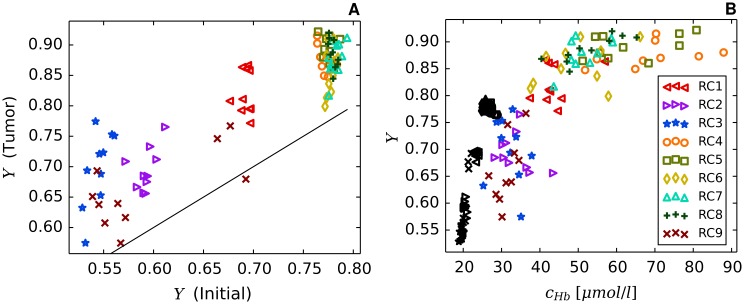
Volume-weighted tissue blood oxygen saturations *Y* and hemoglobin concentrations (Case METAB). This figure is the analog of Fig 9 for case METAB, using the same 90 vascular network realizations at *t* = 0 and *t* = 600 *h* as in case BASE. For each network realization at the end of the growth process, oxygen related parameters were simulated by randomly selecting *M*_0_ from a lognormal distribution (see text). Fig 11A correlates tissue blood oxygen saturation in tumorous and normal tissue. In Fig 11B, tissue blood oxygenation is plotted versus tissue hemoglobin concentration for tumors (colored symbols) and normal tissue (black symbols) separately. The black lines in (A) represents the diagonal separating tumor data above from below normal. The color code to identify root node geometry is the same as in Fig 9.

**Fig 12:**
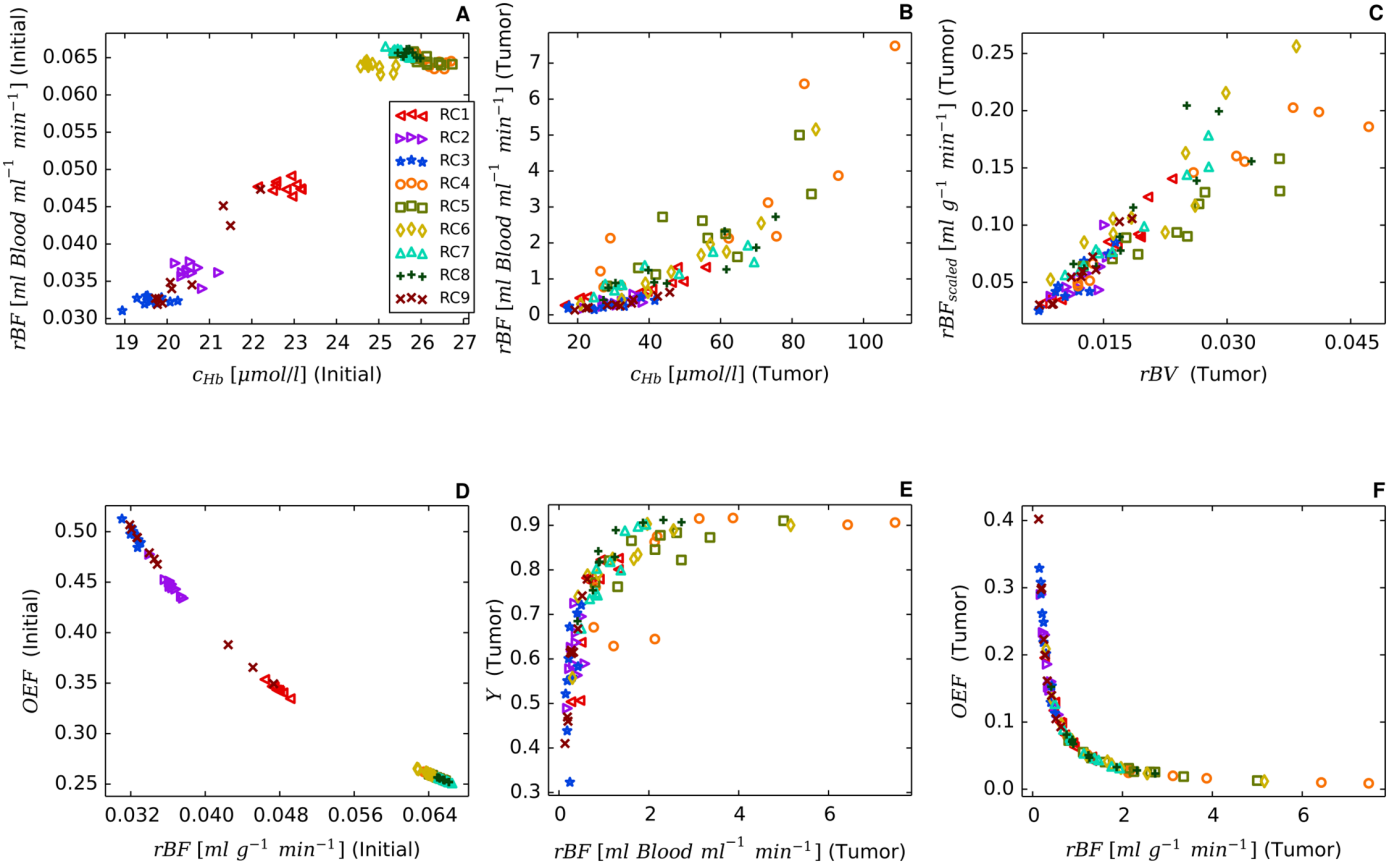
Correlation of other dynamic vascular and metabolic quantities (Case CMPR). Fig 12A and 12B relate perfusion to tissue hemoglobin concentration for normal tissue (t = 0) and tumors (t = 600 h), respectively, whereas Fig 12C correlates tumor scaled perfusion *rBF*_*scaled*_ (see text) with vascular volume density *rBV*. Fig 12D and 12F correlate oxygen extraction fraction *OEF* with perfusion *rBF* for normal (*t* = 0) and tumorous (*t* = 600 *h*) tissue, respectively. Fig 12E displays tissue blood oxygen saturation *Y* versus perfusion (*t* = 600 *h*). A comparison of Fig 12E and 12F shows implicitly the negative correlation of *OEF* and *Y* for normal tissue. Each data point corresponds to one of the 90 simulation runs, where the color code to identify the root node geometry is the same as in Fig 9.

**Fig 13:**
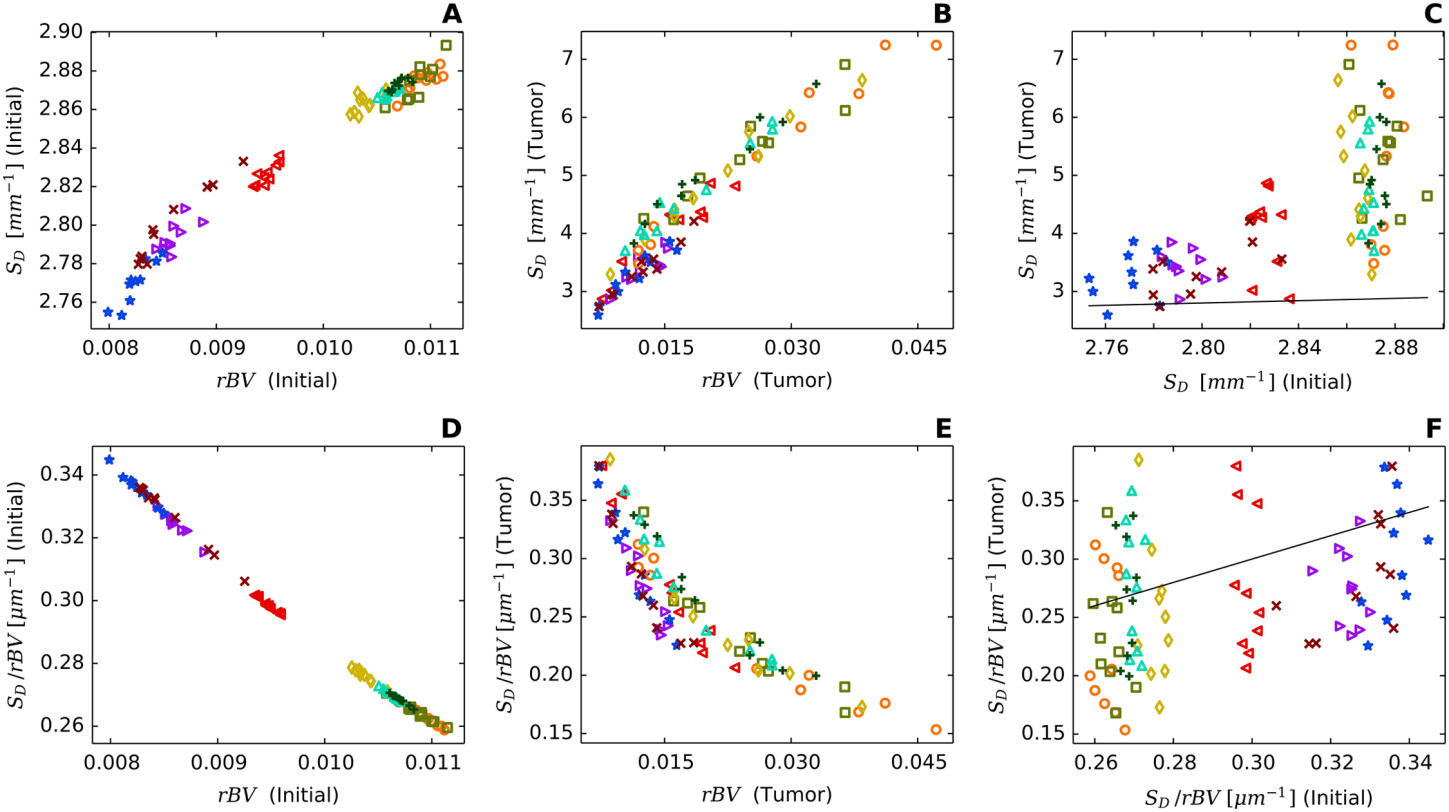
Correlation of geometric vascular quantities (Case CMPR). Fig 13A and 13B relate vessel surface with vessel volume densities for normal (*t* = 0) and tumorous (*t* = 600 *h*) tissue, respectively, whereas Fig 13C compares vessel surface densities of tumors and normal tissue. Correlations of surface to volume ratios *S*_*D*_/*rBV* with vessel volume density *rBV* are shown in Fig 13D and 13E for normal tissue (*t* = 0) and tumors (*t* = 600 *h*), respectively. Fig 13F relates vessel surface to vessel volume densities for tumorous(*t* = 600 *h*) and normal (*t* = 0) tissue. Each data point corresponds to one of the 90 simulation runs, where the color code to identify the root node geometry is the same as in Fig 9. Black lines in (C), (F) represent diagonals separating tumor data above from below normal.

In Figs 9, 10 and 11 corresponding to the model variants BASE, CMPR and METAB, respectively, we likewise correlate simulated tissue hemoglobin concentration and tissue blood oxygenation in normal and tumorous tissue separately for each of the 90 network realizations at the beginning (*t* = 0) and at the end (*t* = 600*h*) of the tumor growth process, thus mimicking a patient cohort. Figs 9–13 illustrate that variations of normal vascular configurations in the cohort result in considerable data scatter of simulated parameters, explaining the observed scatter in clinical data on the patient cohort. Additionally the root node configuration (cf. Fig 3), associated with a particular data point, is indicated by a colored symbol.

Depending on root node configuration, drastic variations in microvascular density *MVD* at the tumor center, vascular volume density *rBV* and tissue hemoglobin concentration *c*_*Hb*_ as well as perfusion and tissue blood oxygenation *Y* in tumors are observed. The same parameters are subject to fluctuations over the initial networks, yet in many cases to a much lesser relative magnitude. Our main finding, that vascular compression in solid tumors is responsible for low tissue oxygen saturation is supported by the sections below.

### Perfusion and tissue hemoglobin concentration are positively correlated

Fig 12A and 12B show that perfusion rates *rBF* correlate positively with tissue hemoglobin concentration *c*_*Hb*_ in both normal tissue and tumors. According to Fig 12A, perfusion of the initial vasculature (normal tissue) is almost linearly dependent on *c*_*Hb*_. Since we take *c*_*Hb*_ to be proportional to *rBV*, perfusion *rBF* and vascular volume density *rBV* are likewise well correlated, illustrated for scaled perfusion *rBF*_*scaled*_ in Fig 12C, exhibiting a linear dependence approximately. Since high caliber vessels crisscrossing the tumor core provide dominant contributions to *c*_*Hb*_ and *rBV*, and, at the same time, carry high blood flow due to low hydrodynamic resistance, such correlation is to be expected. Details of this correlation are determined not only by vascular morphology, e.g. the initial vessel network growth model, but also by boundary conditions at inlets and outlets, i.e. the assumed blood pressure-radius relation (s. Eq (3) in S1 Appendix).

To understand the relation between number of root nodes, vascular volume density *rBV* and radii of root nodes in initial networks (*t* = 0, normal tissue), we consider a simplified system in S3 Appendix. There, we calculate *rBV* assuming perfect binary vascular trees depending on the number of (identical) trees (i.e. number of root nodes), yet keeping the total number of vessel segments constant. Vessel radii, in our model of the initial vasculature, are governed by Murray’s law. Thus, the radius of mother vessels grows by a factor of 2^1/3^ compared to their daughter vessels. Geometries with fewer root nodes (e.g. RC4 through RC8, s. Fig 3) correspond to more extended arterio-venous trees compared to configurations (e.g. RC1, RC2, and RC3) with many arterial and venous roots. It follows that *rBV* and root node radii are larger for geometries with fewer root nodes. Furthermore, according to the assumed blood pressure-radius relation, larger root node radii correspond to larger arterial-venous pressure differentials, increasing perfusion. Conversely, configurations with larger numbers of root nodes are associated with lower *rBV*, lower root vessel radii, and hence lower arterio-venous blood pressure differentials.

Thus, vascular network structure and pressure-radius relation of the feeding and draining vessels determine vascular volume and perfusion. In a cohort of real tissue sections we expect a similar multitude of vascular configurations with the therewith associated data scatter.

### Vascular dilatation leads to elevated tumor oxygen saturation

The *c*_*Hb*_(tumor)-*c*_*Hb*_(normal) correlations for cases BASE and METAB (Fig 9A) and for case CMPR (Fig 10A) qualitatively agree with the clinically observed correlation between tissue hemoglobin concentrations in breast tumors and surrounding normal breast tissue (Fig 7a, ([22], Fig 3a)). Since total hemoglobin concentration is approximately proportional to vascular volume density *rBV*, the higher hemoglobin concentration reflects the larger *rBV* of tumors, explained by neo-angiogenesis and vessel dilatation, outweighing vascular regression. For case CMPR we raised the maximum dilatation radius *r*^(*max*)^ from 10 *μm* (BASE, METAB) to 14 *μm* (s. Table 2) in order to compensate for the decrease of vascular volume density *rBV* associated with an overall compression of tumor vessels. Consequently, the ensemble averages of *rBV*(BASE, METAB) and *rBV*(CMPR) are approximately the same (s. Table 3). Likewise, the correlation plots *c*_*Hb*_(*tumor*)-*c*_*Hb*_(*normal*) shown in Figs 9A and 10A agree qualitatively, although quantitative differences exist comparing individual representations.

For clarity we want to point out the difference between tissue blood oxygen saturation *Y* = *c*_*HbO*_/*c*_*Hb*_ and average vascular oxygen saturation 〈*S*〉. The latter quantity is the length-weighted average of blood oxygen saturation taken over all vessel segments of the vascular network (s. S2 Appendix). High caliber tumor vessels contribute to 〈*S*〉 according to their—likely higher—blood oxygen saturation, but contribute to *Y* additionally by their large relative RBC volume. A plot of 〈*S*〉(tumor) versus 〈*S*〉(normal) shows 〈*S*〉(tumor) to fall above and below normal for case BASE (Fig 9C), whereas 〈*S*〉(tumor) is generally lower than normal when M-M oxygen consumption rate is raised to *M*_0_(*tumor*) = 24.6 *μlO*_2_/*ml*/*min* (s. Panel D of Fig A in S2 Fig) and in the compression case CMPR (Fig 10C).

Figs 9D, 10D and 11B, being qualitatively similar, illustrate the correlation between tissue blood oxygen saturation *Y* and tissue total hemoglobin concentration *c*_*Hb*_ for cases BASE, CMPR, METAB, respectively. Analogous to Fig 8, separate correlations are shown for normal and tumorous tissue. At an arbitrarily selected hemoglobin concentration *c*_*Hb*_ there exists a lower bound on *Y*, clearly discernible in Figs 9D, 10D and 11B despite considerable data scatter. For tumors, tissue blood oxygen saturation *Y* tends towards an upper limit at high tissue total hemoglobin concentration.

We explain this asymptotic behavior of *Y* by first recognizing that vascular volume—essentially *c*_*Hb*_—and perfusion *rBF* are positively correlated as shown in Fig 12A and 12B. An increase in vascular volume allows for stronger perfusion at given blood pressure boundary conditions. By definition, tissue blood oxygen saturation *Y* is negatively correlated with oxygen extraction fraction *OEF*. Moreover, the amount of oxygen extracted from the blood stream is bound from above by the rate of radial diffusion and the local metabolic oxygen demand of tissue. An increase of vascular oxygen influx *q* ⋅ *c* beyond what can be extracted, leads to luxurious perfusion. In this imbalanced state, most of the oxygen exits the system again through draining vessels. In this case the oxygen extraction fraction *OEF* is relatively low. This means, with increasing perfusion *Y* approaches asymptotically the blood oxygen saturation level at the input.

Luxurious perfusion is evidently predicted in tumors exhibiting high tissue hemoglobin concentrations *c*_*Hb*_. We conclude that in such tumors particularly few and thick vessels criss-cross the central tumor volume. Such vessels are associated with relative large blood flow rates *q*, relatively high blood oxygen saturation *S*, besides enhanced hematocrit (s. Figs 5D and 6). Since tissue blood oxygen saturation *Y* is the RBC-volume-weighted average of blood oxygen saturation *S* taken over all vessel segments (s. Eq (39)), high caliber vessels predominantly contribute to *Y*.

Paradoxically, hypoxia is prevalent in many tumors, including our simulations. However, a physiologically normal oxygen supply requires a homogeneous distribution of a capillary bed exhibiting a physiological surface area per vascular volume. Instead, tumor blood vessels are very heterogeneously distributed.

The resulting simulated tissue blood oxygen saturation *Y* of tumors falls above normal (case BASE, METAB), although tumor oxygen demand was raised, i.e. the Michaelis-Menten metabolic rate of oxygen consumption *M*_0_ in tumorous tissue was assumed to be four times higher compared to normal tissue (s. Table 1).

Figs 9D, 10D and 11B are in qualitative agreement with clinical data (s. Fig 8), apart from missing simulated data above the lower bounds of *Y*. This discrepancy might be explained by partial volume effects. Implicitly our simulations assume the spherical tumor to consist of a homogeneous distribution of viable tumor cells, apart from its vasculature, thus assuming homogeneity with respect to oxygen consumption. However, breast tumors of patients may contain necrotic regions or partial volumes consisting of viable tumor cells and normal breast cells. In either case the effective oxygen demand is reduced leading to higher tissue blood oxygen saturations *Y* at constant tissue hemoglobin concentration *c*_*Hb*_.

### Tumor blood flow rates outweigh increased oxygen demand

Clinical data on metabolic rate of oxygen consumption of tumors are scarce. Our choice of *M*_0_(*tumor*) = 14.8 *μlO*_2_/*ml*/*min* corresponds to simulated metabolic rates *MRO*_2_ in tumors to be about 12 *μlO*_2_/*ml*/*min* (s. Table 4), consistent with the high end of oxygen metabolic rates reported for human xeno-transplanted breast cancers in animal tumor models [82]. Considerably higher oxygen consumption rates (*M*_0_ = 33.4 ± 8.3 *μlO*_2_/*ml*/*min*) of human breast carcinoma cells (MDA-MB-468) were recently reported based on in-vitro measurements of oxygen flux above a monolayer of the tumor cells [83]. Assuming a M-M consumption rate of *M*_0_ = 24.6 *μlO*_2_/*ml*/*min* raised the simulated tissue metabolic rate of oxygen consumption to 18.6 *μl* *O*_2_/*ml*/*min* (BASE), yet did not change the situation, i.e. tissue blood oxygen saturation *Y* of tumors was simulated to be above normal (s. Panels A and B in Fig A in S2 Fig). Likewise, simulated tissue blood oxygen saturation *Y* of tumors (case METAB) changed only little compared to case BASE (s. Figs 9B and 11A), when the metabolic rate *M*_0_ was randomly selected from a lognormal distribution for each of the 90 growth processes to simulate phenotypical differences between breast cancer patients. We therefore conclude that tissue blood oxygen saturation *Y* of breast tumors below normal (Fig 7B) is unlikely caused by high metabolic rate of oxygen consumption in tumorous tissue, rather than by insufficient oxygen supply.

### Vascular compression is responsible for low tumor oxygen saturation

It follows that the growth processes of tumor vasculature of case BASE (METAB) need to be augmented (case CMPR). Our method to simulate tumor vasculature had been developed previously based on morphologic data (*MVD*) of melanomas. It is therefore not surprising that addition of data on tissue blood oxygen saturation *Y* depending on metabolic characteristics of tumor tissue besides vascular structure and hemodynamics, requires modifications of previously employed models. Tissue blood oxygen saturation *Y* is (negatively) correlated with blood oxygen extraction fraction *OEF* = *MRO*_2_/*rJ*_*in*_, where *rJ*_*in*_ is the oxygen influx per unit volume of tissue. In simulations, tumor oxygen extraction fraction can be increased and hence tissue blood oxygen saturation *Y* lowered by compressing vessels, thus restricting oxygen influx, while keeping tissue metabolic rate of oxygen consumption *MRO*_2_ (approximately) constant. Therefore, we tentatively assume that blood vessels are compressed during tumor growth by solid pressure, known to be elevated in solid tumors [9, 11], caused by proliferating tumor cells and modifications of the tumor extracellular matrix, e.g. by increased deposition of hyaluronan [96]. Previously, compression of intratumoral vessels was observed in an animal model, attributed to proliferating tumor cells [10]. Presently, the precise mechanisms are still under debate by which extravascular pressure compresses tumor vessels [78, 79].

For simplicity, CMPR assumes the same radius reduction factor *ξ*_*cpr*_ for all vessel segments, where *ξ*_*cpr*_ is randomly selected for each of the 90 tumor growth processes. Case CMPR shows tumor blood oxygen saturation *Y* to fall above and below normal (Fig 10B), consistent with the clinical data. Qualitatively speaking, for a sufficiently small radius reduction factor *ξ*_*cpr*_ selected, vessel segments whose radii fell above the maximal dilatation radius *r*^(*max*)^ = 14 *μm* (CMPR) at the start of the tumor growth process will most likely end up to be narrower compared to case BASE, whereas radii of smaller vessel segments (*r* < *r*^(*max*)^) will be comparable for both scenarios at the end of the growth process, since the higher maximal dilatation radius of case CMPR compensates for vessel segment compression. It follows that oxygen supply by high caliber vessels threading the tumor center (s. Figs 5D and 6) is reduced, causing *Y* to fall below case BASE. On the other hand for a radius reduction factor *ξ*_*cpr*_ close to 1, tissue blood saturation *Y* of high caliber vessel segments will be comparable for cases BASE and CMPR, explaining the difference between Figs 9B and 10B. Likewise, compared to case BASE/METAB, compression of vessels with radii larger than *r*^(*max*)^ contribute to the reduction of the ensemble averages of perfusion *rBF* and scaled perfusion *rBF*_*scaled*_ by 30% and 35%, respectively (case CMPR, s. Table 3).

Fig 10E and 10F illustrate that *Y*_*tum*_ generally decreases with decreasing factor *ξ*_*cpr*_, simulating increasing vascular compression by solid stress and with decreasing average vascular radius *r*. Although we cannot rule out other biological processes that may cause radial extent of tumor blood vessels to shrink we take clinical data with *Y*_*tum*_/*Y*_*norm*_ < 1 to be indicative of vascular compression. Based on data from optical mammography [22] one might expect that tumors with *Y*_*tum*_ < *Y*_*norm*_ have vascular networks with morphological characteristics that are very different from tumors with *Y*_*tum*_ > *Y*_*norm*_. However, our results suggest that similar vascular network morphologies and similar compartmentalization cover a wide range of values for *Y*_*tum*_ above and below *Y*_*norm*_, in good agreement with clinical data. Since compression of blood vessels will impede chemotherapy we conclude that tumors with oxy-to total hemoglobin concentration below normal are less likely to respond to chemotherapy. Such behavior was recently reported for neo-adjuvant chemotherapy of locally advanced breast tumors [47].

### Oxygen extraction and vascular surface density

Fig 12D and 12F correlate oxygen extraction fraction *OEF* with perfusion *rBF* for normal and tumorous tissue, respectively. A comparison of Fig 12E and 12F illustrates implicitly the negative correlation of tissue blood oxygenation *Y* and oxygen extraction fraction *OEF* for tumorous tissue (CMPR). Such a correlation is also seen for normal tissue. As a matter of fact, for normal tissue, the sum *OEF* + *Y* is only slightly larger than 1, being rather independent of perfusion. Since metabolic rate of oxygen consumption *MRO*_2_ is essentially an input in either case and oxygen concentration *c*_*in*_ of feeding vessels is nearly constant, Fig 12D and 12F show a hyperbolic dependence of *OEF* = *MRO*_2_/*c*_*in*_/*rBF* on perfusion *rBF*.

Fig 13 relates vascular geometric properties of normal and tumorous (case CMPR) tissue that are not easily amenable to clinical measurements. As expected, vascular surface density *S*_*D*_ increases with increasing volume density *rBV*, both for normal (Fig 13A) and tumorous (Fig 13B) tissue. Since vascular volume density is higher in tumorous tissue compared to normal tissue (s. Fig 10A), the same applies to vascular surface density (s. Fig 13C). In contrast, vascular surface to volume ratios *S*_*D*_/*rBV* decrease with increasing *rBV* in either case, probably due to the presence of larger vessels at higher fractional blood volumes (Fig 13D and 13E), yet vascular surface to volume ratios of tumors may fall above or below normal (Fig 13F), depending on whether or not the increase in tumor surface density over that of normal tissue outweighs the corresponding increase in tumor volume density over normal.

### Limitations

Although we explained salient features of the clinically observed correlations of hemoglobin concentration and tissue blood oxygen saturation between tumorous and corresponding normal breast tissue (inter-network correlations) as well as the dependence of tissue blood oxygenation on hemoglobin concentration for normal breast tissue and tumors (intra-network correlations) our model has important limitations.

Our computational method presently based on a uniform grid has limited accuracy due to poor convergence with lattice spacing *h*. Optimal convergence of the solution of elliptic partial differential equations with Dirac source terms can be achieved by adaptive meshes, concentrating discretization points near the source contributions [97]. In future, adaptive meshes should be used to improve the accuracy of our method.

The small size of the spherical tumors embedded in the simulation domain make a comparison of simulated data with clinical results uncertain, notably for perfusion. Also, the vascular structure of cm sized tumors might be different from the mm-sized tumors that were simulated. Apart from its vasculature, we implicitly assumed the growing spherical tumor to be homogeneous. However, in real tumors, viable, oxygen consuming, tumor cells are concentrated near blood vessels, whereas necrotic regions which hardly, if at all, consume O_2_ occur in regions void of vessels. This heterogeneity affects tissue PO2 distributions and therefore likely also tissue blood oxygen saturation *Y*.

The reduction of tumor vessels radii during the growth process by the same, randomly selected factor is the simplest way to account for compression by solid stress. In effect, this method decreases the radii of vessels where *r* is larger than the maximal radius *r*^(*max*)^ reached by circumferential dilatation. Furthermore, reducing *r*^(*max*)^ leads to skewed balance between microvessel density *MVD* and regional blood volume *rBV* which we currently consider as adequate. However, rather than a reduction of vessel radii, local pinch-offs of vessels are more likely to occur [12], restricting or obstructing RBC flow and thus delivery of oxygen, yet not changing significantly vascular volume density. Furthermore, only stationary solutions of perfusion were sought, although it is well known that blood flow in capillaries may be chaotic.

If possible anatomical information should be used to generate realistic geometries of feeding arteries (arterioles) and draining veins (venules), rather than arbitrarily creating geometric patterns of root nodes as in this paper. Following [98] a possible remedy to this problem might be to exclude root node geometries that result in unrealistic values of biophysical variables, e.g. unreasonably low or high perfusion rates. Furthermore, probability distributions associated with the clinical data may allow to select the most probable root node geometries.

### Conclusions

We have taken a first step to elucidate the relationship between vascular morphology, vascular oxygen concentration and oxygen delivery to tumor and host tissue, thus trying to solve an ill-posed inverse problem, namely to deduce morphological information on vascular networks from clinical coarse grained data. To this end, we related data on tissue hemoglobin concentrations and tissue blood oxygen saturations of breast cancers and surrounding host tissue to synthetic, algorithmically generated tumor blood vessel networks and networks of surrounding normal tissue, by simulating intravascular and extravascular distributions of partial oxygen pressure. Our simulations suggest that elevated fractional volume of blood vessels in breast tumors is likely the result of vascular dilatation, in spite of a drastic reduction of the number of vessels by regression and collapse. Considerable data scatter in simulated quantities, e.g. tumor tissue hemoglobin concentration and tissue blood oxygenation are related to vascular variance of initial blood vessel networks and qualitatively explain the scatter of clinical data of the patient cohort. Furthermore, breast tumors with tissue blood oxygenation above normal are likely associated with high tumor blood flow caused by high-caliber blood vessels penetrating into the tumor. Tissue blood oxygen saturation *Y* in tumors that is below normal could only be achieved by a reduction of the radii of high-caliber blood vessels, emulating compression of blood vessels caused by intra-tumoral solid stress. Factors contributing to low saturations *Y* are the reduction in blood flow, increasing the fractional oxygen extraction, and secondly, the associated reduction in vascular volume by which the blood oxygen saturation is weighted. Our simulations provide a possible explanation for tissue blood oxygenation in tumors below normal at baseline reported to be indicative of poorer prognosis for neoadjuvant chemotherapy. We expect our model to find applications to data analysis of PET, MRI, biomedical optics and hybrid techniques such as photoacoustics, probing tissue vascularization and oxygenation.

## Supporting Information

S1 Appendix Model details and validation. Details on the derivation of the transvascular oxygen mass transfer coefficient *γ* are given, simulation results are compared with literature references, estimating the accuracy of our method, and finally the blood pressure at inlets and outlets *p*^(*BC*)^(*r*) is defined.
(PDF)

S2 Appendix Explicit Formulas for Relevant Biophysical Quantities. The simulation of tumor growth and oxygen PO2 distributions in blood and tissue yields vascular flow rates *q*_*v*_, hematocrit *H*_*v*_, oxygen partial pressure *P* at discrete points on vessel axes, and tissue partial oxygen pressure *P*_*t*_. In this section explicit formulas of other derived biophysical quantities are provided.
(PDF)

S3 Appendix Regional Blood Volume of Confined Perfect Binary Trees. In this section we derive an approximative relation between the number of root nodes and the regional blood volume, provided that the total number of vessels is held constant. Here we assume that all vascular trees are perfect binary trees.
(PDF)

S4 Appendix Further details on comparison between simulated and literature biophysical data. In support of our model we further discuss simulated data listed in Tables 3 and 4 in some detail, comparing our results with clinical data, data obtained from animal models and theoretical data available in the literature.
(PDF)

S1 Fig Tissue PO2 distributions at constant vascular blood oxygenation. We compare the results of our oxygen model (cf. Fig 5C, case CMPR, root node geometry RC9) with predictions of a simplified model using a constant intravascular oxygen concentration.
(PDF)

S2 Fig Data for doubled parameter value of maximal O_2_ consumption rate *M*_0_. Correlations of volume (*Y*) and length (*S*) weighted blood oxygen saturations at increased metabolic rate of tissue oxygen consumption are compared with case BASE.
(PDF)
