# Supplementary material for: Computational Model for Tumor Oxygenation Applied to Clinical Data on Breast Tumor Hemoglobin Concentrations Suggests Vascular Dilatation and Compression
Source: PLoS One. 2016 Aug 22;11(8):e0161267. doi: 10.1371/journal.pone.0161267 (PMC4993476; doi:10.1371/journal.pone.0161267)
Supplement: S1 Appendix — Details on the derivation of the transvascular oxygen mass transfer coefficient γ are given, simulation results are compared with literature references, estimating the accuracy of our method, and finally the blood pressure at inlets and outlets p(BC)(r) is defined. (PDF) [file pone.0161267.s001.pdf]

S1 Appendix

Model details and validation

Transvascular oxygen mass transfer coefficient

We base the transvascular mass transfer coefficient γ on experimental and theoretical data [1, 2] for the Nusselt number which is related to γ by

$$\gamma = \frac{D_p \alpha_p}{2r} Nu, \tag{1}$$

where r is the vessel radius, and D_p and α_p are the diffusion coefficient and solubility of oxygen in blood plasma, respectively. As seen in Figure A, we use a phenomenological fit function which approximately describes the literature data [1, 2] for Nusselt numbers (assuming a blood oxygen saturation of 90%). The form of this function is

$$Nu(r) = p_2(1 - \exp(-r/p_1)), \tag{2}$$

with parameters p_2 and p_1. A best fit gives $p_1 = 8\ \mu m$ and $p_2 = 4.7$. This is a good approximation for small radii $r < 100\mu m$. For very large radii of several mm, which are not relevant here, we expect this approximation to fail because then a vessels may be considered as a large reservoir of oxygen, where the total transvascular flux is dominantly determined by the vessel surface area. Hence we expect Nu to become proportional to r for $r \gg p_1$ so that r cancels out of the flux density of extravasated oxygen $j_{tv} = D_p \alpha_p Nu/2r(P - P_t)$.

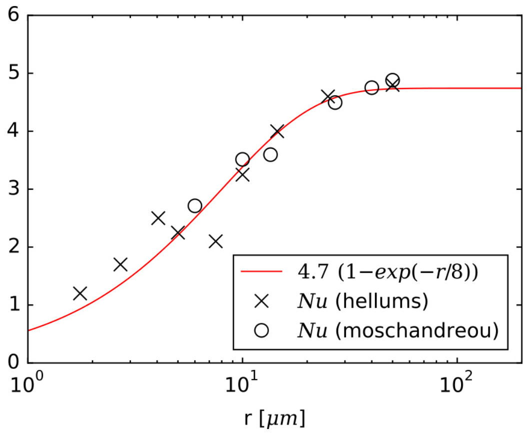

Figure A. Nusselt numbers: The dimensionless Nusselt number Nu describes the oxygen flux through the vessel wall in proportion to the difference between the average intravascular PO2 and the PO2 at the wall. $Nu(hellums)$ and $Nu(moschandreou)$ denote experimental [1] and theoretical data [2, Fig. 6], assuming a blood oxygen saturation of $S \approx 0.9$. The red curve is a best fit to the data.

Validation and comparison with literature data

To validate our model we considered the variation of intravascular PO2 distributions in systems comprising a single vascular tube which is embedded in a block of a medium through which oxygen can diffuse. We considered several cases with a tube length of $4\ mm$ and various radii, and compared these cases with literature data [2, 3] obtained from precise calculations with detailed models. In general we obtain fair agreement. For comparison Figure B shows blood oxygen saturations S at the end of the tube depending on tube radius r.

Details of the considered configurations are given in the following. The theoretical model of Ref. [2] incorporates a multi-layered vascular tube as source of oxygen and a surrounding infinite

tissue with a homogeneous background representing oxygen release from the capillary plexus. We augmented the tissue diffusion equation (15, s. main text) accordingly by adding the source term $\kappa - P_{t,i}\kappa/P^*$, where $\kappa = 0.625 mmHg$ and $\kappa/P^* = 30$. Other parameter which are already present in our model were readily adopted. The flow rate q poses an exception since it was not provided directly by Ref. [2]. We determined it according to [2, (A6)] using the velocity profile coefficients in the appendix. The simulation box was made large enough for the radial P_t profile to assume the asymptotic value ($42\ mmHg$) determined by κ and M_0. [3] considered a configuration where a vascular tube is surrounded by a block of rubber which is in contact with air at a specific oxygen partial pressure. Two model variants exist. One situation where oxygen rich blood releases oxygen to the rubber block [3, Fig. 2] and another where oxygen depleted blood takes up oxygen [3, Fig. 3] from it. In the former case the PO2 of the air is kept at $160\ mmHg$ and the blood PO2 at the inlet is 0. Vice versa for the former case. In either case, gas exchange happens through the surrounding rubber block. We accounted for this situation by Dirichlet boundary conditions and by setting the consumption rate $M_0 = 0$. The faces perpendicular to the longitudinal axis are imposed with no-flux conditions. The system size is set according to the reported half-thickness of the block as $4\,mm \times 80\,\mu m \times 80\,\mu m$. Computation were performed with a grid resolution of $h = 20\ \mu m$. The maximal relative error of the saturation obtained at the end of some tube $S(x = 4mm)$ amounts to approximately 16%.

Additionally, we compared our model with other numerical simulation methods using small vascular networks. [4] recently published the software that produced the results for the theoretical studies [5, 6] using a Green's function method. Using software provided by Ref. [4] we were limited to small networks confined within volumes of ca. $0.1\ mm^3$ due to the computational cost of the Green's function method. Exemplary simulation results can be seen in Figure C. Obtained PO2 distributions are in good agreement with results obtained by our method. We estimate a local worst case difference in the intravascular PO2, P, of 30%, and an average difference relative to the mean of P of ca. 16%.

Poor convergence of the solution with the grid constant h is a significant source of error in our method. Convergence tests with respect to the grid constant h show sublinear convergence as shown in Fig D where we plotted vascular and tissue oxygen partial pressures in a single vascular tube for different grid constants. After an initial settling length, the oxygen partial pressure behind the inlet is underestimated by an approximately constant offset, depending on h. The difference between the on-axis value of blood and tissue PO2 remains approximately spatially constant. Relative errors from the extrapolated true solution therefore start rather small behind the inlet (approx. 3%) and increase to 13% at the end. The reason for poor convergence is the singular nature of the oxygen source term (18) in the tissue oxygen diffusion equation , i.e. the use of the Dirac-δ function. D'Angelo [7] obtained better convergence by using locally refined meshes. Although it is a very challenging task, steps along those lines could be untertaken to improve numerical accuracy.

It is possible to arrive at inconsistent solutions when the iteration of (20) and (21) has not sufficiently converged. Then the amount of oxygen extracted from the network is not the same as the amount consumed by tissue. Hence, in order to check for conservation of mass we determined the total transvascular oxygen flux J_{tv}, the total consumption $J_{cons} = \int_\Omega M(P_t)\boldsymbol{dx}$ and considered the error $e_{tv} = (J_{tv} - J_{cons})/J_{tv}$. We also checked for mass conservation errors in the vasculature due to the numerical integration of P and determined $e_v = (J_{in} - J_{out} - J_{tv})/J_{in}$, where J_{in} and J_{out} are the influx and efflux through root nodes. We found both errors to be less than 1% at the point where further iteration does not change the solution any more.

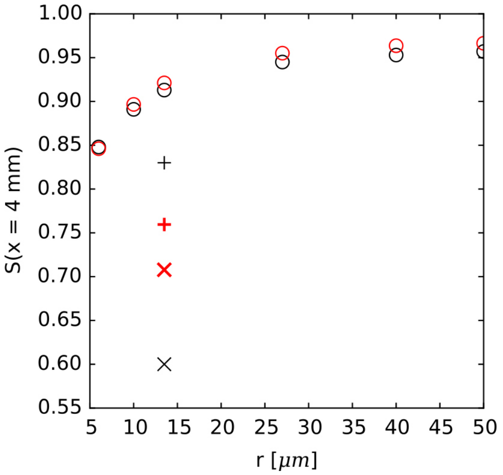

Figure B. Comparison of blood oxygen saturations at ends of vascular tubes: We considered a single $4\ mm$ long vascular tube embedded in a passive medium through which oxygen can diffuse. We computed the blood oxygen saturation S at the end of the vascular tube $x = 4\ mm$ (red) under various conditions (see text), such as varying vascular radii r, which is plotted here. Literature data of more sophisticated capillary models for equivalent cases [2, Fig. 4] ($\circ$), [3, Fig. 2] ($\times$), and [3, Fig. 3] ($+$) are shown as black symbols.

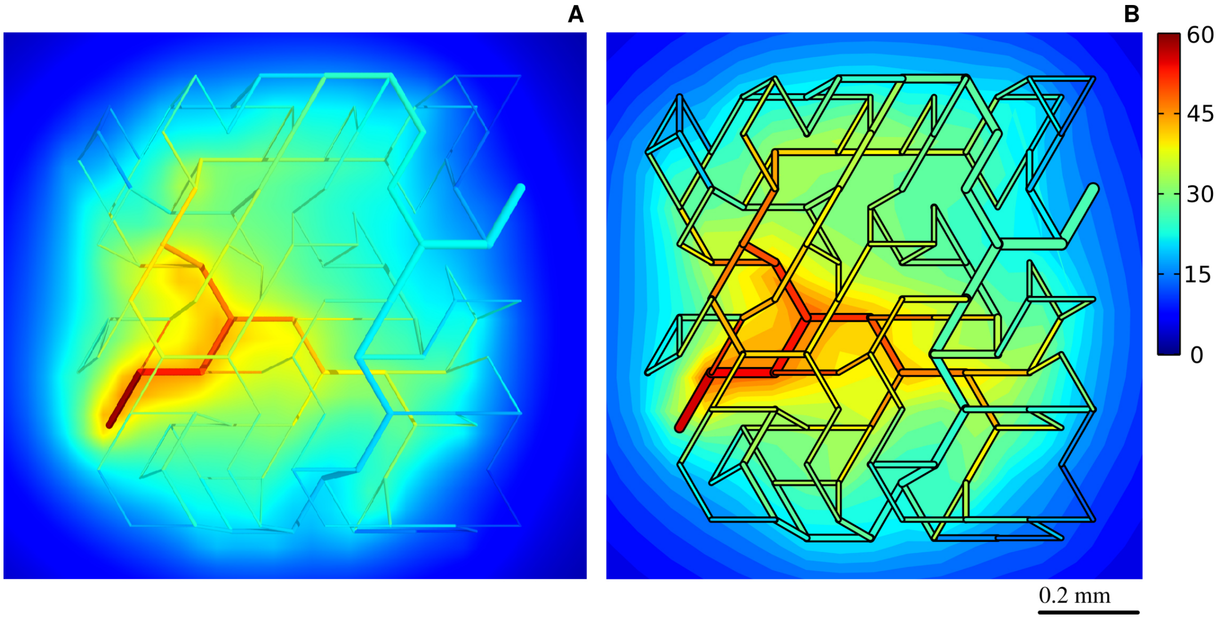

Figure C. Comparison with Green's function method: (A) shows the oxygen partial pressure PO2 distribution produced by our program. (B) shows the distribution produced by the software by [4]. The solution of vascular PO2 distribution P is layered on top of a slice through the tissue oxygen PO2 distribution P_t. The input to both programs was a small artificial network, identical parameters, and a spacing of tissue points of $h = 40\ \mu m$. However, boundary conditions for the tissue PO2 diffusion equations could not be matched. Therefore we extended the simulation box beyond the displayed region by $1\ mm$ on every side. The colorscale for P and P_t is given in $mmHg$.

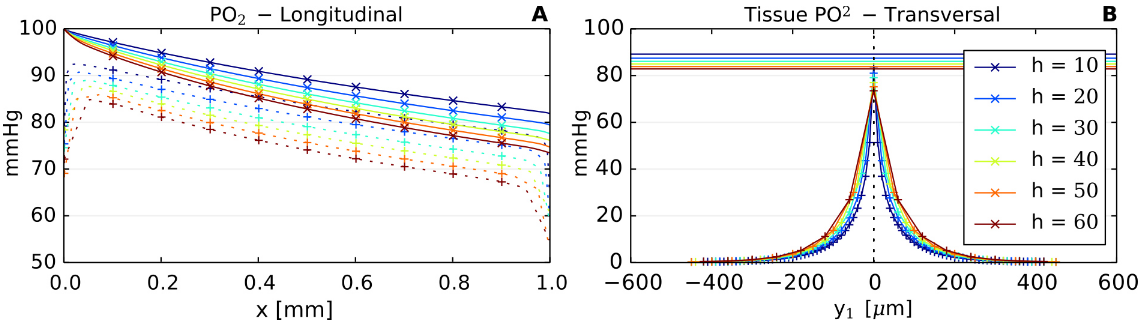

Figure D. Convergence test w.r.t. grid constant h. We consider a small simulation volume with a single vessel of length $1\ mm$ inside. Vascular (P, crosses) and tissue (P_t, pluses) oxygen partial pressure PO2 distributions are shown. The development of P and P_t (dotted line) along the vessel center axis is displayed in (A). In (B) we plotted the transversal profile of P_t, i.e. perpendicular to the vessel axis along the spatial coordinate y_1 at a point half way between the ends of the vessel. The horizontal lines indicate the vascular PO2 at this point. For smaller h the curves lie somewhat further apart, indicating sub-linear convergence, i.e. if the error to the true solution was linear in h we would observe a constant spacing between curves.

Blood Pressure Radius Relation

Blood flow boundary conditions of our model are such that the blood pressure is set to fixed numbers $p^{(BC)}$ for the solution for nodal pressures. Since blood pressure correlates well with vascular radius r, we let $p^{(BC)}$ be given as function of r and the type of vessel (arterial or venous). We first fit a curve to blood pressure data of rat mesentery [8]. Then we scaled the curve (adjusting $A1$ and $A2$) so that physiological blood pressures [9, Fig. 19.6] are obtain in venules and arterioles, thus obtaining for the blood pressure boundary condition

$$p^{(BC)}(r) = [A2 + (A1 - A2)/(1 + \exp((r' - r_0)/\Delta r))], \tag{3}$$

where $r' = r$ for a vein, else $-r$, and $A1 = 89\ mmHg$, $A2 = 18\ mmHg$, $r_0 = -21\mu m$, $\Delta r = 16\mu m$.

References

[1] Hellums JD, Nair PK, Huang NS, Ohshima N. Simulation of intraluminal gas transport processes in the microcirculation. Ann Biomed Eng. 1996 Jan-Feb;24(1):1–24. doi:10.1007/bf02770991.

[2] Moschandreou TE, Ellis CG, Goldman D. Influence of tissue metabolism and capillary oxygen supply on arteriolar oxygen transport: a computational model. Math Biosci. 2011 Jul;232(1):1–10. doi:10.1016/j.mbs.2011.03.010.

[3] Nair PK, Huang NS, Hellums JD, Olson JS. A simple model for prediction of oxygen transport rates by flowing blood in large capillaries. Microvasc Res. 1990 Mar;39(2):203–211. doi:10.1016/0026-2862(90)90070-8.

[4] Secomb TW; 2014. Available from: `http://www.physiology.arizona.edu/people/secomb/greens`.

[5] Secomb TW, Hsu R, Park EYH, Dewhirst MW. Green's Function Methods for Analysis of Oxygen Delivery to Tissue by Microvascular Networks. Annals of Biomedical Engineering. 2004 Nov;32(11):1519–1529. doi:10.1114/b:abme.0000049036.08817.44.

[6] Safaeian, N . Computational Modelling of Capillaries in Neuro-vascular Coupling. University of Canterbury; 2012. doi:10092/8038.

[7] D'Angelo C. Finite Element Approximation of Elliptic Problems with Dirac Measure Terms in Weighted Spaces: Applications to One- and Three-dimensional Coupled Problems. SIAM Journal on Numerical Analysis. 2012;50(1):194–215. doi:10.1137/100813853.

[8] Pries AR, Secomb TW, Gaehtgens P. Design principles of vascular beds. Circulation Research. 1995;77(1017-1022). doi:10.1161/01.res.77.5.1017.

[9] Marieb EN, Hoehn K. Human Anatomy & Physiology. Pearson; 2013.
