# Supplementary material for: Computational Model for Tumor Oxygenation Applied to Clinical Data on Breast Tumor Hemoglobin Concentrations Suggests Vascular Dilatation and Compression
Source: PLoS One. 2016 Aug 22;11(8):e0161267. doi: 10.1371/journal.pone.0161267 (PMC4993476; doi:10.1371/journal.pone.0161267)
Supplement: S2 Appendix — The simulation of tumor growth and oxygen PO2 distributions in blood and tissue yields vascular flow rates qv, hematocrit Hv, oxygen partial pressure P at discrete points on vessel axes, and tissue partial oxygen pressure Pt. In this section explicit formulas of other derived biophysical quantities are provided. (PDF) [file pone.0161267.s002.pdf]

S2 Appendix

Explicit Formulas for Relevant Biophysical Quantities

After vascular networks of normal tissue and tumor were constructed, distributions of hematocrit in vascular networks and oxygen concentrations in vascular networks and tissue are calculated, yielding for each vessel segment $v \in \mathbb{V}$ e.g. radius r_v , length l_v, hematocrit H_v, blood flow q_v, blood oxygen saturation $S_v(0)$ and oxygen concentration $c_v(0)$ at vessel entrance as well as average blood oxygen saturation S_v. Using this information, the following averages are calculated, pertaining to the tissue volume Ω.

Length density :

$$L_D = \frac{1}{|\Omega|} \sum_{v \in \mathbb{V}} l_v, \tag{1}$$

taken as measure for microvessel density MVD.

Regional blood volume (vascular volume density) :

$$rBV = \frac{1}{|\Omega|} \sum_{v \in \mathbb{V}} \pi r_v^2 l_v \tag{2}$$

Vascular surface density :

$$S_D = \frac{1}{|\Omega|} \sum_{v \in \mathbb{V}} 2\pi r_v l_v. \tag{3}$$

Regional blood flow (perfusion) :

$$rBF = \frac{1}{|\Omega|} \sum_{v \in \mathbb{I} \cap \partial\Omega} q_v = \frac{1}{|\Omega|} \sum_{v \in \mathbb{O} \cap \partial\Omega} q_v, \tag{4}$$

where $\mathbb{I} \cap \mathbb{V}$ ($\mathbb{O} \cap \mathbb{V}$) denote set of all vessel segments penetrating surface $\partial\Omega$ with blood flow pointing inwards (outwards).

Tissue hemoglobin concentration :

$$c_{Hb} = MCHC \cdot rRBCV = \frac{MCHC}{|\Omega|} \sum_{v \in \mathbb{V}} \pi r_v^2 l_v H_v \tag{5}$$

with mean corpuscular hemoglobin concentration $MCHC$, regional RBC volume $rRBCV$.

Tissue oxyhemoglobin concentration :

$$c_{HbO} = \frac{MCHC}{|\Omega|} \sum_{v \in \mathbb{V}} \pi r_v^2 l_v H_v \frac{1}{l_v} \int_v S_v(x) dx \tag{6}$$

and an analogous expression for the tissue deoxyhemoglobin concentration c_{HbD}, replacing $\frac{1}{l_v} \int_v S_v(x) dx$ by $1 - \frac{1}{l_v} \int_v S_v(x) dx$.

Tissue blood oxygen saturation :

$$Y = \frac{c_{HbO}}{c_{Hb}} = \sum_{v \in \mathbb{V}} \left\{ \frac{\pi r_v^2 H_v l_v}{\sum_{u \in \mathbb{V}} \pi r_u^2 l_u H_u} \frac{1}{l_v} \int_v S_v(x) dx \right\}, \tag{7}$$

where Y is the RBC-volume-weighted average of blood oxygen saturation taken over all vessel segments.

Length-weighted average blood oxygen saturation :

$$\langle S \rangle = \sum_{v \in \mathbb{V}} \frac{l_v}{\sum_{u \in \mathbb{V}} l_u} \frac{1}{l_v} \int_v S_v(x) dx \tag{8}$$

Average partial oxygen pressure in blood :

$$\langle P \rangle = \sum_{v \in \mathbb{V}} \frac{l_v}{\sum_u l_u} \frac{1}{l_v} \int_v P(x) dx, \tag{9}$$

where the average concentration of dissolved oxygen in blood is given by $\bar{c}_p = \alpha_p \langle P \rangle$.

Average tissue partial oxygen pressure :

$$P_t = \frac{1}{|\Omega|} \int_\Omega P_t(\boldsymbol{x}) d^3x, \tag{10}$$

where the average oxygen concentration in tissue is given by $\bar{c}_t = \alpha_t \langle P_t \rangle$.

Metabolic rate of oxygen consumption :

$$MRO_2 = \frac{1}{|\Omega|} \int_\Omega M(P_t(\boldsymbol{x})) d^3x, \tag{11}$$

where M is the Michaelis-Menten relation (17).

Total transvascular oxygen flux :

$$J_{tv} = \sum_{v \in \mathbb{V}} 2\pi r_v \gamma(r_v) \int_v \{P(x) - P_t(x)\} dx, \tag{12}$$

and corresponding regional transvascular oxygen flux $rJ_{tv} = J_{tv}/|\Omega| = MRO_2$.

Average transvascular oxygen flux density :

$$\langle j_{tv} \rangle = \frac{1}{\sum_{u \in \mathbb{V}} l_u} \sum_{v \in \mathbb{V}} \gamma(r_v) \int_v \{P(x) - P_t(x)\} dx, \tag{13}$$

i.e. the average transvascular oxygen flux density is the length weighted average over the mean oxygen flux density of all vessel segments; note that $S_D \langle j_{tv} \rangle \neq \frac{J_{tv}}{|\Omega|} = MRO_2$.

Oxygen flux entering vascular network :

$$J_{in} = \sum_{v \in \mathbb{I} \cap \partial\Omega} q_v \{c_0 H_v S_{in,v} + \alpha_p P_{in,v}\}, \tag{14}$$

with $P_{in,v} = P_v^{(BC)}$ and $S_{in,v}$ given by the Hill equation. The corresponding regional influx is $rJ_{in} = J_{in}/|\Omega|$.

Oxygen flux leaving vascular network :

$$J_{out} = \sum_{v \in \mathbb{O} \cap \partial\Omega} q_v \{c_0 H_v S_{out,v} + \alpha_p P_{out,v}\} \tag{15}$$

Oxygen extraction fraction :

$$OEF = \frac{J_{in} - J_{out}}{J_{in}} = MRO_2/rJ_{in}. \tag{16}$$
