# Supplementary material for: Computational Model for Tumor Oxygenation Applied to Clinical Data on Breast Tumor Hemoglobin Concentrations Suggests Vascular Dilatation and Compression
Source: PLoS One. 2016 Aug 22;11(8):e0161267. doi: 10.1371/journal.pone.0161267 (PMC4993476; doi:10.1371/journal.pone.0161267)
Supplement: S3 Appendix — In this section we derive an approximative relation between the number of root nodes and the regional blood volume, provided that the total number of vessels is held constant. Here we assume that all vascular trees are perfect binary trees. (PDF) [file pone.0161267.s003.pdf]

S3 Appendix

Regional Blood Volume of Confined Perfect Binary Trees

In this section we derive an approximative relation between the number of root nodes R and the regional blood volume rBV, provided that the total number of vessels M is held constant, i.e. all vascular trees are confined to the simulation box Ω and adhere to a fixed vascular density. Naturally R is also the number of trees. We assume that all vascular trees are perfect binary trees, i.e. tree levels from $k = 0$ at the top to $k = n - 1$ at capillary (leaf) level have the maximum number of vessels 2^k. Furthermore we assume that trees are equal in the number of vessels (connections) N. Hence $M = N \cdot R$ is split to equal parts between trees. There is no distinction between arterial and venous trees. For given number of levels (depth) n, a tree has $N = \sum_{k=0}^{n-1} 2^k$ connections. This is a geometric series for which holds in general

$$\sum_{k=0}^{n-1} q^k = \frac{q^n - 1}{q - 1}, \tag{1}$$

for some q. N can therefore be written as $N = 2^n - 1$, from which we obtain the depth

$$n = \log_2(N + 1), \tag{2}$$

Let further the radii at a bifurcation be given by Murray's law, i.e. $r_c^\alpha = r_a^\alpha + r_b^\alpha$. Since a and b are the same, we have $r_c = 2^{1/\alpha} r_{a=b}$, or in relation to the tree level k, we have the radius at level k

$$r_k = 2^{(n-1-k)/\alpha} r_{n-1}, \tag{3}$$

where r_{n-1} is the capillary radius. Now let's estimate the volume of the tree TV, provided equal vessel lengths l. By summing over tree levels we obtain

$$TV = l\pi \sum_{k=0}^{n-1} 2^k r_k^2, \tag{4}$$

since each level has 2^k vessels of radius r_k. Replacing r_k by Eq.(3) we obtain

$$TV = \pi r_{n-1}^2 l \cdot 2^{(n-1)2/\alpha} \sum_{k=0}^{n-1} 2^{k(1-2/\alpha)}. \tag{5}$$

This is again a geometric series which is rewritten with the help of Eq.(1) and $q = 2^{1-\frac{2}{\alpha}}$ to remove the sum. Furthermore without loss of generality we set $\pi r_{n-1}^2 l = 1$. After some basic algebra, the result is

$$TV = \frac{2^n - 2^{(2/\alpha)n}}{2 - 2^{2/\alpha}} \tag{6}$$

$$= \frac{(N+1) - (N+1)^{2/\alpha}}{2 - 2^{2/\alpha}}, \tag{7}$$

where in the second line we further used that $2^n = N + 1$. Now to satisfy the constraint that the total number of vessels taken over all trees is constant M, we substitute $N = M/R$, using the number of trees R. For brevity we set the volume of the simulation box $|\Omega| = 1$ without loss of generality. Thus we obtain the total regional volume of all trees rBV

$$rBV = \frac{TVR}{|\Omega|} \tag{8}$$

$$= M \frac{1 + \frac{R}{M} - \frac{R}{M}(\frac{M}{R} + 1)^{2/\alpha}}{2 - 2^{2/\alpha}}. \tag{9}$$

The denominator $2 - 2^{2/\alpha}$ is a positive increasing function of α that vanishes for $\alpha = 2$ and which asymptotically approaches 1 for $\alpha \to \infty$. It can be shown that the nominator is a decreasing

function in R, which is well approximated for small $R/M \ll 1$ by $1-(R/M)^{(1-2/\alpha)}$. In the edge case where $\alpha = 2$ we still obtain a decreasing function by calculating the limit of $\alpha \to 2$ for Eq.(6). In that case it is logarithmic with $rBV = \frac{1}{2}(R+M)\log_2(M/R+1)$.

Murrays α determines how much parent vessel radii grow at bifurcations. In the limit of $\alpha \to \infty$, Murrays law selects the radius of the thicker child vessel as the radius of the parent vessel. This means all radii become equal, and indeed $rBV(\alpha \to \infty) = M$ is obtained from Eq.(9). As we would intuitively expect, the dependence on R is eliminated. For realistic $2 \leq \alpha \leq 3$, the dependence on R cannot be removed. Moreover we can consider the case where all vessels are root vessels, i.e. $R = M$. Then again $rBV = M$ is obtained, which is clear since then again all vessels have equal radii. In actual simulations the assumption of perfect and equally sized trees does not hold, i.e. frequently, small branches are generated. Moreover, even if all lattice boundary sites are occupied with root nodes, only a small fraction of them grow to vascular trees of significant size. Most of them regress to a single starting node without any connections. Despite that, we observe a clear trend of decreasing rBV with increasing R.
