# Supplementary material for: Computational Model for Tumor Oxygenation Applied to Clinical Data on Breast Tumor Hemoglobin Concentrations Suggests Vascular Dilatation and Compression
Source: PLoS One. 2016 Aug 22;11(8):e0161267. doi: 10.1371/journal.pone.0161267 (PMC4993476; doi:10.1371/journal.pone.0161267)
Supplement: S4 Appendix — In support of our model we further discuss simulated data listed in Tables 3 and 4 in some detail, comparing our results with clinical data, data obtained from animal models and theoretical data available in the literature. (PDF) [file pone.0161267.s004.pdf]

S4 Appendix

Further details on comparison between simulated and literature biophysical data (see Tables 3 and 4)

In support of our model we further discuss simulated data listed in Tables 3 and 4 in some detail, comparing our results with clinical data, data obtained from animal models and theoretical data available in the literature.

Microvessel density MVD

The microvessel density MVD is measured microscopically by counting the number of microvessels per area of a tissue microscopic slide. The MVD is a quantity not easy to simulate. Therefore we take the simulated length density L_D , i.e. the total length of the vascular network divided by the tissue volume as measure for MVD (s. S2 Appendix). This definition yields the correct dimension and is identical to the number of vessels per cross-sectional area in case of a Krogh-like arrangement of parallel vessels. However, when comparing experimental MVD data and simulated length densities L_D it should be kept in mind that both parameters are not identical. In a large portion of the experimental literature on breast tumors, the MVD is obtained from well vascularized areas, so called hot-spots. Dhakal et al. [1] reported densities between 15-348 mm^{-2} with a mean of 88 mm^{-2}. Hot-spots are predominantly found near the tumor rim, but apparently not exclusively. Fernandez-Guinea et al. [2] reported somewhat lower MVD values of 0-157 mm^{-2} from breast tumor centers with a median of 20 mm^{-2}. Stamatelos et al [3] reconstructed the vasculature of a tumor of volume $280\,mm^3$ of a human breast cancer animal model and these authors report an average length density L_D of $21\,mm^{-2}$. However, spatial heterogeneity of the vasculature length density varied considerably within the experimental tumor investigated, ranging from about $9\,mm^{-2}$ up to $101\,mm^{-2}$. We obtain a whole tumor average of $133 \pm 18mm^{-2}$ (case CMPR, s. Table 3) which includes a significant contribution of the rim. The prediction of the MVD of the central part of the tumor agrees with the data of [2]. Microvessel density MVD of normal breast tissue is reproduced accurately.

Regional vessel volume rBV

The regional vessel volume rBV denotes the volume fraction of tissue occupied by vessels, i.e. vascular volume density (s. S2 Appendix). Using clinical data on hemoglobin concentrations of normal breast tissue ($c_{Hb} = 17.3 \pm 6.2$ $\mu mol/l$ [4]), and of tumors ($c_{Hb} = 53 \pm 32$ $\mu mol/l$ [4]), we estimated the vascular volume fraction. Tissue hemoglobin concentration c_{Hb} is the product of mean corpuscular hemoglobin concentration ($MCHC$) and regional RBC volume $rRBCV$ (s. S2 Appendix). Assuming hematocrit $H = 0.45$ to be homogeneously distributed within the vascular network, tissue hemoglobin concentration is given by $c_{Hb} = MCHC \cdot H \cdot rBV = c_{Hb,blood} \cdot rBV$, where $c_{Hb,blood}$ is the hemoglobin concentration in whole blood. Using $64.5kg/mol$ for the hemoglobin molar mass and assuming for the hemoglobin concentration in blood $c_{Hb,blood} = 14g/dl$ (women, normal range: $12 - 16$ g/dl), we estimate $rBV = 0.008 \pm 0.003$ (normal breast tissue) and $rBV = 0.024 \pm 0.015$ (breast tumors). Results on hemoglobin concentration of normal breast tissue and breast tumors obtained by a number of groups using optical mammography scatter by about a factor of two [4], resulting in an upper limit of $rBV = 0.016$ for normal breast tissue and a range of $rBV = 0.01$–0.06 for breast tumors. Stamatelos et al [3] report an average of tumor vessel volume density of 0.007 and rBV to vary within the tumor of the human breast cancer model from 0.002 up to 0.032. Beaney [5] obtained distinctly larger rBV estimates for human breast tissue ($rBV = 0.034$) and human breast tumors ($rBV = 0.043$), derived from PET measurements using the steady state $^{15}O_2$ inhalation technique. Note, however, that the rBV values reported by Beaney [5] for breast tumors, have to be considered as unreliable since the compartment model used to analyze the PET data is inaccurate for heterogeneous tissue [6]. For comparison, in normal human brain regional blood volume was determined from PET measurements to be $rBV = 0.038 \pm 0.007$ [7]. Using MRI and a brain tumor animal model yielded $rBV = 0.026 \pm 0.003$ (normal brain tissue) and $rBV = 0.053 \pm 0.006$ (brain tumor) [8]. Our simulation results on regional vessel volume of normal breast tissue and breast tumors (case CMPR) agree with clinical data derived from optical

mammography. Furthermore, rBV of breast tumors (case CMPR) falls within the range of volume densities reported in [3] for the animal breast cancer model. In addition, for normal breast tissue, we deduce a venous fraction of the total blood volume fraction $vrBV/rBV = 68\%$ in fair agreement with MRI data of normal human brain (77%) [9]. Because the classification of vessel segments of the tumor vasculature into arterioles, capillaries and venules no longer applies, the venous fractional blood volume is meaningless in case of tumors.

Vessel surface to vessel volume

Besides vascular surface density S_D, Table 3 lists the vessel surface density to vessel volume density ratio S_D/rBV. For a single vessel segment vessel surface to vessel volume scales as $1/r$. Although this result will no longer be exactly applicable for the ratio of vessel surface density to vessel volume density, we expect this ratio to decrease with increasing average vessel radius. For tumor vasculature (case CMPR) we find $S_D/rBV = 0.27\ \mu m^{-1}$ close to the simulated value for normal breast tissue ($S_D/rBV = 0.29\ \mu m^{-1}$). This modest decrease is ultimately explained by the low maximal dilation radius $r^{(max)} = 14\ \mu m$, leading to an average vascular radius of $r = 5.2\ \mu m$ (case CMPR), only $2\ \mu m$ above the average radius of initial networks. A contributing factor is the peripheral plexus of thin and densely packed vessel (high S_D/rBV), part of which is included in the tumor volume. Stamatelos et al [3] reported a surface to volume ratio of $S_D/rBV = 0.17\ \mu m^{-1}$ for a human breast tumor animal model together with an average (median) vessel radius of $r = 9.2\ \mu m$.

Perfusion

Dynamical PET measurements ($^{15}O_2$) on 37 breast cancer patients yielded $rBF = 0.06\, ml/g/min$ (mean, normal tissue, range $0.03 - 0.17\, ml/g/min$) and $rBF = 0.32 ml/g/min$ (mean, tumor, range $0.08 - 0.9\, ml/g/min$) [10]. Nearly the same results were reported in [11]. Volumes of human breast cancers imaged range from one to several cm^3, typically. On the other hand, the range of simulated regional blood flow $rBF = q_{sph,tum}/|\Omega_{sph}|$ of the small ($0.014\ cm^3$) spherical tumor amounts to $0.13 - 7.5 ml/g/min$, with a mean $1.25 ml/g/min$ (case CMPR) being higher by about a factor of 4 compared to the clinical results (s. Table 3). We attributed the discrepancy between simulated perfusion and clinical data to the considerable difference in tumor volumes. Indeed, a general decrease of perfusion with increasing tumor mass was seen previously for human breast cancer xenografts. Perfusion ranged from $0.5\ ml/g/min$ at $m_{tum} = 0.4\ g$ down to $0.025\ ml/g/min$ at $m_{tum} = 8\ g$ [12] . For eliminating the dependence of simulated perfusion on tumor volume, Table 3 lists the scaled perfusion $rBF_{scaled} = rBF_{norm} \cdot q_{sph,tum}(t = 600h)/q_{sph,norm}(t = 0)$. Mean simulated scaled perfusion rBF_{scaled} (case CMPR) is lower by about a factor of 2 compared to the clinical results, possibly overestimating size dependence of tumor perfusion. Our results for normal breast tissue (range: $0.032 - 0.064 ml/ml/min$) agree well with clinical data.

Wall shear stress

Simulated wall shear stress of normal vasculature falls within the range of shear stresses measured for human conjunctival arterioles, capillaries and venoles [13, 14]. Simulated shear stresses of the tumor model variants are consistent with shear stresses reported by Stamatelos et al [3] for a human breast cancer animal model.

Tissue oxygen partial pressures

The global ensemble average of simulated PO2 of normal tissue and tumor tissue amounts to $36 \pm 6\ mmHg$ and $29 \pm 13\ mmHg$, respectively (s. Table 4). Polarographic PO2 measurements at a large number of sites in normal breast tissue and breast tumors of 15 patients yielded median PO2 values of $65\ mmHg$ and $30\ mmHg$, respectively [12]. The simulated PO2 value of tumor tissue (case CMPR) reproduces the clinical result exactly, the coincidence possibly being fortuitous, considering the skewed histogram of measured partial oxygen pressures [12]. Since the distribution of measured partial oxygen pressures in normal breast tissue is rather broad, simulated and measured tissue partial oxygen pressures in normal tissue can be considered to agree.

Metabolic rate of oxygen consumption

Our model assumes tissue metabolic rate of oxygen consumption to follow a Michaelis-Menten relation and takes the maximal rate M_0 and half-pressure P_{M50} as input. In particular, maximal rate M_0 in tumor tissue was assumed to be four times larger than in normal breast tissue, whereas half-pressure P_{M50} of normal breast tissue was adopted to be twice that of breast cancers (s. Table 1). Since (average) partial oxygen pressures in normal and tumorous breast tissue were simulated to be considerably higher than the assumed half-pressures P_{M50}, the simulated metabolic rates $MRO_2 = 3.25\ \mu l O_2/ml/min$ (normal tissue) and $MRO_2 = 12.1\ \mu l O_2/ml/min$ (breast tumor, case CMPR) are essentially determined by the adopted parameter M_0 of the Michaelis-Menten relation. From PET measurements based on the steady-state $^{15}O_2$ inhalation technique, Beaney [5] reported $MRO_2 = 4.5\ \mu l O_2/ml/min$ and $MRO_2 = 6.6\ \mu l O_2/ml/min$ for normal breast tissue and breast tumors, respectively. The latter value had been challenged because of tumor inhomogeneity [6]. Recently, a considerably higher metabolic rate of oxygen consumption $M_0 = 40.6 \pm 7.8\,\mu l\ O_2/ml/min$ was reported of viable multicellular human colorectal carcinoma spheroids, taken as in-vitro tumor model [15]. In addition, measurements of oxygen flux above a monolayer of cancer cells in culture medium yielded metabolic rates M_0 from $21\,\mu l\ O_2/ml/min$ up to $52\,\mu l\ O_2/ml/min$ for various human cancer cells, with $M_0 = 33.4 \pm 8.3\,\mu l\ O_2/ml/min$ for human breast cancer cells MDAMB- 468 [16]. For comparison we note that for normal human brain PET measurements yielded $CMRO_2 = 33 \pm 5\,\mu l\ O_2/ml/min$ [7].

Oxygen extraction fraction

Average blood oxygen saturation is (negatively) correlated with oxygen extraction fraction $OEF = J_{tv}/J_{in} = (J_{in} - J_{out})/J_{in} = MRO_2/rJ_{in}$, where J_{tv} is the total transvascular oxygen flux into tissue, in $J_{in}(J_{out})$ the total oxygen influx (efflux) into the vasculature (leaving the vasculature) of the tissue volume and rJ_{in} is the oxygen influx per unit volume of tissue considered. For the whole simulation box, J_{in} and J_{out} are determined by summing oxygen flux qc over all root arteries or veins, respectively. For the tumor, we sum qc over vessels penetrating the tumor surface with a flow direction into or out of the tumor. For normal breast tissue and breast tumors (case CMPR) we obtain the result $OEF = 0.34 \pm 0.1$ and $OEF = 0.11 \pm 0.09$, respectively. Oxygen extraction data on normal breast tissue and breast tumors are scarce in the literature. Beaney [5] reported the oxygen extraction fraction of normal breast tissue to be $OEF = 0.65 \pm 0.1$. This result seems to be rather high considering the oxygen extraction of normal human brain measured by PET to be $OEF = 0.44 \pm 0.06$ [7].

Using the steady state $^{15}O_2$ inhalation technique almost the same oxygen extraction fractions were measured for breast tumors ($OEF = 0.23 \pm 0.08$, [5]) and brain tumors ($OEF = 0.22 \pm 0.04$, [17]) , however the same reservations remain concerning inadequate data analysis because of tissue heterogeneity. Our simulations predict for breast tumors (case CMPR) oxygen extraction fractions of $OEF = 0.11 \pm 0.09$, an estimate likely to be too low. Since $MRO_2 = \epsilon \cdot c_{Hb,blood} S_{in} \cdot rBF \cdot OEF$, where ϵ is the Hüfner factor and S_{in} the arterial blood oxygenation of the vessels penetrating the surface of the spherical tumor, overestimation of the tumor regional blood flow will cause an underestimation of the oxygen extraction fraction OEF, since the metabolic rate of oxygen consumption is essentially fixed by the input parameters of the model. Conversely, estimates of scaled perfusion are probably too low, overestimating the dependence of perfusion on tumor volume. Therefore, corresponding oxygen extraction fractions of breast tumors are probably on the high side ($OEF = 0.66$, CMPR). In addition, Table 4 lists the oxygen influx J_{in} and average transvascular oxygen flux density j_{tv}, where we write j_{tv} informally for the average over the network length (s. S2 Appendix).

Tissue blood oxygen saturation

Vessel segments contribute to tissue hemoglobin concentration c_{Hb} (tissue oxyhemoglobin concentration c_{HbO}) according to their blood hemoglobin concentration, (blood oxyhemoglobin concentration) weighted by their fractional volume. In order to simulate c_{HbO} for each vessel segment its average blood oxygenation has to be known. Simulated tissue blood oxygen saturation $Y = c_{HbO}/c_{Hb}$ agree well with clinical data.

References

[1] Dhakal HP, Bassarova A, Naume B, Synnestvedt M, Borgen E, Kaaresen R, et al. Breast carcinoma vascularity: a comparison of manual microvessel count and Chalkley count. Histol Histopathol. 2009 Aug;24(8):1049–1059.

[2] Fernandez-Guinea O, Alvarez-Cofino A, Eiro N, Gonzalez LO, del Casar JM, Fernandez-Garcia B, et al. Low microvascular density at the tumor center is related to the expression of metalloproteases and their inhibitors and with the occurrence of distant metastasis in breast carcinomas. Int J Clin Oncol. 2013 Aug;18(4):629–640. doi:10.1007/s10147-012-0428-2.

[3] Stamatelos SK, Kim E, Pathak AP, Popel AS. A bioimage informatics based reconstruction of breast tumor microvasculature with computational blood flow predictions. Microvasc Res. 2014 Jan;91:8–21. doi:10.1016/j.mvr.2013.12.003.

[4] Grosenick D, Wabnitz H, Moesta KT, Mucke J, Schlag PM, Rinneberg H. Time-domain scanning optical mammography: II. Optical properties and tissue parameters of 87 carcinomas. Phys Med Biol. 2005 Jun;50(11):2451–2468. doi:10.1088/0031-9155/50/11/002.

[5] Beaney RP. Positron emission tomography in the study of human tumors. Semin Nucl Med. 1984 Oct;14(4):324–341. doi:10.1016/s0001-2998(84)80006-9.

[6] Lammertsma AA, Jones T. Low oxygen extraction fraction in tumours measured with the oxygen-15 steady state technique: effect of tissue heterogeneity. The British Journal of Radiology. 1992;65(776):697–700. doi:10.1259/0007-1285-65-776-697.

[7] Ito H, Kanno I, Kato C, Sasaki T, Ishii K, Ouchi Y, et al. Database of normal human cerebral blood flow, cerebral blood volume, cerebral oxygen extraction fraction and cerebral metabolic rate of oxygen measured by positron emission tomography with 15O-labelled carbon dioxide or water, carbon monoxide and oxygen: a multicentre study in Japan. Eur J Nucl Med Mol Imaging. 2004 May;31(5):635–643. doi:10.1007/s00259-003-1430-8.

[8] Christen T, Lemasson B, Pannetier N, Farion R, Remy C, Zaharchuk G, et al. Is T2* enough to assess oxygenation? Quantitative blood oxygen level-dependent analysis in brain tumor. Radiology. 2012 Feb;262(2):495–502. doi:10.1148/radiol.11110518.

[9] An H, Lin W. Cerebral venous and arterial blood volumes can be estimated separately in humans using magnetic resonance imaging. Magn Reson Med. 2002 Oct;48(4):583–588. doi:10.1002/mrm.10257.

[10] Mankoff DA, Dunnwald LK, Gralow JR, Ellis GK, Charlop A, Lawton TJ, et al. Blood flow and metabolism in locally advanced breast cancer: relationship to response to therapy. J Nucl Med. 2002 Apr;43(4):500–509. Available from: http://jnm.snmjournals.org/content/43/4/500.abstract.

[11] Wilson CB, Lammertsma AA, McKenzie CG, Sikora K, Jones T. Measurements of blood flow and exchanging water space in breast tumors using positron emission tomography: a rapid and noninvasive dynamic method. Cancer Res. 1992 Mar;52(6):1592–1597. Available from: http://cancerres.aacrjournals.org/content/52/6/1592.abstract.

[12] Vaupel P, Schienger K, Knoop C, Hockel M. Oxygenation of human tumors: Evaluation of tissue oxygen distribution in breast cancers by computerized O2 tension measurements. Cancer Research. 1991 Jun;51(12):3316–3322.

[13] Koutsiaris AG, Tachmitzi SV, Batis N. Wall shear stress quantification in the human conjunctival pre-capillary arterioles in vivo. Microvasc Res. 2013 Jan;85:34–39. doi:10.1016/j.mvr.2012.11.003.

[14] Koutsiaris AG, Tachmitzi SV, Batis N, Kotoula MG, Karabatsas CH, Tsironi E, et al. Volume flow and wall shear stress quantification in the human conjunctival capillaries and post-capillary venules in vivo. Biorheology. 2007;44(5-6):375–386. Available from: http://content.iospress.com/articles/biorheology/bir470.

[15] Grimes DR, Kelly C, Bloch K, Partridge M. A method for estimating the oxygen consumption rate in multicellular tumour spheroids. J R Soc Interface. 2014 Jan;11(92):20131124. doi:10.1098/rsif.2013.1124.

[16] Grimes DR, Kannan P, McIntyre A, Kavanagh A, Siddiky A, Wigfield S, et al. The Role of Oxygen in Avascular Tumor Growth. PLOS ONE. 2016 Apr;11(4):e0153692. Available from: http://journals.plos.org/plosone/article?id=10.1371/journal.pone.0153692. doi:10.1371/journal.pone.0153692.

[17] Leenders KL. PET: blood flow and oxygen consumption in brain tumors. J Neurooncol. 1994;22(3):269–273. doi:10.1007/bf01052932.
