# Supplementary material for: Computational Model for Tumor Oxygenation Applied to Clinical Data on Breast Tumor Hemoglobin Concentrations Suggests Vascular Dilatation and Compression
Source: PLoS One. 2016 Aug 22;11(8):e0161267. doi: 10.1371/journal.pone.0161267 (PMC4993476; doi:10.1371/journal.pone.0161267)
Supplement: S1 Fig — We compare the results of our oxygen model (cf. Fig 5C, case CMPR, root node geometry RC9) with predictions of a simplified model using a constant intravascular oxygen concentration. (PDF) [file pone.0161267.s005.pdf]

S1 Figure

Tissue PO2 distributions at constant vascular blood oxygenation

For some applications, such as models of tumor growth, it might be sufficient to implement a simplified model of oxygenation where the intravascular PO2 is a constant input parameter to the model. Results obtained from such a model are presented in the following. To this end we modified our present model, omitting the propagation of oxygen losses through the vascular network, and instead setting the oxygen partial pressure P to a constant. A map of the resulting oxygen distribution is shown here in Fig. A, where we set $P = 39\ mmHg$, equal to the mean intravascular PO2 taken over initial $(t = 0)$ networks of the simulated cohort as it is obtained from the full model. An obvious qualitative difference is the absence of long ranged P_t variations in normal tissue. Instead, fine grained fluctuations in-between neighboring vessels are much more apparent. As expected, the P_t level along the surviving tumor vessels is nearly constant so that the P_t distribution can approximately be described by a function of the distance to the closest vessel alone. The neglect of varying intravascular PO2, leads to overestimation of P_t in the peripheral region (cf. Fig. 5 C). Naturally, this simplified model implies constant tissue oxygen saturation Y, and thus does not allow to analyze experimental data on blood oxygen saturation in normal and tumorous tissue.

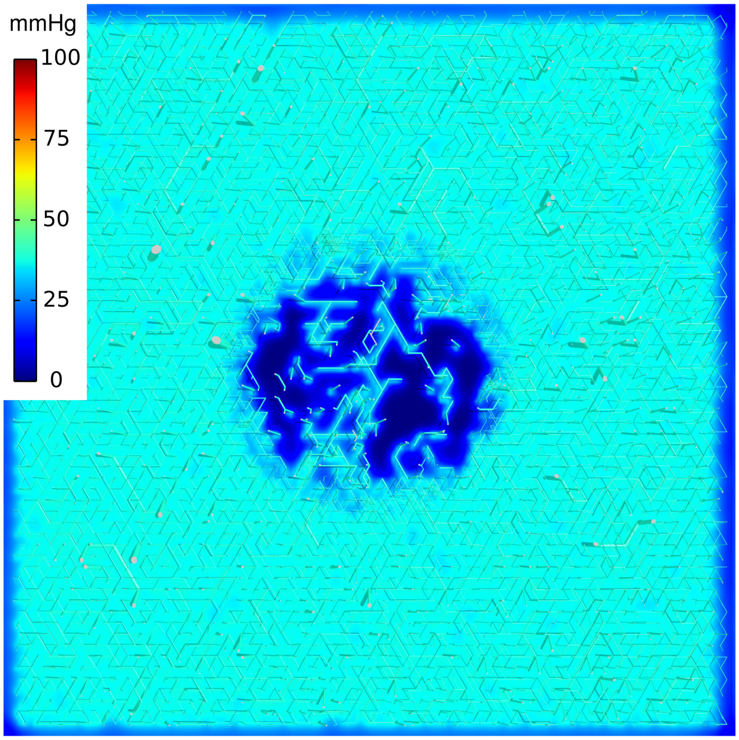

Figure A. Tissue oxygen distribution at constant vascular PO2: This figure shows a visualization of the network depicted in Fig 5 C,D but with blood PO2 held constant.
