# Supplementary material for: Computational Model for Tumor Oxygenation Applied to Clinical Data on Breast Tumor Hemoglobin Concentrations Suggests Vascular Dilatation and Compression
Source: PLoS One. 2016 Aug 22;11(8):e0161267. doi: 10.1371/journal.pone.0161267 (PMC4993476; doi:10.1371/journal.pone.0161267)
Supplement: S2 Fig — Correlations of volume (Y) and length (S) weighted blood oxygen saturations at increased metabolic rate of tissue oxygen consumption are compared with case BASE. (PDF) [file pone.0161267.s006.pdf]

S2 Figure

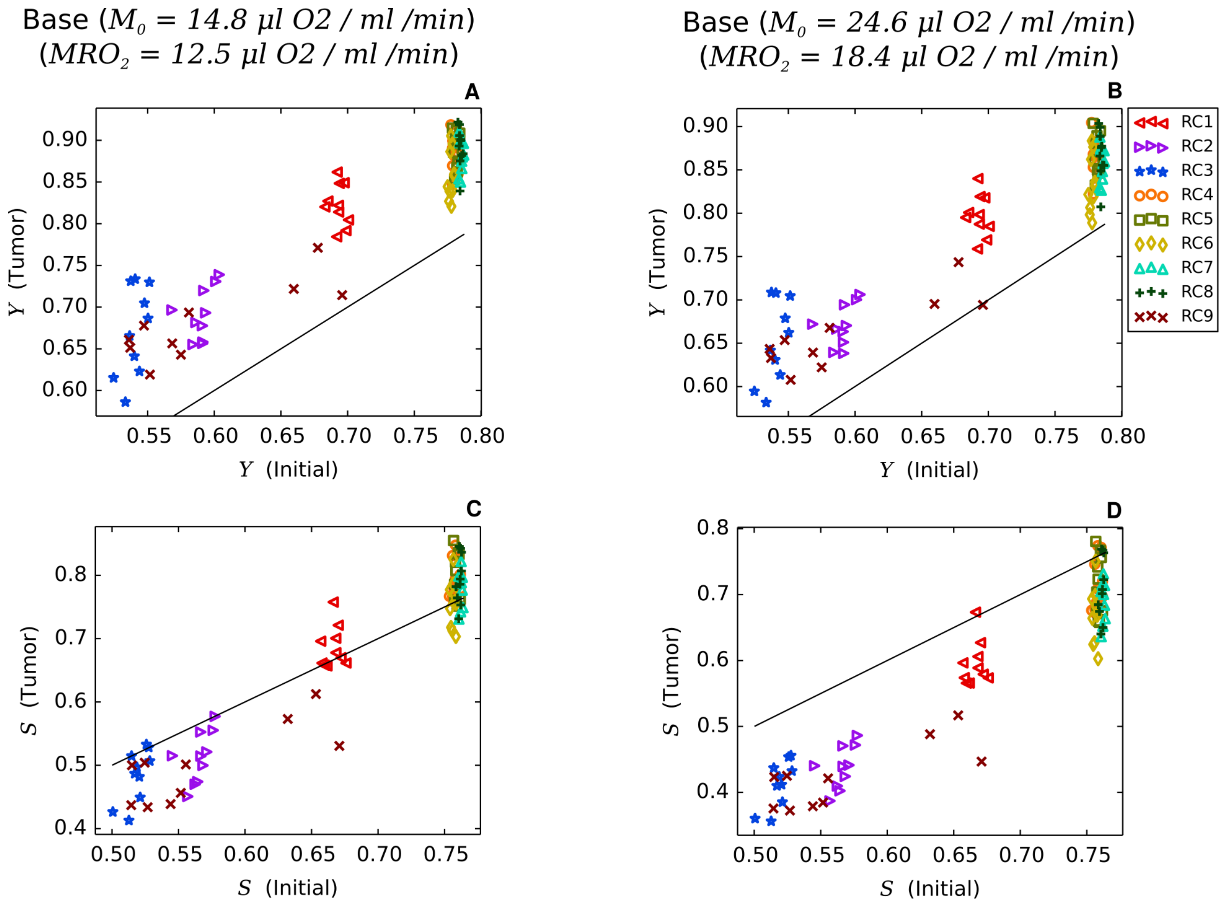

Figure A. Correlations of tissue blood oxygenation Y and length weighted blood oxygen saturation S for two assumed Michaelis-Menten metabolic rates of oxygen consumption M_0 (case BASE): Tissue blood oxygen saturation of tumors versus host tissue (A) and length-weighted blood oxygen saturation S of tumors versus host tissue (C) at $M_0 = 14.8\,\mu l\ O_2/ml/min$ (case BASE); right column: tissue blood oxygen saturation of tumors versus host tissue (B) and length-weighted blood oxygen saturation S of tumors versus host tissue (D) at elevated $M_0 = 24.6\,\mu l\ O_2/ml/min$; the color code to identify root node geometry is the same as in Fig 9.
